# Temporal prediction captures retinal spiking responses across animal species

**DOI:** 10.1038/s41467-026-77399-y

**Published:** 2026-09-08

**Authors:** Luke Taylor, Friedemann Zenke, Andrew J. King, Nicol S. Harper

**Affiliations:** 1 Department of Physiology, Anatomy and Genetics, University of Oxford, Oxford, UK; 2 Friedrich Miescher Institute for Biomedical Research, Basel, Switzerland; 3 Faculty of Science, University of Basel, Basel, Switzerland

**Keywords:** Retina, Network models, Biophysical models

## Abstract

The retina’s role in visual processing has been viewed as two extremes: an efficient compressor of incoming visual stimuli, akin to a camera, or a predictor of future stimuli. Addressing this dichotomy, we developed a spiking neural network model of the retina trained on natural movies under metabolic-like constraints to either encode the present or to predict future scenes. When optimized for efficient temporal prediction ~100 ms into the future, the model not only captures retina-like receptive fields and their mosaic-like organizations, but also exhibits complex retinal processes such as latency coding, motion anticipation, differential motion tuning, and stimulus-omission responses. Notably, the temporal prediction model also accurately predicts the way retinal ganglion cells respond across different animal species to natural images and movies. Our findings suggest that the retina is not merely a compressor of visual input, but rather is fundamentally organized to provide the brain with foresight into the visual world.

## Abstract (web summary)

Functional roles of retina in visual processing are not fully understood. This paper shows that a spiking neural network model trained to efficiently predict future visual input captures many features of retinal activity and does so better than one trained to efficiently encode only the current visual input.

## Introduction

Retinal ganglion cells (RGCs), driven by the photoreceptors via bipolar cells, respond to different patterns of light^1,2^ and send representations of the visual world to the brain^3^. Despite extensive progress, it remains an open question what general principle, if any, governs the information this collective pattern of activity represents. One hypothesis posits that the retina compresses the information in incoming light into an efficient neural code^4–10^, much like a camera capturing a visual scene. Another hypothesis is that the retina efficiently signals features within the visual input that are predictive of future input^11–15^. Such predictive features are argued to be advantageous for guiding actions in response to visual cues, capturing underlying variables and eliminating unnecessary information^16,17^. The concept of prediction is thought to be fundamental to many aspects of sensory processing, where the predominant focus has been on the role of cortical circuits in predicting sensory inputs^16–19^.

Normative modeling is a commonly used approach for exploring such coding questions^20,21^. This involves optimizing a model (in our case of the retina) for a particular goal, for example efficient compression or prediction, and comparing the resulting phenomena within the model to the biology. Previous normative models of the retina have mostly explored the efficient compression of natural images by employing model units that output firing rates^4–8^. These models have successfully captured the different types of retina-like spatial receptive fields (RFs) and their mosaic-like organizations. However, they lack particular biological features, such as the spiking responses of RGCs and their recurrent interactions^22^. Modeling spikes is important since RGCs may encode visual stimuli using the relative timing of spikes, which therefore play an important role in sensory transmission^23^. Furthermore, these models have not been shown to capture more complex retinal processes, such as stimulus-omission^24,25^ and motion-anticipation^26^ responses or differential motion tuning^27^. Thus, pivotal to the compression versus prediction dichotomy, it remains unclear to what extent a spiking model optimized for prediction compares to the retina.

To address these shortcomings, we developed a spiking retinal model, trained on natural movies under metabolic-like constraints, to either encode the present or to predict the future from recent spike activity. Like prior non-spiking normative investigations^4–8,15^, we found that our spiking model optimized for efficient temporal prediction was able to capture retina-like RFs and, to a degree, their spatial organizations. Extending beyond these results, we found that the model also exhibited retina-like latency coding^23^ and spike statistics in response to natural images and movies. Furthermore, the temporal prediction model captured complex retinal processes of motion tuning and sensitivity to the unexpected absence of a stimulus (stimulus-omission responses)^24,25^. In contrast, the model optimized for efficient compression did not account for complex retinal processes, like stimulus-omission responses. Notably, we found that the model optimized for prediction ~100 ms into the future more accurately predicted RGC responses across different animal species to both natural images and movies.

Although other principles, such as efficient compression, may also be factors underpinning the anatomy and physiology of the retina, this study demonstrates the broad explanatory capacity of the principle of temporal prediction, which can account for a remarkably diverse range of RGC properties across different species. Our findings therefore suggest that the retina in mammals and amphibians has evolved to supply the brain with representations of temporally predictive features of the visual world.

## Results

### The spiking retinal model

**Fig. 1:**
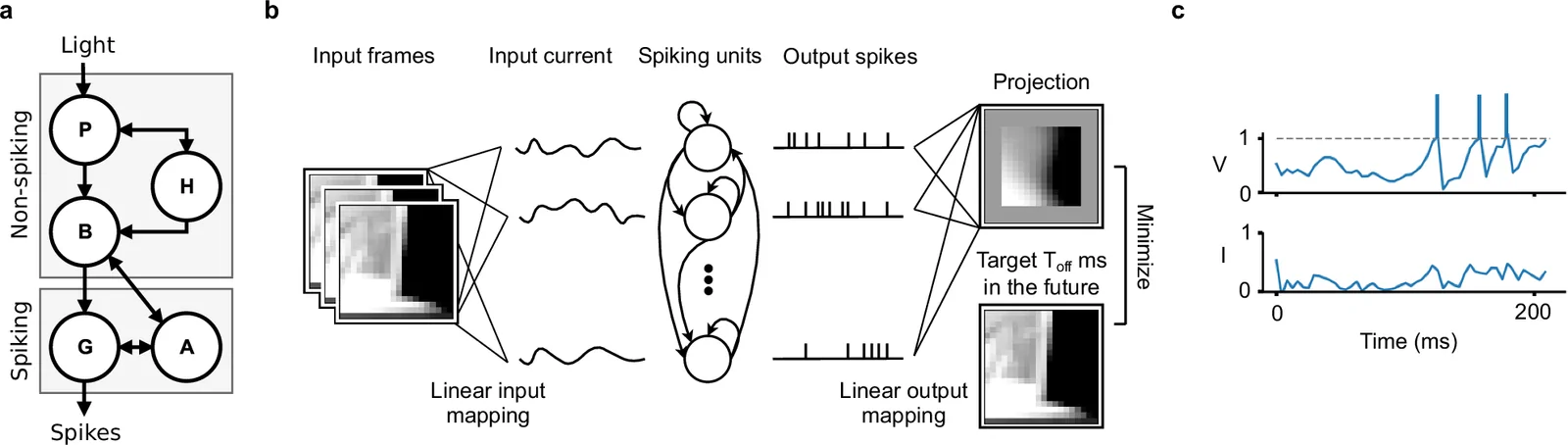
The spiking retinal model. **a** Schematic of the retinal circuit with its different cell types (P photoreceptor, H horizontal cell, B bipolar cell, A amacrine cell, G ganglion cell) and their respective connections, with the arrowheads indicating the flow of visual information. **b** Schematic of the retinal model. The model takes a sequence of movie frames as input and linearly transforms them into an input current that is fed into a single hidden layer of recurrently connected spiking LIF units. The linear transformation approximates the transformation by the pre-ganglionic cells in the top gray-edged box in (**a**), and the recurrently connected spiking units model the ganglion cells and their interactions with the amacrine cells, in the bottom gray-edged box. Photoreceptor-like noise was added to the input image, and ganglion-cell-like noise was added to the input current. The spike output of the model units was used to linearly construct a projection, either of the present or the future, of the input stimulus. **c** Example activity traces of one of the LIF units over time: input current *I* charges the membrane potential *V* and elicits a spike if the firing threshold (dashed line) is crossed.

The retina consists of three cellular layers: the photoreceptors (which transduce light), the bipolar cells (an intermediate cell layer), and the RGCs (the spiking output layer), interspersed by the horizontal and amacrine cell inhibitory interneurons^22^ (Fig. 1a). We modeled this circuit using a single hidden layer of recurrently connected spiking units (Fig. 1b), where the spiking units are equivalent to the RGCs. All spiking units were modeled using the leaky integrate-and-fire (LIF) model^28^, which captures the key biophysical nature of real neurons: integrate incoming current, and fire a spike when the firing threshold is exceeded (Fig. 1c). Pre-ganglionic cells (the photoreceptors, horizontal and bipolar cells) appear to respond to incoming light in a mostly linear fashion^29^. We thus opted to model their transformation using a linear mapping, whose output was fed as input current to the RGC-like spiking units. Similar to prior modeling work^30^, we included recurrent connectivity to capture the interactions between RGC types, arising from electrical synapses between RGCs^31,32^ and electrical and chemical synaptic connections between RGCs via amacrine cells^22,31,32^.

We also noise-perturbed the input stimulus and each unit’s membrane potential with Gaussian noise, to capture the noise characteristics of the photoreceptors and RGCs^33,34^. We set these noise sources so that the model’s spike train variability resembled that of the RGCs across repeated stimulus presentations of uniform full-field illumination with randomly varying intensity^3,35^, being sub-Poisson with Fano factors mostly below unity^35–37^ (Supplementary Fig. S1).

The model was trained on diverse movies of dynamic natural scenes, with the objective of estimating a target frame *T*_off_ ms into the future, or at the present, using a linear readout from past spike activity. We explored different prediction offset values *T*_off_ to examine whether a non-predictive encoding (*T*_off_ = 0 ms) or prediction (*T*_off_ > 0 ms) objective better captures the sensory processing of the retina. Apart from the different prediction offsets *T*_off_, the non-predictive encoding and prediction models had exactly the same architectures. We regularized the models’ connectivity during training using a metabolic-like cost function, which penalized individual synaptic-like connections by their cost in energy, approximated as the absolute value of a connection weighted by its incoming activity over time (see “Methods”). Unless otherwise mentioned, for the temporal prediction model, we report our results using the model optimized for prediction of *T*_off_ = 128 ms into the future, as this value enables neural responses to natural scenes to be captured well across multiple species (see below).

### Retina-like spike statistics to natural images and movies

**Fig. 2:**
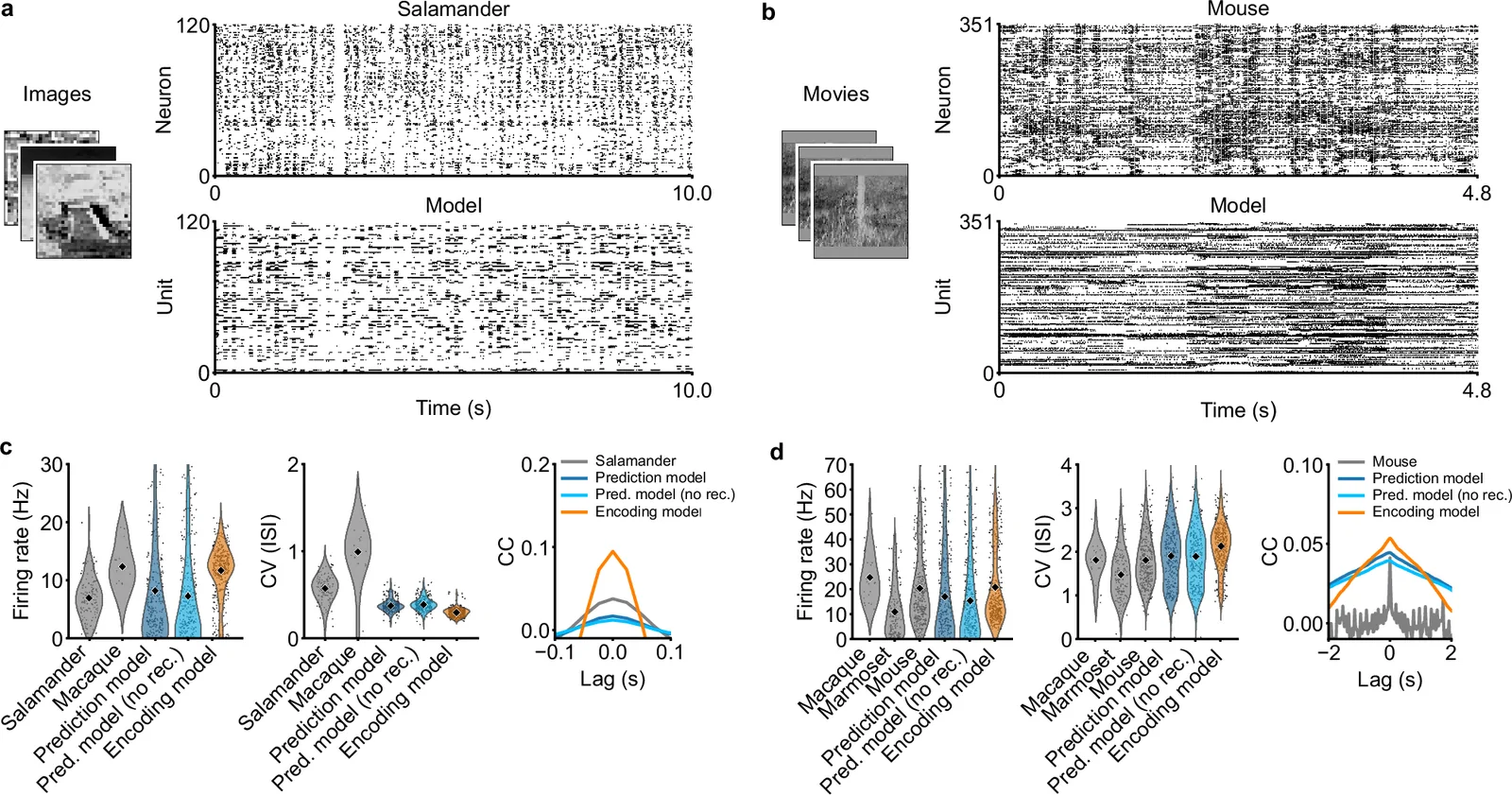
Spike responses to natural images and movies. **a** Raster plot from the salamander retina (top) and the prediction model (bottom) showing responses to the same natural images (left). **b** Raster plot from the mouse retina (top) and the prediction model (bottom) to the same natural movies (left). **c** Left: Violin plots showing the distribution of mean firing rates evoked by natural images for RGCs from different species (gray) and for the model units from the prediction model (dark blue), the prediction model with recurrence ablated post-training (no rec., light blue), and the non-predictive encoding model (orange). Black diamond shows the means; each point is a neuron or model unit, and the violins show the distributions. Middle: Violin plots showing the corresponding distributions of the coefficient of variation of the interspike intervals, CV(ISI), of the neurons and model units to the natural images. Right: Cross-correlograms of the model units and salamander RGCs. **d** Same as (**c**) but with the statistics instead calculated using the responses to natural movies and with the cross-correlogram calculated from the mouse RGCs. The salamander data were obtained from ref. ^37^, the macaque image and movie data were obtained from ref. ^38^, and the marmoset and mouse movie data were obtained from ref. ^39^. A subset of model units was randomly selected for plots (**a**, **b**) to match the number of neurons.

First, we checked that our model could produce spike trains similar to the retina. We compared the spiking activity of the model units to RGC activity across different animal species in response to natural images and movies. Here, we used publicly available retinal recordings in macaques, marmosets, mice, and salamanders^37–39^. We found that our model units exhibit spiking activity similar to that of the RGCs across each of these species, with both showing that the retina qualitatively exhibits more sparse and regular firing activity in response to natural images^37^ (Fig. 2a) compared to natural movies^39^ (Fig. 2b).

We quantified the spiking responses to the natural images and movies across the different animal species and the non-predictive encoding and prediction models. We used the image dataset from the salamander recordings^37^ to quantify the model’s response statistics to natural images, and a held-out test set from our training movies to calculate the model’s response statistics to natural movies. We measured the spike response sparsity and variability by respectively calculating the mean firing rate and the mean coefficient of variation of the interspike interval CV(ISI). The CV(ISI) measures the variability in a spike train, where CV(ISI) < 1 corresponds to a more regular firing pattern than a Poisson process and CV(ISI) > 1 a less regular one^28^ (see “Methods”). Both the mean firing rate and CV(ISI) are lower across the animal species in response to the natural images (Fig. 2c) than to the natural movies (Fig. 2d). Similarly, we found the prediction model units to exhibit a lower firing rate (images 8.3 Hz vs movies 17 Hz; *p* = 6.0 × 10^−14^, one-sided Mann–Whitney *U* test) and CV(ISI) (images 0.39 vs movies 1.9; *p* = 2.4 × 10^−166^, one-sided Mann–Whitney *U* test) to the images compared to the movies.

The non-predictive encoding model exhibited similar spike statistics to the prediction model, albeit slightly less well aligned to the biology with respect to the CV(ISI). Furthermore, the firing rates and spike variability remained intact when we ablated the recurrent connections from the prediction model. This is consistent with the recurrent connections contributing significantly less (5.6% of total) input current compared to the feedforward connections in the trained prediction model (Supplementary Fig. S2).

We measured the correlation between the spike trains of different units by calculating the spike train cross-correlogram, which assesses the correlation between randomly selected pairs of units at different time lags. For pairs of real RGCs, the peak correlation is at time lag zero for the image (Fig. 2c) and movie datasets (Fig. 2d). A peak at zero was also seen for the prediction and non-predictive encoding models, with the height of the peak correlation for the prediction model being closer to that of the biology in both salamanders and mice. The spike train correlations decayed symmetrically for increasing and decreasing lag for both models, as with the biology. However, for the movie dataset, both the prediction and non-predictive encoding model units tended to be more correlated for non-zero lags compared to mouse RGCs. These elevated correlations persisted when we ablated the recurrent connections in the prediction model (although the peak correlation was slightly decreased).

### Emergence of major retina-like cells

After training for temporal prediction, the model units, which before training were undifferentiated, appeared to separate into different functional types. We analyzed the functional types of the model units, as one would categorize the functional types of RGCs^37^, by mapping the spatiotemporal RFs of the model units using a spike-triggered average to white noise. We fitted a 2D Gaussian function to each unit’s spatial RF and projected the spatial RF size and dimensionality-reduced RF temporal profile onto a 2D scatter plot. We then separated the units into functional groups using k-means clustering (see “Methods”).

**Fig. 3:**
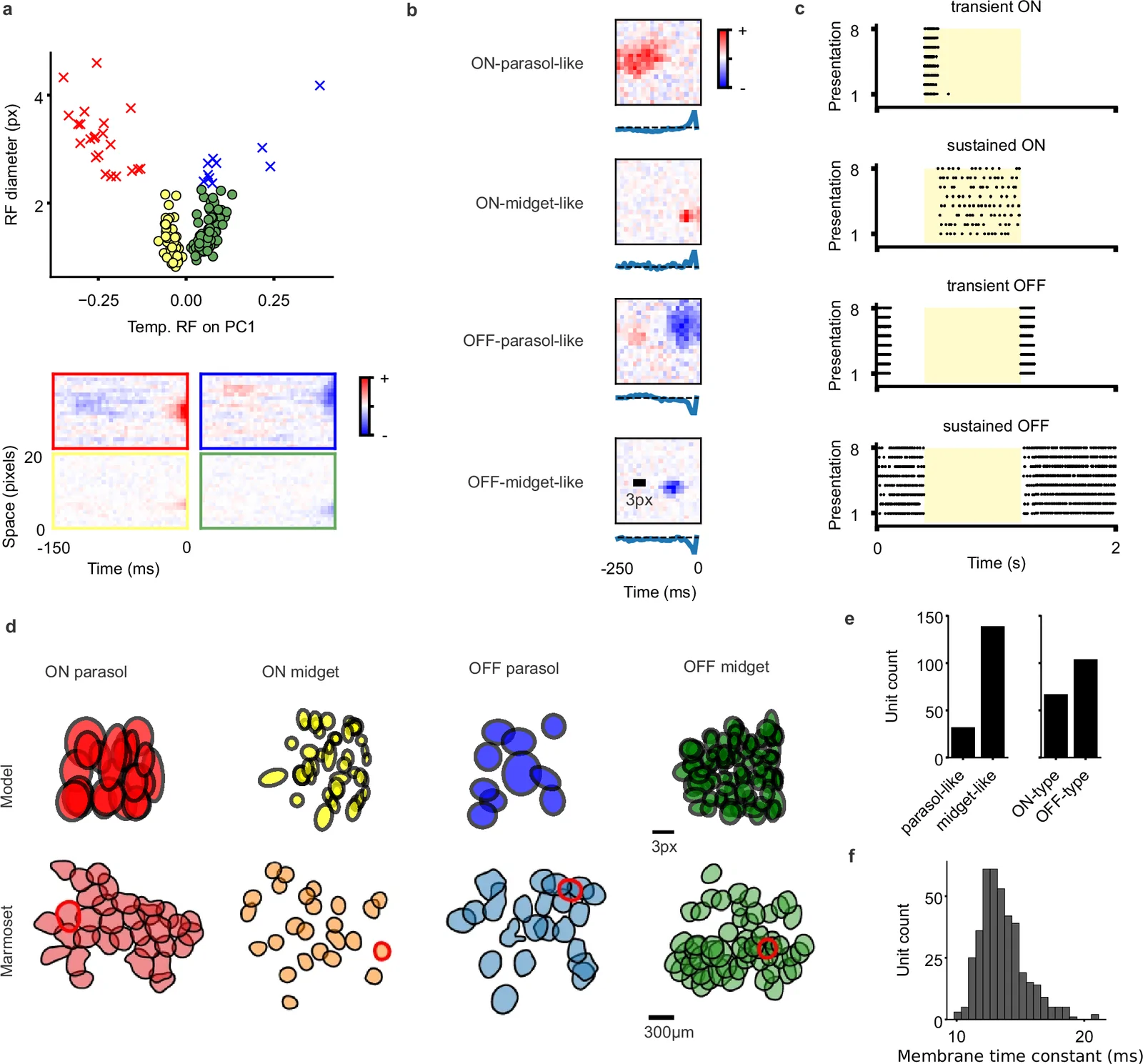
Retina-like RFs and their mosaic organization. **a** Scatter plot of prediction model unit RF diameter (obtained from a 2D Gaussian fit) vs the first principal component (PC1) of the dimensionality-reduced RF temporal profile, with the spatiotemporal RFs obtained from a spike-triggered average to white noise. Separate colors and shapes denote distinct functional groups (obtained using k-means clustering), with red and blue crosses denoting ON-type and OFF-type parasol-like units, respectively, and yellow and green circles denoting ON-type and OFF-type midget-like units, respectively. Example 2D spatiotemporal RFs from each group are plotted below: left are ON-type units, right are OFF-type units, top are parasol-like units, and bottom are midget-like units. **b** An example spatial RF from each group, with corresponding temporal profile plotted below. Red and blue colors denote positive and negative weights, respectively. **c** Light-flash responses of the model units depicted in (**b**) (yellow rectangle denotes the duration of the flash of light). **d** RF mosaics of the different model unit types (top) with the corresponding RF mosaics of the different marmoset RGC types^39^ (bottom). **e** Left: number of model units that are parasol- and midget-like. Right: number of units that are ON- and OFF-type. **f** Membrane time constant distribution over all model units. The marmoset RF mosaics in (**d**) are adapted from ref. ^39^, and used under CC BY 4.0.

The prediction model units separated into four functional classes (Fig. 3a), each resembling one of the major cell types found in the primate retina, which together constitute over 95% of the RGCs in the central retina^40^: ON parasol and midget cells and their OFF type counterparts^40,41^ (Fig. 3b, see Supplementary Fig. S3 for more RFs). As in the human retina, the parasol-like units had a larger spatial structure and a more biphasic temporal profile than the midget-like units^42,43^.

We also found the model parasol- and midget-like units to behave similarly to their biological counterparts in the primate retina in response to flashes of light (Fig. 3c): the ON and OFF parasol-like units responded with a transient burst of spikes to changes in light intensity, and the ON and OFF midget-like units responded with a sustained train of spikes (with the ON-type cells responding to the light onset, and the OFF-type cells responding to the light offset)^44,45^. We observed the response correlations between units of the same functional class to be larger than the correlation between units of different classes (Supplementary Fig. S4). The ON parasol-like units exhibited the largest within-class correlations, as has similarly been found in marmosets^39^.

As reported in the retina across various mammalian species^46–48^, the RFs of the different unit types in the model tiled visual space, forming mosaic-like organizations (Fig. 3d). The degree of RF overlap between OFF-type midget-like units more closely resembles the spatial arrangement of RGC RFs^49^ than is the case for the other model unit types, which were either more overlapping or more widely spaced (Supplementary Fig. S5). As the clusters for the different unit classes are quite distinct (Fig. 3a), this overlap is unlikely to be due to poor cell-type assignment.

We found fewer parasol- than midget-like units, and fewer ON- than OFF-type units within the model (Fig. 3e). This aligns with experimental reports of midget cells being more plentiful than parasol cells in the human retina^40,50^, and OFF-type cells occurring more frequently than ON-type cells in guinea pigs^51^ and humans^43^. Lastly, the model units exhibit a heterogeneous membrane time constant distribution on the scale of tens of milliseconds, which has similarly been reported for cat RGCs^52^ (Fig. 3f).

The RGC-like RF tuning properties were less evident in the non-predictive encoding model, in which we found parasol-cell-like RFs were missing (Supplementary Fig. S6). We also found that the metabolic constraint is important in obtaining the results of the prediction model. Excluding the constraint causes the model’s RFs to become broad and indistinct, losing their uni-peaked oval-like shape, which is hence less similar to the recorded RFs (Supplementary Fig. S7).

Note that the spatial RFs were taken at the time point of each spatiotemporal RF with the largest overall power. At this time point, the surround is weak or absent (Fig. 3b); however, tens of milliseconds further into the past of the spatiotemporal RFs, the model units often have a clear surround (Supplementary Fig. S8). Studies in guinea pigs^53^ and primates^54^ have shown that the surround also tends to lag the center, suggesting that this biological phenomenon may not just be a mechanistic consequence of the circuitry, but may actually serve a functional purpose for improving temporal prediction.

### Differential and anticipatory motion selectivity

**Fig. 4:**
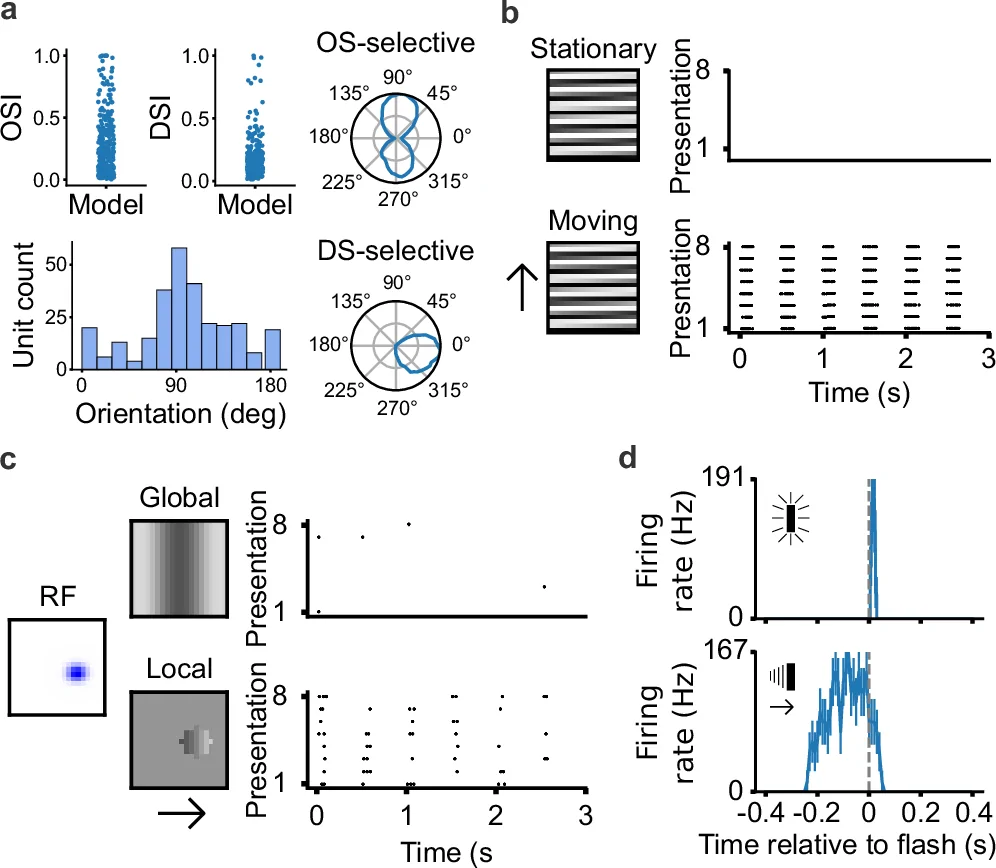
Motion tuning. **a** Top left: prediction model unit orientation- and direction-selectivity distributions (values closer to unity denote stronger tuning). Bottom left: distribution of orientation tuning for the model units. Right: sample tuning curves of an orientation-selective model unit and a direction-selective model unit. **b** Spike raster of a model motion-sensitive unit, with no response to a static grating of high spatial frequency (top), and a response to the grating moving upwards (bottom). **c** Spike raster of an object-motion-sensitive model unit, with no clear response to the grating moving over the entire visual space (top), and a stronger response to the same spatially localized grating moving within the model unit’s RF (bottom). **d** Top: responses of a single model unit to a briefly flashed bar within their RFs. Bottom: Anticipation-like responses in the same model unit to a rightward moving bar (0.125 pixels/frame) before the bar crosses the RFs. The flashed and moving bars were aligned at time zero, and the blue line shows the mean firing rate, and error bars denote the s.e. over repeated stimulus presentations.

We quantified the tuning characteristics of the model units and found that they exhibited several well-known motion-tuning characteristics of RGCs. Tuning to the orientation or direction of moving objects, with a preference for motion along the cardinal axes, has been reported in a range of vertebrate species^55–62^. In keeping with these observations, we found that some model units were selective for orientation (16% of units with OSI > 0.5) and direction (4.5% of units with DSI > 0.5), with a preference for motion along the horizontal and vertical axes (Fig. 4a). Ablating the recurrent connections slightly increased the proportion of orientation tuned units to 17% but decreased direction tuned units to 4.2%. A well-known class of RGCs, the so-called Y-type cells (~3% of ganglion cells in cats^63^), are tuned to the general motion of textures of high spatial frequency, but not their static presentation^22,64–66^. Similar to these neurons, we found units in the model (9.1% of units) that did not respond to a static grating of high spatial frequency (at each unit’s preferred orientation), but fired as soon as the grating moved (Fig. 4b). Ablating the recurrent connections reduced the proportion of units with this property to 8.4%.

Some vertebrate RGCs are object-motion-sensitive (OMS) cells that exhibit differential motion selectivity. This is where a neuron remains silent when an image (or grating) moves across the retina yet fires if the motion in the RF center differs from that in the wider surround^27,57,67,68^, such as when the center motion varies independently from that of the surround or when the surround is masked. We also found such OMS tuning in some of the model units for both masked (Fig. 4c) and differential surrounds (Supplementary Fig. S9). We measured the OMS tuning of the population for the differential surround stimulus: the mean object-motion sensitivity index^57^ was positive at 0.11, indicating net sensitivity to differential motion, and this was somewhat reduced to 0.10 when the recurrency was ablated (*p* = 3.9 × 10^−3^, two-sided Wilcoxon signed-rank test). This is consistent with results suggesting a role for amacrine cells in this phenomenon^27,57,67,68^.

OFF-type RGCs in salamanders and rabbits have been shown to produce an anticipation-like response to moving objects (such as a moving bar), where the neuron fires even before the object crosses the neuron’s RF, whereas there is a relatively delayed response to simply flashing the bar onto the RF^26^. The spatial profile of the population response of such cells along the axis of motion lies ahead of the population response of a coincident flashed bar. Indeed, the peak of the population response also tends to be equal to or ahead of the leading edge of the moving bar by an approximately constant distance, except at high speeds. Similarly, we found that certain units in the prediction model produced an anticipation-like response to a moving bar (Fig. 4d). Furthermore, we found similar population response properties in the OFF-type parasol-like units of the model (Supplementary Fig. S10), although given the small number of such units, these results are tentative. The peak of the anticipation-like response of the model unit population depended to a degree on the recurrent connections, since ablating them resulted in it leading the moving bar by a shorter distance (Supplementary Fig. S10). This suggests that gap junctions and/or amacrine cells between RGCs may play a role in motion anticipation.

### Emergence of a relative spike latency code and stimulus omission responses

A contentious and open question in neuroscience is how neurons transmit information using their spikes^69^. This code has historically been cast as two extremes, where neurons either communicate using the number of spikes evoked (i.e., a rate code) or the timing of spikes (i.e., a temporal code). These strategies are not necessarily mutually exclusive^70^, and different neurons likely employ different coding strategies depending on their location within the nervous system. In the retina, there is strong evidence that certain RGCs employ a temporal-like code, encoding the spatial structure of a new visual scene using the relative timing of the first spikes. Reconstructing natural images using the differential spike latencies of fast OFF RGCs in the isolated salamander retina, rather than their spike counts, qualitatively results in a clearer image representation^23^.

**Fig. 5:**
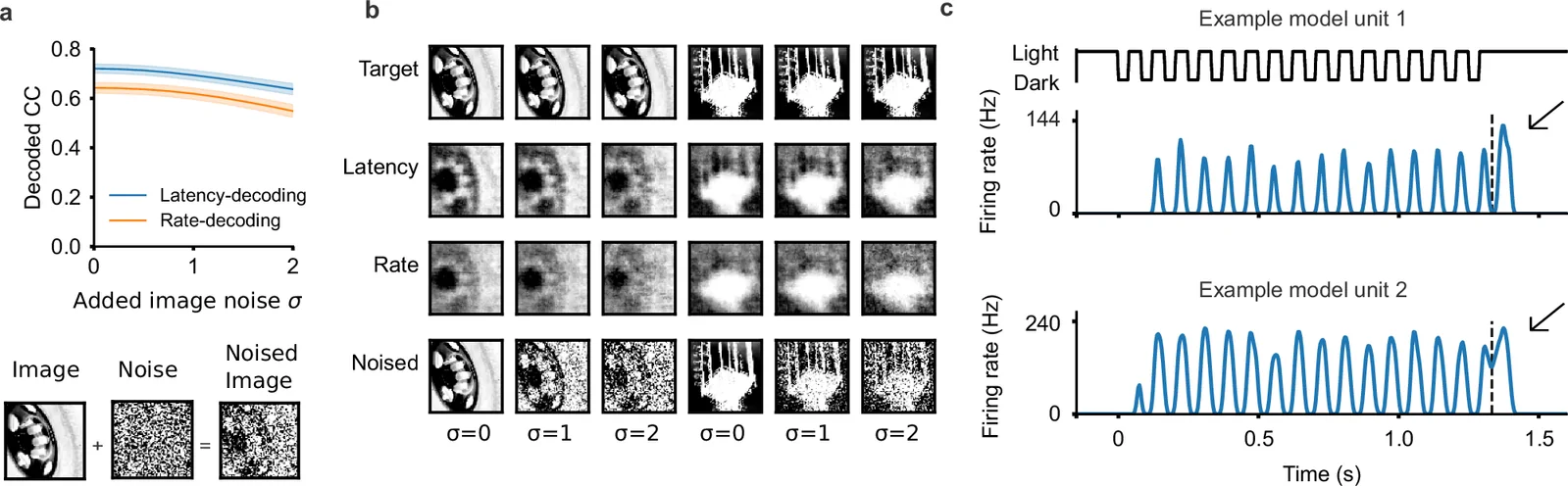
Latency and stimulus-omission responses. **a** Top: mean decoding accuracy (Pearson correlation coefficient) in reconstructing natural images from noise-corrupted inputs using a linear transformation of the population model activity with a latency code (blue line) and a rate code (orange line). The shaded area is the standard error of the mean over images. Bottom: illustration of noise corrupting the input images. **b** Example image reconstructions from (**a**). Rows from top to bottom are: target image, latency-decoding estimate, rate-decoding estimate, noised-image input. **c** Responses of two example model units (blue) during a sequence of 16 40-ms dark-flashes (brief luminance reductions) presented at 12 Hz (top). The vertical dashed lines in the response plots mark the time when the next dark-flash would have started, with the arrow indicating the omitted-stimulus response. Omission stimulus responses were also present at different dark-flash rates, as well as when dark-flashes were omitted in the middle of the sequence (Supplementary Fig. S12).

We examined these coding strategies in our model and similarly found that a rate-based reconstruction was qualitatively more blurry and noisy compared to a latency-based reconstruction (Fig. 5a, b, Supplementary Fig. S11). A differential spike latency code can transmit new visual information using very few spikes, which is both energy efficient and fast. This is particularly important because gaze tends to remain fixed for only a fraction of a second, before a rapid saccadic eye movement to a new fixation point causes a different span of the visual scene to be projected upon the retina^71,72^.

Most of our understanding of the retina’s neural code is at a single-cell level based on responses to artificial stimuli, like drifting gratings^22,73^. It is less well understood how natural stimuli are encoded by the population activity. Additionally, it remains to be understood how the different coding strategies compare to each other in the presence of varying levels of noise. This is important because the retina likely employs a noise-robust coding strategy, given the ubiquity of noise in the retina and the visual environment^74^. To explore this, we tested how well we could reconstruct natural images by inputting their noise-corrupted counterparts into the model and linearly decoding the images from the resulting rate and latency codes of the population model spike activity. We found that the latency-decoding strategy more robustly decoded the noise-corrupted natural images than the rate-decoding strategy (Fig. 5a), qualitatively producing clearer image reconstructions, even under high levels of noise corruption (Fig. 5b).

Certain RGCs in the salamander and mouse retina have been reported to produce an omitted-stimulus response (OSR)^24,25^. Such neural responses are generally thought to encode prediction or prediction errors^75^. RGCs exhibiting OSRs fire in response to a violation in a periodic temporal pattern, such as an omitted dark-flash (brief luminance reduction) within a steady sequence of dark flashes^24,25^. We found that some of the model units (16%) produced an OSR to a violation in a periodic sequence of dark-flashing at a rate of 12 Hz (see “Methods”). These units’ responses persisted throughout the dark-flash sequence, but also responded at the end of the sequence, around the expected time of the response to the omitted dark-flash (Fig. 5c). We also found OSRs to be present for slower (8 Hz) and faster (16 Hz and 20 Hz) dark-flash sequences, as well as when dark-flashes were omitted in the middle of the sequence (Supplementary Fig. S12). Substantial OSR responses were only present in the model optimized for temporal prediction and not in the model optimized for non-predictive encoding.

For the temporal prediction model, across the population of units, the latency of the OSR peak relative to the peak of the response to the last dark-flash increased with respect to the period of the dark-flash sequence (Supplementary Fig. S13), as has similarly been reported in salamander RGCs^24^. Furthermore, when we ablated the recurrent connections, the OSRs were still present, and for the population of units, the dependence of the OSR timing on the period was little altered except for the low-frequency sequences (at 8 Hz). In contrast, this OSR timing dependence on period seen in the population was greatly weakened when it was examined for the feedforward input current alone. This suggests a role for the integrate-and-fire mechanism in the generation of OSRs.

Inspection of the input weights of the OSR units revealed them to often have a biphasic temporal profile—with the net weighting changing from positive to negative as the weights extend into the past, or with decreasing negativity and then a slight increase (Supplementary Fig. S14). This biphasic tendency was also reflected in their RFs (Supplementary Fig. S15). We observed these biphasic properties much less in the non-predictive encoding model units and in prediction model units that did not produce OSRs. We confirmed these differences between OSR and non-OSR units with a quantification of the biphasic character of the input weights (Supplementary Fig. S16), although there was considerable overlap between OSR and non-OSR units.

Together, this suggests that OSRs in the model are largely a consequence of the biphasically sculpted input current interacting with the spiking mechanism, with a lesser role for the recurrent connections. This is consistent with experimental results in salamanders, suggesting that inhibition from amacrine cells is not needed for OSRs at 12 Hz, but ON bipolar cells are^25^. The importance for OSR timing of the spiking mechanism and of the recurrent connections at low frequencies are experimental predictions of the model.

### The temporal prediction model captures retinal responses in different species

**Fig. 6:**
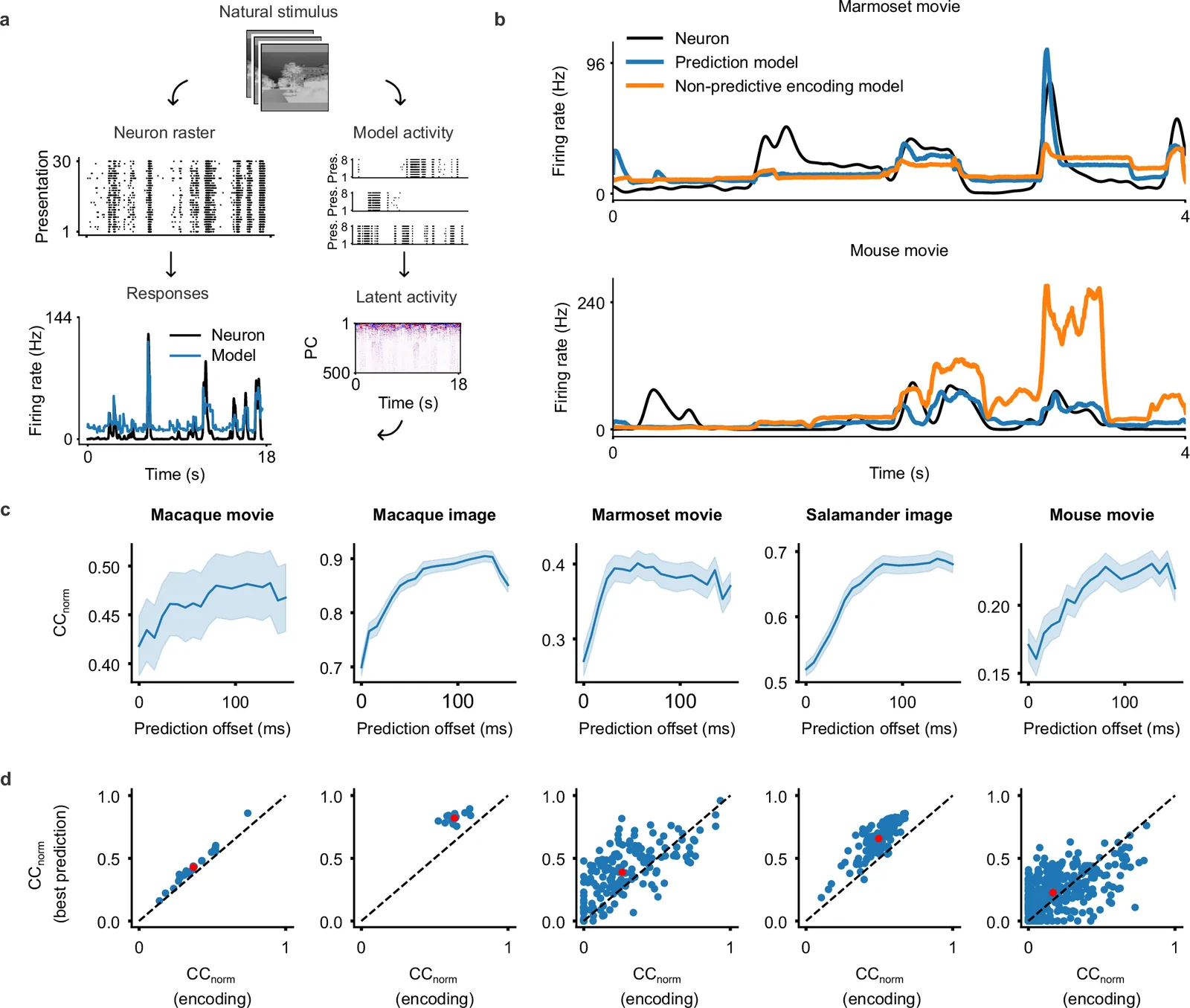
Predicting RGC responses to natural images and movies using the spiking models. **a** Each retinal model (trained to encode the present or predict different offsets into the future) was fitted to predict the neural responses across multiple natural stimuli in different retinal datasets. Model unit activity was obtained for each stimulus set used in these studies and downsampled into a latent activity space using principal component analyses, from which the neural responses were predicted using a regularized linear-non-linear readout (see “Methods”). All results report the normalized correlation coefficient between the predicted and the target neural responses, computed on a held-out test set. **b** Sample neural response (black line) of a single neuron in the marmoset retina (top) and mouse retina (bottom) to natural movie stimuli. Predicted responses of the prediction model (blue line) and non-predictive encoding model (orange line) are superimposed. **c** Neural prediction scores across the different retinal datasets and retinal models, as a function of the prediction offset. The blue line is the mean score and the blue shaded area is the standard error of the mean over neurons. **d** Neuron prediction scores of the best temporal prediction model (*y*-axis, optimal offset into the future) against the non-predictive encoding model (*x*-axis, 0 ms into the future) across the different datasets (with the red dot plotting the centroid).

We assessed whether optimizing the retinal model to encode the present or the future better predicts the activity of RGCs across different vertebrate species in response to natural images and movies. We used five publicly available datasets of RGC recordings in macaque, marmoset, mouse, and salamander^37–39^. For every dataset and retinal model, we fitted a linear-non-linear readout from the retinal model to predict the RGC responses and quantified the performance of each neuron’s fit as the normalized correlation coefficient (*CC*_*norm*_) between the predicted and the target neuron response on a held-out test set^76^. As is commonly done^77^, we performed the fits from a dimensionality-reduced latent representation of the model’s hidden-unit activity in response to the different dataset inputs (Fig. 6a; see “Methods”).

Across all animal species and stimulus types, we found the retinal model optimized for prediction to more accurately capture the neural responses than the non-predictive encoding model. Qualitatively, this advantage appeared to be particularly present at the peaks in the firing rate profiles (Fig. 6b). Notably, the mean *CC*_*norm*_ steadily increased across all datasets for increasing model prediction offset (i.e., the further the retinal model predicted into the future, the better the neural fits), until a maximum score was reached, after which prediction performance declined (Fig. 6c). Across the datasets, the maximum *CC*_*norm*_ was obtained using the retinal model optimized to predict 56–128 ms into the future. Lastly, the higher *CC*_*norm*_ for prediction was driven by the majority of neurons, rather than by a small subset (Fig. 6d). Hence, it can be seen that the value of *T*_off_ at 128 ms used in previous figures is justified as a value that gives high response prediction across multiple species.

## Discussion

We set out to uncover the principles underlying the sensory transformation performed by the retina, from the incoming light to the outgoing spikes conducted by the optic nerve. Does the retina efficiently compress incoming input^4–8^ or efficiently extract sensory features predictive of the future^11–15^? To examine this question, we implemented a biologically detailed spiking model of the retina, which we optimized for either efficient compression or efficient prediction using natural movie stimuli. The model optimized for prediction exhibited various retina-like phenomena, not all of which were captured by the model optimized for efficient compression. Notably, the temporal prediction model more accurately predicted RGC responses to both natural images and movies that have been recorded in a range of vertebrate species. These findings suggest an evolutionarily conserved role for the retina in informing the brain about temporally predictive features in the visual world.

Normative approaches enable the underlying principles behind the sensory transformations of a biological circuit to be identified^19–21^. Prior studies developed models of the retina using non-spiking units optimized for efficient coding by maximizing the mutual information between the sensory input and model unit firing rates^5–8^. These models accounted for the retina’s spatial RFs^4–8^, their temporal profiles^4,6^, and their mosaic-like organization^5–8^.

We asked whether normative principles such as compression or prediction explain RGC properties when spikes are used for communication. To this end, we developed a retinal model with units that output individual spikes, providing greater biological realism over prior work that used firing rate outputs^4–8^. Our model exhibited retina-like spike statistics to natural images and movies, as quantified using firing rates and measures of spike variability, and more accurately encoded sensory input using relative spike latencies than firing rates, as has also been reported in the retina^23^. Furthermore, unlike other models^4–8^, our model includes recurrent connectivity to capture the interplay between RGCs. We trained the model on natural movie recordings, whereas previous work has generally omitted temporal dynamics by training on natural images^5,7,8^ (although see ref. ^6^). Finally, our model was trained to minimize a detailed metabolic-like loss, approximating synaptic energy requirements, in contrast to previous studies, which have simply constrained model firing rates^4–8^.

In addition to capturing the spatial RFs of RGCs, their temporal dynamics, and, to some degree, their mosaic-like spatial organization, the prediction model exhibited several other properties that have been described in a range of vertebrate species, and which have not been accounted for by other normative studies of the retina. This includes finding units tuned to the direction and orientation of moving gratings^78^; units that exclusively fire to high-spatial-frequency gratings only if they move, like Y-type cells^22^; units tuned for the differential motion of objects versus their background^27^; units that appear to anticipate moving objects^26^; and units that respond to stimulus omission within a temporal sequence of dark flashes^24,25^. Although stimulus omission and anticipation responses also emerge in non-spiking encoding models fitted to retinal responses^79,80^, these are not normative approaches aimed at investigating underlying principles like compression or prediction.

The temporal prediction model most accurately predicted neural responses to natural images and movies when it was trained to predict future inputs on a timescale of ~50–100 ms. It is interesting to speculate on why this particular timescale might be important. One reason could be that an animal needs to know the likely state of the world at the time of future immediate action, for example to catch prey or avoid predators. This would be a fraction of a second into the future, whereas shorter or longer timescales may be less effective for this role. This timescale can account for transduction delays, which are on the order of tens of milliseconds at the photoreceptor^81,82^, as well as neural transmission delays and movement delays for very fast motor responses^83^.

This timescale is in some respects consistent with a prior study^11^ showing that, for naturalistic stimuli, the retina carries predictive information about its own activity out to hundreds of milliseconds ahead. That study did not involve a normative model and used different measures: the mutual information in the retinal response about the full future of simple one-dimensional Brownian-motion stimuli, and information about future responses in the current retinal responses for naturalistic stimuli. Another study^19^ maximized the predictive information of a non-spiking linear model about naturalistic visual input at future time-points. These authors observed units with RFs that resembled the simple cells of the primary visual cortex, but without direction selectivity. In contrast, our model units had RGC-like RFs that captured diverse characteristics displayed by these neurons, including direction selectivity. These differences likely arise because the temporal prediction model is non-linear, uses strong synaptic activity constraints, and was trained on movies rather than shifted image-patches.

It is striking that a single-layer network, with many simplifications compared to the real retina, reproduces so many of its features. This may partly be because the simplifications included in our model, such as linear photoreceptor/bipolar layers and ignoring the details of amacrine cells and gap junctions, still appropriately capture many of the crucial features for the response properties examined. However, this finding also implies that the response properties of the network (and the retina) are more a consequence of the objective and stimulus statistics than the details of the architecture, and that different architectures may achieve comparable functional results when optimized for temporal prediction. Indeed, this is why we used a relatively generic recurrent architecture rather than incorporating species-specific biological detail. Our aim was to determine whether diverse retinal features could arise as a consequence of a temporal prediction objective and natural stimulus statistics, rather than be contingent on particular biological structures that are hard-coded into the model.

This is not to suggest, however, that adding more biological detail would not extend the explanatory power of our spiking temporal prediction model. Indeed, while the inclusion of spikes and recurrent connections increased realism over prior models, there remains scope to incorporate more of the retina’s biological complexities and processes. For instance, RGCs in different vertebrate species adapt to stimulus contrast^29,84,85^, which could potentially be modeled using adaptive LIF neurons^28,86^. We could also include constraints on the recurrent connectivity by explicitly modeling the amacrine cells^22,31^ and gap junctions between the spiking units to better emulate the coupling between RGCs^31,32^, including non-linear effects of amacrine cells^87^.

We modeled the photoreceptors and bipolar cells as a linear transformation, given reports of their near-linear integration properties^29^. However, bipolar cells can exhibit some non-linear integration^88^; thus, modeling them with an additional layer of non-linear graded units may, for example, enable better tiling by the mosaics. Furthermore, photoreceptors show non-linear adaptation, which may mechanistically underlie RGC responses to luminance changes in natural stimuli^89^. Endowing the photoreceptor and bipolar units with the capacity to develop non-linear adaptation would allow investigation of whether luminance adaptation is also a functional consequence of temporal prediction. Furthermore, it may be worth applying to the stimuli some low-pass temporal integration, reflecting the relative slowness of phototransduction, alongside substantially limiting the span of input latencies. This may address the difference in the temporal RF profile of the model units compared to RGCs, and also potentially help to produce stronger RF surrounds^39^ and a greater role for recurrent connections.

Instead of training our model on randomly sampled normalized grayscale movie patches, we could use non-negative pixel values to capture the non-negative nature of light intensity, or use color movies to examine the processing of color in the primate retina^90^. Future extensions could additionally develop and train the model on the entire visual scene rather than patches. This could be a convolutional model^91^, or better yet, a model composed of overlapping unique patches, each operating at a different spatial location within the visual frame. A patch model may capture how the tuning properties of RGCs differ across the retina, such as in their direction selectivity^92^. However, training such large spiking models remains a challenging problem due to the speed and memory constraints of digital computers (although see refs. ^86,93^).

Another extension would be to further explore the effect of noise in the model, particularly with regard to RGC RF surrounds. RGCs have typically been characterized as having a center-surround RF organization^22^, although, as is the case with our prediction model units, some studies in primates have reported relatively weak surrounds^43,94^. Interestingly, the RFs of the prediction model often show lagged surrounds, in keeping with RGC recordings in primates^54^ and guinea pigs^53^. It is possible that learning the noise sources in the model, instead of hard-coding them, would result in RFs with stronger surrounds^7^. Alternatively, differences in surrounds may result from the way RFs are mapped, where spike-triggered averages—as employed in our study—result in RFs with less defined surrounds^95^.

We did not focus on one specific species, but rather searched for general retinal characteristics that are relatively common across vertebrate species. For example, we determined whether model units exhibited ON- and OFF-sustained and ON- and OFF-transient responses that are common across many species, such as the cat X and Y cells^96^, primate (macaque, marmoset, human) midget and parasol cells^97^, and mouse alpha cells^98^. It would be interesting in future studies to tailor the model to the particular stimulus statistics and constraints of individual species. This might better capture, for example, mouse retinal responses to natural stimuli, which the models presented here predicted somewhat worse than for the other species we examined. It might also explain species-specific distinctions between RGC types, for example in the extent to which they exhibit direction selectivity^99^.

Finally, although each cell type produced by our model approximately spanned visual space, this mosaic tiling is imperfect with noticeable gaps and overlaps that are less apparent in the biology. Such tiling has been better captured by some efficient coding models^5–8^. However, none of these models had the additional constraint of being spiking, and beyond our non-predictive encoding model, it remains to be seen how different instantiations of efficient coding/compression with spiking would perform. Nevertheless, that we do see tiling to some degree despite our prediction model objective focusing on temporal rather than spatial aspects is notable. Training the temporal prediction model with more units or with additional efficiency constraints, such as on physical space, may enable more even and well-spaced tiling. Indeed, it may be worth exploring models with an objective combining both efficient coding of current input with temporal prediction of future input.

The spiking temporal prediction model might have clinical implications in the development of retinal prosthetics. Current prosthetics use standard computer vision algorithms to translate camera signals into a stimulatory code^100,101^, which fall short of delivering a normal visual experience^102^. We anticipate that the sensory transformation implemented in our model could aid in delivering a more RGC-like stimulatory signal.

In conclusion, consistent with the capacity of temporal prediction models to explain diverse properties of visual^15,17,103,104^ and auditory cortex^17^, this study adds to the evidence that sensory systems are geared towards prediction of future input. Optimization of sensory systems for temporal prediction enables estimation of the world in the future when action will occur, compensating for transduction, processing, and motor delays, while also eliminating irrelevant information and extracting underlying variables^16,17^. Thus, temporal prediction may be a governing principle of sensory systems.

## Methods

### Spiking retinal model

#### Training data

We recorded a natural visual stimulus dataset for model training. Various types of scenes were obtained, categorized as flow (camera moving through space), pan (camera panning through space), still (fixed camera recording a still scene), and still-moving (fixed camera recording an active scene). A total of 39 clips of 9.6-s duration were recorded at a frame rate of 120 Hz and a spatial extent of 720 × 1280 pixels. The dataset was divided into a training and test set, both containing a balanced number of the different recording types (training set: 12 flow, 8 pan, 3 still, and 8 still-moving clips; test set: 3 flow, 2 pan, 1 still, and 2 still-moving clips).

All clips were pre-processed by converting the RGB channels to grayscale (using a weighted average of the color channels of R:G:B = 30:59:11^37^). They were then bilinearly downsampled to spatial dimensions of 144 × 256 and centrally cropped to 140 × 240 pixels. The clips were then normalized by subtracting the mean and dividing by the standard deviation of the training set pixels. Finally, the temporal resolution was artificially increased to 240 Hz by duplicating every frame. The model was trained on temporally non-overlapping snippets that were 20 × 20 pixel patches of 320 ms duration (i.e., 77 frames), with each snippet being from a randomly sampled spatial position. Hence, each snippet had *T* = 77 time steps, *Q* = 20 pixels width and *H* = 20 pixels height. We artificially doubled the training data using data augmentation by flipping each snippet around the vertical axis^105^.

#### Spiking neurons

We modeled the RGCs in the retina using a single hidden layer of *N* = 400 recurrently connected spiking units, providing a flexible model for optimization to an objective. Unlike simpler linear-non-linear models^15,17^, which lack spikes, and generalized linear models^30^, which use a Poisson-process spike generator, we simulate our units using the leaky-integrate-and-fire model. As in real neurons, our model includes an evolving membrane potential and a spiking output with a reset mechanism. The model comprised the following series of computational steps:

Step 1, photoreceptor noise: to emulate the noisiness of the photoreceptors^33^, we added independent and identically distributed (i.i.d) Gaussian noise ${\epsilon }^{{{\rm{photo}}}} \sim {{\mathcal{N}}}(0,0.01)$ to each pixel at each time step *t* for each input movie snippet. The frame of a snippet at time *t* is ${{\bf{U}}}[t]\in {{\mathbb{R}}}^{Q\times H}$. This produced noisy input movie snippets, with the frame of a noisy snippet at time *t* being $\tilde{{{\bf{U}}}}[t]\in {{\mathbb{R}}}^{Q\times H}$. The elements of **U**[*t*] and $\tilde{{{\bf{U}}}}[t]$ are denoted by *u*_*xy*_[*t*] and ${\tilde{u}}_{xy}[t]$, respectively, for horizontal position *x*, vertical position *y*, and time step *t*.

Step 2, input current: the input current ${I}_{i}[t]\in {\mathbb{R}}$ to unit *i* at time step *t* is obtained from the noisy input movie snippet, the model output spikes ${{\bf{S}}}[t-1]\in {{\mathbb{R}}}^{N}$ and a bias term ${b}_{i}\in {\mathbb{R}}$.

1 $${I}_{i}[t]={b}_{i}+\underbrace{{\sum }_{\tau=1}^{{T}_{E}}{\sum }_{x=1}^{Q}{\sum }_{y=1}^{H}{w}_{i\tau xy}{\tilde{u}}_{xy}[t-\tau+1]}_{{{\rm{Feedforward}}}\,{{\rm{current}}}}+\underbrace{{\sum }_{j=1,j\ne i}^{N}{w}_{ij}^{{{\rm{rec}}}}{S}_{j}[t-1]}_{{{\rm{Recurrent}}}\,{{\rm{current}}}}$$

The feedforward connectivity ${{\bf{W}}}\in {{\mathbb{R}}}^{N\times {T}_{E}\times Q\times H}$ linearly maps the noisy-input movie stimulus to a feedforward current contribution and captures the transformation of the pre-RGC cells. We set the span of latencies to *T*_*E*_ = 30 time steps (125 ms) in the feedforward connectivity, consistent with the potentially wide integration times of the photoreceptors^82^. The recurrent connectivity ${{{\bf{W}}}}^{{{\rm{rec}}}}\in {{\mathbb{R}}}^{N\times N}$ (autapse connections were excluded) maps the output spikes into a recurrent current contribution to capture the ganglion-to-ganglion cell and ganglion-to-amacrine-to-ganglion cell interactions. The elements of **W** and **W**^rec^ are given by *w*_*iτxy*_ and ${w}_{ij}^{{{\rm{rec}}}}$, respectively.

The connections of the model units represent a diverse collection of pathways. The feedforward model connections reflect the effect of photoreceptors on ganglion cells via bipolar cells, including the contributions of horizontal cells and those of amacrine cells that are not mediated through RGCs. The excitatory recurrent model connections approximate the excitatory influence of RGCs on other RGCs via gap junctions^32^ and via the rare glutamatergic amacrine cells^106^. Finally, the inhibitory recurrent model connections approximate the inhibitory effect of RGCs on other RGCs via amacrine cells, either by the amacrine cells impacting RGC activity directly or acting via bipolar cells, with the RGCs themselves activating the amacrine cells by gap junctions^107^.

Step 3, RGC noise: to emulate RGC noise^34^, we multiplicatively perturbed the input current *I*_*i*_[*t*] of unit *i* at every time step *t* using i.i.d. Gaussian noise ${\epsilon }^{{{\rm{gang}}}} \sim {{\mathcal{N}}}(0,0.6)$ to give the noisy input current ${\tilde{I}}_{i}[t]$. This approximates noise sources, such as stochastic vesicle release, and while this noise model could be made more biologically accurate^108^, it suffices for our purposes. We chose this noise source (as well as the photoreceptor noise) such that the model’s spike train variability matched that of the RGCs across repeated stimulus presentations (Supplementary Fig. S1).

2 $${\tilde{I}}_{i}[t]={I}_{i}[t](1+{\epsilon }_{i}^{{{\rm{gang}}}}[t])$$

Step 4, RGC transformation: we modeled all RGC spiking units using the normalized and discretized LIF model^86^. This gives the model membrane potential ${V}_{i}[t]\in {\mathbb{R}}$ of unit *i* at time *t* using the difference equation:

3 $${V}_{i}[t]=\left({\beta }_{i}{V}_{i}[t-1]+(1-{\beta }_{i}){\tilde{I}}_{i}[t]\right)\left(1-{S}_{i}[t-1]\right)$$

The membrane potential decays by a learned factor *β*_*i*_ at every simulation step and resets to zero if a spike occurred in the previous simulation step. A spike is emitted if the membrane potential exceeds the firing threshold (equal to one).

4 $${S}_{i}[t]=\left\{\begin{array}{ll}1\quad &\,{{\mbox{if}}}\,\,{V}_{i}[t] > 1\\ 0\quad &\,{{\mbox{otherwise}}}\,\end{array}\right.$$

Step 5, stimulus estimation: finally, at every simulation step *t*, a span of *T*_*D*_ = 2 steps of proceding spike activity was linearly mapped using projection weights ${{\bf{M}}}\in {{\mathbb{R}}}^{N\times {T}_{D}\times Q\times H}$ to generate an estimation $\hat{{{\bf{U}}}}[t]\in {{\mathbb{R}}}^{Q\times H}$ of the target movie frame (either of the present or the future). This linear mapping is not envisaged as a part of the retinal network; rather, it approximates a posited evolutionarily enforced constraint that causes the network to use representations that can be linearly decoded to provide an estimate of the present or future input^17^. The bias term of this mapping is given by ${b}_{xy}^{{{\rm{proj}}}}$. The elements of $\hat{{{\bf{U}}}}[t]$ and **M** are ${\hat{u}}_{xy}[t]$ and *m*_*iτxy*_, respectively.

5 $${\hat{u}}_{xy}[t]={b}_{xy}^{{{\rm{proj}}}}+{\sum }_{i=1}^{N}{\sum }_{\tau=1}^{{T}_{D}}{m}_{i\tau xy}{S}_{i}[t-\tau+1]$$

#### Bounding membrane time constants

We clipped the *β*_*i*_ value of every spiking unit to be between 0.0001 and 0.9999 to help ensure that the network employed biologically realistic membrane potentials^109^. The membrane time constant in milliseconds for unit *i* was calculated from *β*_*i*_ and the frame rate *f* = 240 Hz by $-1000/(f\ln ({\beta }_{i}))$.

#### Weight initialization

The initial feedforward weights **W**, recurrent weights **W**^rec^ and projection weights **M** were all sampled from an i.i.d. uniform distribution from −*a* to *a*, with $a=\frac{1}{\sqrt{{T}_{E}QH}}$, $a=\frac{0.1}{\sqrt{N}}$ and $a=\frac{1}{\sqrt{N{T}_{D}}}$ for the feedforward, recurrent and projection weights, respectively. All initial membrane time constants were set to 20 ms (i.e., *β*_*i*_ ≈ 0.81). All initial bias terms were set to zero.

#### Loss function

The model was trained, by minimizing loss ${{{\mathcal{L}}}}^{{{\rm{total}}}}$, to generate a single-frame projection of the natural movie stimulus at every simulation step *t* (corresponding to loss ${{{\mathcal{L}}}}^{{{\rm{proj}}}}$) under a metabolic-like constraint (corresponding to loss ${{{\mathcal{L}}}}^{{{\rm{metab}}}}$), weighted by hyperparameter *λ* = 10^−2.5^ (which by qualitative inspection we determined to produce the most retina-like RFs across all models).

6 $${{\mathcal{L}}}^{{\rm{total}}}={{\mathcal{L}}}^{{\rm{proj}}}+\lambda \underbrace{\left(\gamma {{\mathcal{L}}}^{{\rm{graded}}}+(1 - \gamma) {{\mathcal{L}}}^{{\rm{spiking}}} \right)}_{{{\rm{Metabolic}}}\,{{\rm{loss}}}\, {{\mathcal{L}}}^{{\rm{metab}}}}$$

The projection loss ${{{\mathcal{L}}}}^{{{\rm{proj}}}}$ is the mean squared error (MSE) between every pixel in the generated single-frame projection $\hat{{{\bf{U}}}}[t]\in {{\mathbb{R}}}^{Q\times H}$ and the corresponding natural stimulus target frame ${{\bf{U}}}[t+{T}_{{{\rm{off}}}}^{{{\rm{steps}}}}]\in {{\mathbb{R}}}^{Q\times H}$. The offset ${T}_{{{\rm{off}}}}^{{{\rm{steps}}}}$ specifies in time-steps if the target frame is of the present (i.e., ${T}_{{{\rm{off}}}}^{{{\rm{steps}}}}=0$) or of the future (i.e., ${T}_{{{\rm{off}}}}^{{{\rm{steps}}}} > 0$), and is converted to the offset in milliseconds *T*_off_ by ${T}_{{{\rm{off}}}}=1000 \times {T}_{{{\rm{off}}}}^{{{\rm{steps}}}}/f$, where *f* is the frame rate of 240 Hz. The projection loss was averaged over time steps *t*, starting from time step *t*_0_ onwards to allow the model unit membrane potentials to sufficiently depolarize (i.e., to permit the network to warm up), and over horizontal position *x* and vertical position *y*, both cropped by *c* = 3 pixels on all sides. The projection loss was also averaged over all batch samples, which we omit from the equations for brevity.

7 $${{\mathcal{L}}}^{{\rm{proj}}}=\underbrace{\frac{1}{(T - t_0+1)(H - 2c)(Q - 2c)} {\sum}_{t=t_0}^{T} {\sum}_{x=c+1}^{Q-c} {\sum}_{y=c+1}^{H-c}}_{{{\rm{Avg}}}.\,{{\rm{over}}}\,{{\rm{time}}}\,{{\rm{and}}}\,{{\rm{space}}}} \underbrace{\left({\hat{u}}_{xy}[t] - u_{xy}\left[t+T_{{\rm{off}}}^{{\rm{steps}}}\right]\right)^2}_{{\rm{MSE}}}$$

The metabolic-like loss ${{{\mathcal{L}}}}^{{{\rm{metab}}}}$ is an approximation of the biological energy use of the network. We took this to be a proxy of the energy expenditure at every synapse, calculated as the absolute input value to a synaptic weight, multiplied by its respective absolute value. Synaptic transmission has been reported to require significant energy within the cerebral cortex, with much of the energy spent on the maintenance of resting potentials^110,111^. Hence, our loss function encapsulates both the energy expenditure of resting potential maintenance and the interaction of spikes and synapses. Note that this dependence of the regularization on the absolute value of the activity means that the case where ${T}_{{{\rm{off}}}}^{{{\rm{steps}}}}=0$, the non-predictive encoding model, can be seen as a variant of a sparse autoencoder, but with a biologically-inspired sparsity objective and spiking and recurrent properties.

We weighted the metabolic loss of the graded pre-RGC-like units ${{{\mathcal{L}}}}^{{{\rm{graded}}}}$ and the spiking RGC-like units ${{{\mathcal{L}}}}^{{{\rm{spiking}}}}$ separately using hyperparameter *γ* = 0.3, as spiking neurons consume more energy than graded neurons^112,113^.

8 $${{\mathcal{L}}}^{{\rm{graded}}}=\underbrace{\frac{1}{NT} {\sum}_{i=1}^{N} {\sum}_{t=1}^{T}}_{{{\rm{Avg}}}.\,{{\rm{over}}}\,{{\rm{units}}}\,{{\rm{and}}}\,{{\rm{time}}}} \underbrace{\left(|b_i|+{\sum}_{\tau=1}^{T_E} {\sum}_{x=1}^{Q} {\sum}_{y=1}^{H} |w_{i\tau xy}| |{\tilde{u}}_{xy}[t-\tau+1]| \right)}_{{{\rm{Pre}}}-{{\rm{ganglionic}}}\,{{\rm{synaptic}}}\,{{\rm{cost}}}}$$

9 $$\begin{array}{rcl}{{{\mathcal{L}}}}^{{{\rm{spiking}}}}&=&\underbrace{\frac{1}{NT}{\sum }_{i=1}^{N}{\sum }_{t=1}^{T}}_{{{\rm{Avg}}}.{{\rm{over}}}\,{{\rm{units}}}\,{{\rm{and}}}\,{{\rm{time}}}}\underbrace{\left({\sum }_{j=1,j\ne i}^{N}| {w}_{ij}^{{{\rm{rec}}}}| {S}_{j}[t-1]\right)}_{{{\rm{Recurrent}}}\,{{\rm{synaptic}}}\,{{\rm{cost}}}}\\ &&+\underbrace{\frac{1}{TQH}{\sum }_{t=1}^{T}{\sum }_{x=1}^{Q}{\sum }_{y=1}^{H}}_{{{\rm{Avg}}}.{{\rm{over}}}\,{{\rm{time}}}\,{{\rm{and}}}\,{{\rm{space}}}}\underbrace{\left({\sum }_{i=1}^{N}{\sum }_{\tau=1}^{{T}_{D}}| {m}_{i\tau xy}| {S}_{i}[t-\tau+1]\right)}_{{{\rm{Post}}}-{{\rm{ganglionic}}}\,{{\rm{synaptic}}}\,{{\rm{cost}}}}\end{array}$$

#### Surrogate gradient descent

All spiking retinal models were optimized using a modification of the backpropagation algorithm^114^ called surrogate gradient descent^115^. To permit the flow of gradient, the gradient of the non-differentiable Heaviside step function was replaced with a well-behaved function. We adopted the fast sigmoid function, which has been shown to work well in practice^116,117^.

10 $$\frac{\partial {S}_{i}[t]}{\partial {V}_{i}[t]}={(10 \times | {V}_{i}[t]-1|+1)}^{-2}$$

All training was performed using the Adam optimizer^118^ (with default parameters) over 100 epochs, with an initial learning rate of 10^−4^ (decaying to 10^−5^ after 50 epochs) and batch size of 1024. Model weights were saved during training whenever a new minimum training loss was obtained.

### General analysis

#### Filtering for active units

For all reported results, we only included units that were active and responded to our held-out test set of natural clips. This resulted in 113/400 units being dropped from the analysis. All results that report a population percentage are with respect to the number of active units (287).

#### Firing rate traces

For all reported results, the firing rate traces, i.e., the peri-stimulus time histograms, were computed with bins of 1/240 s, corresponding to the sampling period of the stimuli, and using 8 repeats of the stimuli.

### Spike analysis

#### Firing rate

We calculated the firing rate of a neuron (and model unit) by counting the number of spikes over a given stimulus presentation and dividing this by the stimulus duration to obtain a firing rate.

#### Coefficient of variation

We computed the coefficient of variation of the interspike interval CV(ISI) to quantify the variability of the timing of spikes for a given neuron (and model unit). This is defined as the standard deviation of the interspike intervals divided by their mean^28^, where a CV(ISI) < 1 corresponds to a regular firing pattern and a CV(ISI) > 1 corresponds to an irregular firing pattern. We excluded CV(ISI) values from the analysis if there were less than three spikes for a given stimulus presentation^119^.

#### Neuron synchronization

We measured the neural synchronization between the spike trains of pairs of RGCs or pairs of model units by calculating their cross-correlogram. This is the Pearson correlation coefficient between the spike trains of two units for varying time lags. To obtain the average cross-correlogram over the population, we calculated cross-correlograms for 1000 randomly sampled unit pairs and averaged them. We binned the spike trains using a bin size of 33 ms before calculating the cross-correlograms.

#### Fano factor

We reported the Fano factor for each neuron (or model unit) over each stimulus presentation to estimate the spike count variability over the stimulus-repeat trials. The Fano factor is defined as the variance of the spike count divided by the mean spike count over stimulus-repeat trials.

#### Image decoding from single cell responses

We adopted the experimental paradigm of ref. ^23^, and obtained the activity of an OFF-type model unit in response to a 150 ms flash of a grayscale image of a swimming salamander larva. Similar to the experimental paradigm, we convolved the model unit response along the *x*- and *y*-directions (using a stride of 2 pixels) to obtain the model unit spike activity at different spatial locations. The image was then reconstructed—with every pixel denoting a unique spatial location—using either the number of spikes or the relative timing of the first spikes.

#### Image decoding from population responses to noise-corrupted inputs

We performed all analyses using a selected set of 300 natural photographs from the McGill Calibrated Color Image Database^120^. These images were converted to grayscale, as for the model training data, and normalized by subtracting the mean. The spatial dimensions were rescaled to 64 × 64 pixels using bicubic interpolation, with 240 images used for training and cross-validation, and the remaining 60 images used for testing. For every experiment, we added i.i.d. Gaussian noise $\epsilon \sim {{\mathcal{N}}}(0,{\sigma }_{s})$ to every image pixel, and varied the distribution standard deviation, *σ*_*s*_, from 0 to 2.0 in 0.25 increments between the different experiments.

We passed the set of noise-corrupted images into the model by convolving along the *x*- and *y*-directions using a stride of 4 pixels, and transformed the resulting spike outputs into a rate code (i.e., the number of spikes of a unit) and a relative spike latency code (i.e., the time of spikes minus the mean spike time across all units). We then fitted a separate linear readout model from each of these respective spike codes to reconstruct the original images. We penalized the readout weights using an *L*_1_ penalty, with the optimal penalty weighting found using five-fold cross-validation. Lastly, we fitted each model using its optimal regularization penalty on all of the training data and reported the mean pixel correlation coefficient of the reconstructed images on the held-out test set.

#### Classifying and analyzing units with an omitted-stimulus response

RGCs exhibit OSRs at the end of a dark-flash sequence around the expected time of the next dark-flash^24,25^. To determine whether a unit showed an OSR, we first found the prominent peaks in its PSTH to an OSR stimulus (Fig. 5d). We then found any peaks that came after the onset of the last dark-flash, and if there were at least two such following peaks, we considered this to be an OSR unit. The first of these peaks was taken as the response to the last dark-flash, and the second one was taken as the OSR.

### Receptive field analysis

#### Spike-triggered average

We estimated the spatiotemporal RF of every unit using a spike-triggered average, using responses to 200 white-noise input snippets of 100-frame duration. The exception was the spatiotemporal RFs in Supplementary Fig. S8, which used 100,000 white-noise input snippets of 200-frame duration. The noise snippets were generated by sampling each pixel from an i.i.d. Gaussian distribution with a mean of 0 and a standard deviation of *σ* = 10. We made the 2D spatiotemporal RFs by averaging over one of the spatial dimensions. We determined the RF temporal profile by averaging over space the pixel values for each time point in the spatiotemporal RF^121^, and the RF temporal power profile similarly but using the squared pixel values. The spatial RF was defined as the slice of the spatiotemporal RF at the time point with the highest power.

#### Fitting 2D Gaussians

For each unit, we fitted a two-dimensional Gaussian function^37,43^ to the spatial RF using gradient descent, with the function defined as:

11 $$\begin{array}{c}G(x,y)=\frac{A}{2\pi {\sigma }_{{{\rm{X}}}}{\sigma }_{{{\rm{Y}}}}\sqrt{1-{\rho }^{2}}}\exp \left(-\frac{1}{2(1-{\rho }^{2})}\right.\\ \left.\left({\left(\frac{x-{\mu }_{{{\rm{X}}}}}{{\sigma }_{{{\rm{X}}}}}\right)}^{2}-2\rho \left(\frac{x-{\mu }_{{{\rm{X}}}}}{{\sigma }_{{{\rm{X}}}}}\right)\left(\frac{y-{\mu }_{{{\rm{Y}}}}}{{\sigma }_{{{\rm{Y}}}}}\right)+{\left(\frac{y-{\mu }_{{{\rm{Y}}}}}{{\sigma }_{{{\rm{Y}}}}}\right)}^{2}\right)\right)\end{array}$$

with (*x*, *y*) denoting the spatial position in pixel space; *A* the amplitude; (*μ*_X_, *μ*_Y_) the RF center position; *σ*_X_ and *σ*_Y_ the standard deviation along the horizontal and vertical axis, respectively, and *ρ* the RF orientation as measured by the correlation coefficient. For each RF, the parameters of the two-dimensional Gaussian were fitted by minimizing the MSE between the two-dimensional Gaussian and the RF. For the RF analyses, we only included units (173/287) that had a good fit (those with a correlation coefficient >0.7 and those with standard deviation *σ*_X_ and *σ*_Y_ > 0.5 pixels^17^).

#### Classifying unit types

In a similar way to how different retinal cell types are classified^37^, we classified the functional type of every model unit that had a valid 2D Gaussian fit to its spatial RF. For every unit with a sufficient fit, we plotted its spatial RF diameter (calculated as $\sqrt{{\sigma }_{{{\rm{X}}}}{\sigma }_{{{\rm{Y}}}}}$) against the first principal component of its temporal profile. We classified the different functional unit types by grouping the units into four distinct groups using k-means clustering fixed at four centroids.

#### Mosaic analysis

We assessed the character of each cell-type mosaic by measuring its normalized nearest neighbor distribution (NNND)^49^. The measure for this distribution is calculated for each pair of nearest neighbor units in visual space. The measure is given by 2*R*/(*σ*_1_ + *σ*_2_), where *R* is the distance between the center of the spatial RF of a unit and that of its nearest neighbor, and *σ*_1_ and *σ*_2_ are the standard deviations of their respective 2D Gaussian fits along the straight line joining their centers. If the RFs touch at their standard deviation contours, then the NNND is 2; if the contours overlap, it is <2, and if they do not touch, it is >2.

### Motion tuning

#### Virtual physiology

We measured the model unit response properties to full-field sinusoidal gratings (of amplitude one) of varying orientation (0° to 360° in 5° increments), spatial frequency (10 evenly spaced values between 0.01 and 0.2 cycles per pixel), and temporal frequency (1, 2, 4 and 8 Hz). We presented each grating to each unit for 3 s and computed the resulting PSTH by averaging over 8 repeats and convolving with a Gaussian kernel (*σ* = 37 ms). Next, we computed the mean firing rate over time (ignoring the first 41 ms for network warmup, as done in training), resulting in a 3D mean-firing tuning space for each unit, with the preferred stimulus being the one eliciting the highest mean response.

#### Orientation and direction selectivity

We measured the orientation selectivity index (OSI) and direction selectivity index (DSI) for each unit, which respectively quantify the tuning preference for motion across a particular orientation and direction:

12 $$\,{\mbox{OSI}}\,=\frac{{r}_{{{\rm{pref}}}}^{{{\rm{orient}}}}-{r}_{{{\rm{orth}}}}^{{{\rm{orient}}}}}{{r}_{{{\rm{pref}}}}^{{{\rm{orient}}}}+{r}_{{{\rm{orth}}}}^{{{\rm{orient}}}}}$$

13 $$\,{\mbox{DSI}}\,=\frac{{r}_{{{\rm{pref}}}}^{{{\rm{dir}}}}-{r}_{{{\rm{non}}}\,{{\rm{pref}}}}^{{{\rm{dir}}}}}{{r}_{{{\rm{pref}}}}^{{{\rm{dir}}}}+{r}_{{{\rm{non}}}\,{{\rm{pref}}}}^{{{\rm{dir}}}}}$$

with ${r}_{{{\rm{pref}}}}^{{{\rm{orient}}}}={r}_{{{\rm{pref}}}}^{{{\rm{dir}}}}$ being a unit’s response to a stimulus moving in the preferred direction, and ${r}_{{{\rm{orth}}}}^{{{\rm{orient}}}}$ and ${r}_{{{\rm{non}}}\,{{\rm{pref}}}}^{{{\rm{dir}}}}$ being the responses to the orthogonal orientation and opposite direction, respectively (all using a unit’s preferred spatial and temporal frequency). For both metrics, a value close to zero corresponds to weaker tuning, and a value close to unity corresponds to stronger tuning.

#### Counting Y-type-like units

We presented full-field sinusoidal gratings with a spatial frequency of 0.3 cycles per pixel at each unit’s preferred orientation. We then measured each unit’s firing rate for the static grating *r*_SG_ and the grating moving at the unit’s preferred temporal frequency *r*_MG_. We considered a unit to be Y-type-like if the following constraints were satisfied: the moving grating response was larger than the static grating response (*r*_MG_ > *r*_SG_), and the static and moving grating responses were respectively below (*r*_SG_ < 24 spikes/s) and above (*r*_MG_ > 24 spikes/s) a firing threshold (chosen via qualitative inspection), to potentially avoid counting units as Y-type-like due to noise fluctuations.

#### Object motion stimuli

We implemented two types of object motion stimulus pairs. In the first type (Fig. 4c), the stimulus was either a global smoothly moving grating or the same grating moving only in a small circular area centered on the RF of the relevant unit, but masked with gray outside that area. In the second type (Fig. 4c), the stimulus had the same grating moving with random jittered motion both inside and outside the circular area, barring a thin gray annulus, but the random movement was either the same (coherent) or independent (differential) between the inside and outside of the area. For the second stimulus type, we quantified the object motion sensitivity of each unit using the object motion sensitivity index; this is given by (*r*_dif_ − *r*_coh_)/(*r*_dif_ + *r*_coh_), where *r*_dif_ is the firing rate for the differential stimulus, and *r*_coh_ is the firing rate to the coherent stimulus. To calculate the index, we average over 10 different pairs of the stimuli, each pair using different random seeds and lasting 200 s duration. We then averaged over units.

#### Anticipation code

We implemented the experimental protocol of ref. ^26^. We replicated this experiment for the model by projecting a vertical dark bar (pixel value of −1.5) on a white background (assuming, due to retinal adaptation, a pixel value of 0), moving rightwards at a range of speeds from 0.05 to 1 pixels/frame.

### Predicting neural responses from model activity

We used publicly available and spike-sorted recordings of RGC activity in isolated retinal tissue from different animal species in response to natural images and movies. In each case, these recordings were made in accordance with national and local institutional guidelines and approved by institutional animal care and use committees, as set out in the individual publications. For all neural datasets, we used 80% of the data for training and the remaining 20% for testing (with splits separated by unique clips or images). As the datasets were recorded at different sampling rates, we resampled all data to be 240 Hz (the same sampling rate of the natural movie stimulus with which the spiking retinal model was trained) by repeating frames at particular points in time. We also standardized all natural stimuli (clips and images) by subtracting the mean and dividing by the standard deviation of the training set. We averaged the recorded spike responses over trials and convolved the resulting PSTH with a Gaussian kernel (using *σ* = 38 ms, 9 time-steps) to obtain a smoothed spike firing probability for every neuron^39^.

#### Macaque image and movie dataset

We used recordings from ON- and OFF-parasol cells in the isolated peripheral retina of male and female macaque monkeys in response to natural images and movies (made available by ref. ^38^). This consisted of 16 cells recorded in response to 48 250 ms-flashed natural images over 5 repeats, and 24 cells recorded in response to 7 6 s-long natural movies over 4 repeats, with the movies consisting of the natural images spatially shifted in time using eye movement trajectories of human observers^122^. All stimuli were projected at a frame rate of 60 Hz onto the RF center of each neuron using a circular aperture.

#### Marmoset movie dataset

We used recordings from ON- and OFF-midget and ON- and OFF-parasol cells in the peripheral retina of male marmoset monkeys in response to natural movies (made available by ref. ^39^). This includes 423 cells recorded in response to 22 1 s-long natural movies over 30 trials, with the movies consisting of the natural images spatially shifted in time using eye movement trajectories of awake, head-fixed marmoset monkeys. All stimuli were projected at a frame rate of 85 Hz. To keep the fitting procedure computationally manageable, we only fitted a subset of the most responsive neurons, as measured by the maximum obtainable correlation coefficient (see below), resulting in 164 neurons (using ${{\mbox{CC}}}_{\max }\ge 0.93$).

#### Mouse movie dataset

We used recordings from ON- and OFF-transient and ON- and OFF-sustained alpha cells in isolated retinal pieces from female mice in response to natural movies (made available by ref. ^39^). These 1791 cells were recorded in response to 22 1 s-long natural movies over 30 trials, with the movies consisting of the natural images spatially shifted in time using the horizontal gaze component of freely moving mice. All stimuli were projected at a frame rate of 75 Hz. Again, to keep the fitting procedure computationally manageable, we only fitted a subset of the most responsive neurons, as measured by the maximum obtainable correlation coefficient (see below), resulting in 351 neurons (using ${{\mbox{CC}}}_{\max }\ge 0.93$).

#### Salamander image dataset

We used recordings of various ON- and OFF-type cells in the isolated retina of male and female salamanders in response to natural images (made available by ref. ^37^). This includes 215 cells recorded in response to 300 natural images flashed for 200 ms each over 12 trials. There was an 800 ms inter-stimulus interval between image presentations, with spike responses to each image recorded between image onset and 100 ms after image offset. The images came from the Van Hateren database^123^ over 12 trials. All stimuli were projected at a frame rate of 30 Hz.

#### Obtaining latent model activity

We trained a set of 14 retinal models, one for every temporal offset *T*_off_ into the future (*T*_off_ ∈ {0, 8, 16, 24, 32, 40, 48, 56, 64, 72, 80, 96, 112, 128} ms, where *T*_off_ = 0 corresponds to the present). For each neural dataset, we convolved each retinal model (using a stride of 4 pixels) over the spatial dimensions of the dataset’s stimuli to obtain the model unit spike activity ${{\bf{D}}}[t]\in {{\mathbb{R}}}^{N\times \tilde{Q}\times \tilde{H}}$ in response to the stimuli. The number of simulation steps $\tilde{T}$, and the spatial width $\tilde{Q}$ and height $\tilde{H}$ dimensions varied between the different datasets. This was repeated 8 times, and averaged over repeats, to obtain unit responses ${{{\bf{D}}}}^{{{\rm{prob}}}}[t]\in {{\mathbb{R}}}^{N\times \tilde{Q}\times \tilde{H}}$ consisting of the spike probability of every unit over space at each time step. We further compressed these responses to a latent activity array ${{{\bf{D}}}}^{{{\rm{latent}}}}[t]\in {{\mathbb{R}}}^{500}$ using the first *N*_PC_ = 500 principal components over space and units. Each element of **D**^latent^ is given by ${d}_{k}^{{{\rm{latent}}}}[t]$. This technique is routinely used^77^, as it reduces overfitting^77^ and keeps the fitting procedure computationally tractable^124^.

#### Fitting procedure

For every retinal model, we fitted a linear-non-linear readout to predict the response profile *r*_*n*_[*t*] of every neuron *n* in every dataset from the latent model activity. During project development, using only the training set, we explored various hyperparameters relating to the readout model and fitting procedure for each dataset and employed those hyperparameters that we found to work the best.

14 $${\hat{r}}_{n}[t]=\exp \left({b}_{n}^{{{\rm{read}}}}+{\sum }_{\tau=1}^{{T}_{R}}{\sum }_{k=1}^{{N}_{{{\rm{PC}}}}}{w}_{nk\tau }^{{{\rm{read}}}}{d}_{k}^{{{\rm{latent}}}}[t-\tau+1]\right)$$

with readout weights ${{{\bf{W}}}}_{n}^{{{\rm{read}}}}\in {{\mathbb{R}}}^{{N}_{{{\rm{PC}}}}\times {T}_{R}}$ using a *T*_*R*_ = 5 temporal frame span equivalent to ≈21 ms (except for the mouse movie dataset where we used a span of 46 ms). Each element of ${{{\bf{W}}}}_{n}^{{{\rm{read}}}}$ is given by ${w}_{nk\tau }^{{{\rm{read}}}}$. Each readout was jointly fitted across all $\tilde{N}$ neurons of a dataset by minimizing the negative Poisson log-likelihood of the readout predictions ${\hat{r}}_{n}[t]$ and the neuron’s responses *r*_*n*_[*t*], and an *L*_1_ penalty on the readout weights (weighted by hyperparameter *λ*^read^) to avoid overfitting.

15 $${{{\mathcal{L}}}}^{{{\rm{out}}}}=-\frac{1}{\tilde{N}\tilde{T}}{\sum }_{n=1}^{\tilde{N}}{\sum }_{t=1}^{\tilde{T}}\left({r}_{n}[t]\ln ({\hat{r}}_{n}[t])-{\hat{r}}_{n}[t]\right)+{\lambda }^{{{\rm{read}}}}{\sum }_{n=1}^{\tilde{N}}{\sum }_{\tau=1}^{{T}_{R}}{\sum }_{k=1}^{{N}_{{{\rm{PC}}}}}\left\vert {w}_{nk\tau }^{{{\rm{read}}}}\right\vert$$

We trained each readout using the Adam optimizer (with default parameters) at a learning rate of 10^−4^ over 2000 epochs with a batch size of 8 subsamples of at most 600 ms duration (except for the salamander dataset where we used a batch size of 32). We found the optimal regularization hyperparameter *λ*^read^ using five-fold cross-validation on the training dataset (over a set of five different log-spaced values, which we varied between the different datasets). Lastly, we also explored different spatial scales of the input stimuli to account for differences in spatial scale between the model units and neurons^125,126^. To determine the optimal scale for each dataset, we ran all fits using three different spatial scales (×0.66, ×1, and ×1.5) using the retinal model optimized for efficient compression of the present (Supplementary Table S1). We then chose the spatial scale that performed the best on each test dataset to fit the remaining models.

#### Normalized and maximum correlation coefficient

We used the normalized correlation coefficient CC_norm_ to quantify the neural fits, expressed as the Pearson correlation coefficient, CC, between the model’s prediction and target neural response, normalized by dividing by the maximum obtainable correlation coefficient, ${{\mbox{CC}}}_{\max }$, to take into account neural noise^76,127^. A value of zero indicates no correlation between the predicted and target neural response, whereas a value of one indicates the best possible score.

### Reporting summary

Further information on research design is available in the Nature Portfolio Reporting Summary linked to this article.

## Supplementary information

Supplementary Information

Reporting Summary

Transparent Peer Review file

## Data Availability

The natural movie training data recorded for this study have been deposited in the Figshare repository^128^. Pre-trained spiking models can be found at ref. ^129^. We did not perform any of the retinal recordings. Inspect the data availability sections in the relevant papers cited in this manuscript to access the retinal data.

## Code availability

The code of the spiking retinal model and the code for reproducing the results can be found at ref. ^129^. The code was mostly written in Python (3:10) using Jupyter Notebook (6.0.3), with a very small amount of MATLAB (R2025b). We used PyTorch (2.1.2)^130^ and DevTorch (0.0.5)^131^ for implementing the models and BrainBox (0.0.9)^132^ for analyzing the spike statistics, receptive fields, and tuning properties. The code was run on an Ubuntu desktop using an NVIDIA GeForce RTX 3090 GPU.

## References

[1] Hartline, H. K. The response of single optic nerve fibers of the vertebrate eye to illumination of the retina. *Am. J. Physiol.* **121**, 400–415 (1938).

[2] Kuffler, S. W. Discharge patterns and functional organization of mammalian retina. *J. Neurophysiol.* **16**, 37–68 (1953). 10.1152/jn.1953.16.1.37 13035466

[3] Meister, M. & Berry, M. J. The neural code of the retina. *Neuron* **22**, 435–450 (1999). 10.1016/s0896-6273(00)80700-x 10197525

[4] Ocko, S., Lindsey, J., Ganguli, S. & Deny, S. The emergence of multiple retinal cell types through efficient coding of natural movies. Preprint at *bioRxiv* 10.1101/458737 (2018).

[5] Jun, N. Y., Field, G. D. & Pearson, J. Scene statistics and noise determine the relative arrangement of receptive field mosaics. *Proc. Natl. Acad. Sci. USA* **118**, e2105115118 (2021). 10.1073/pnas.2105115118 PMC8488585 34556573

[6] Jun, N. Y., Field, G. & Pearson, J. Efficient coding, channel capacity, and the emergence of retinal mosaics. *Adv. Neural Inf. Process. Syst.* **35**, 32311–32324 (2022). PMC10168625 37168261

[7] Karklin, Y. & Simoncelli, E. Efficient coding of natural images with a population of noisy linear-nonlinear neurons. *Adv. Neural Inf. Process. Syst.* **24**, 999–1007 (2011). PMC4532291 26273180

[8] Roy, S., Jun, N. Y., Davis, E. L., Pearson, J. & Field, G. D. Inter-mosaic coordination of retinal receptive fields. *Nature* **592**, 409–413 (2021). 10.1038/s41586-021-03317-5 PMC8049984 33692544

[9] Zhaoping, L. Theoretical understanding of the early visual processes by data compression and data selection. *Netw. Comput. Neural Syst.* **17**, 301–334 (2006). 10.1080/09548980600931995 17283516

[10] Barlow, H. B. et al. Possible principles underlying the transformation of sensory messages. *Sens. Commun.* **1**, 217–233 (1961).

[11] Palmer, S. E., Marre, O., Berry, M. J. & Bialek, W. Predictive information in a sensory population. *Proc. Natl. Acad. Sci. USA* **112**, 6908–6913 (2015). 10.1073/pnas.1506855112 PMC4460449 26038544

[12] Salisbury, J. M. & Palmer, S. E. Optimal prediction in the retina and natural motion statistics. *J. Stat. Phys.* **162**, 1309–1323 (2016).

[13] Liu, B., Hong, A., Rieke, F. & Manookin, M. B. Predictive encoding of motion begins in the primate retina. *Nat. Neurosci.* **24**, 1280–1291 (2021). 10.1038/s41593-021-00899-1 PMC8728393 34341586

[14] Berry, M. J. & Schwartz, G. The retina as embodying predictions about the visual world. In * Predictions in the Brain: Using our Past to Generate a Future* 295–310 (ed. Bar, M) (Oxford Academic, 2011).

[15] Singer, Y., Taylor, L., Willmore, B. D., King, A. J. & Harper, N. S. Hierarchical temporal prediction captures motion processing along the visual pathway. *eLife* **12**, e52599 (2023). 10.7554/eLife.52599 PMC10629830 37844199

[16] Bialek, W., Nemenman, I. & Tishby, N. Predictability, complexity, and learning. *Neural Comput.* **13**, 2409–2463 (2001). 10.1162/089976601753195969 11674845

[17] Singer, Y. et al. Sensory cortex is optimized for prediction of future input. *eLife* **7**, e31557 (2018). 10.7554/eLife.31557 PMC6108826 29911971

[18] Keller, G. B. & Mrsic-Flogel, T. D. Predictive processing: a canonical cortical computation. *Neuron* **100**, 424–435 (2018). 10.1016/j.neuron.2018.10.003 PMC6400266 30359606

[19] Chalk, M., Marre, O. & Tkačik, G. Toward a unified theory of efficient, predictive, and sparse coding. *Proc. Natl. Acad. Sci. USA* **115**, 186–191 (2018). 10.1073/pnas.1711114115 PMC5776796 29259111

[20] Dayan, P. & Abbott, L. F. *Theoretical Neuroscience: Computational and Mathematical Modeling of Neural Systems* (Computational Neuroscience Series, MIT Press, 2001).

[21] Levenstein, D. et al. On the role of theory and modeling in neuroscience. *J. Neurosci.* **43**, 1074–1088 (2023). 10.1523/JNEUROSCI.1179-22.2022 PMC9962842 36796842

[22] Gollisch, T. & Meister, M. Eye smarter than scientists believed: neural computations in circuits of the retina. *Neuron* **65**, 150–164 (2010). 10.1016/j.neuron.2009.12.009 PMC3717333 20152123

[23] Gollisch, T. & Meister, M. Rapid neural coding in the retina with relative spike latencies. *Science* **319**, 1108–1111 (2008). 10.1126/science.1149639 18292344

[24] Schwartz, G., Harris, R., Shrom, D. & Berry, M. J. Detection and prediction of periodic patterns by the retina. *Nat. Neurosci.* **10**, 552–554 (2007). 10.1038/nn1887 PMC3196557 17450138

[25] Schwartz, G. & Berry 2nd, M. J. Sophisticated temporal pattern recognition in retinal ganglion cells. *J. Neurophysiol.* **99**, 1787–1798 (2008). 10.1152/jn.01025.2007 PMC2856926 18272878

[26] Berry, M. J., Brivanlou, I. H., Jordan, T. A. & Meister, M. Anticipation of moving stimuli by the retina. *Nature* **398**, 334–338 (1999). 10.1038/18678 10192333

[27] Ölveczky, B. P., Baccus, S. A. & Meister, M. Segregation of object and background motion in the retina. *Nature* **423**, 401–408 (2003). 10.1038/nature01652 12754524

[28] Gerstner, W., Kistler, W. M., Naud, R. & Paninski, L. *Neuronal Dynamics: From Single Neurons to Networks and Models of Cognition* (Cambridge University Press, 2014).

[29] Baccus, S. A. & Meister, M. Fast and slow contrast adaptation in retinal circuitry. *Neuron* **36**, 909–919 (2002). 10.1016/s0896-6273(02)01050-4 12467594

[30] Pillow, J. W. et al. Spatio-temporal correlations and visual signalling in a complete neuronal population. *Nature* **454**, 995–999 (2008). 10.1038/nature07140 PMC2684455 18650810

[31] Vaney, D. I. Patterns of neuronal coupling in the retina. *Prog. Retin. Eye Res.* **13**, 301–355 (1994).

[32] Vlasiuk, A. & Asari, H. Feedback from retinal ganglion cells to the inner retina. *PLoS ONE* **16**, e0254611 (2021). 10.1371/journal.pone.0254611 PMC8297895 34292988

[33] Baylor, D., Matthews, G. & Yau, K. Two components of electrical dark noise in toad retinal rod outer segments. * J. Physiol.* **309**, 591–621 (1980). 10.1113/jphysiol.1980.sp013529 PMC1274605 6788941

[34] van Rossum, M. C., O’Brien, B. J. & Smith, R. G. Effects of noise on the spike timing precision of retinal ganglion cells. *J. Neurophysiol.* **89**, 2406–2419 (2003). 10.1152/jn.01106.2002 12740401

[35] Berry, M. J., Warland, D. K. & Meister, M. The structure and precision of retinal spike trains. *Proc. Natl. Acad. Sci. USA* **94**, 5411–5416 (1997). 10.1073/pnas.94.10.5411 PMC24692 9144251

[36] Berry, M. & Meister, M. Refractoriness and neural precision. *J. Neurosci.* **18**, 2200–2211 (1998). 10.1523/JNEUROSCI.18-06-02200.1998 PMC6792934 9482804

[37] Liu, J. K., Karamanlis, D. & Gollisch, T. Simple model for encoding natural images by retinal ganglion cells with nonlinear spatial integration. *PLoS Comput. Biol.* **18**, e1009925 (2022). 10.1371/journal.pcbi.1009925 PMC8932571 35259159

[38] Freedland, J. & Rieke, F. Systematic reduction of the dimensionality of natural scenes allows accurate predictions of retinal ganglion cell spike outputs. *Proc. Natl. Acad. Sci. USA* **119**, e2121744119 (2022). 10.1073/pnas.2121744119 PMC9674269 36343230

[39] Karamanlis, D. et al. Nonlinear receptive fields evoke redundant retinal coding of natural scenes. *Nature* **637**, 394–401 (2025). 10.1038/s41586-024-08212-3 PMC11711096 39567692

[40] Dacey, D. M. The mosaic of midget ganglion cells in the human retina. *J. Neurosci.* **13**, 5334–5355 (1993). 10.1523/JNEUROSCI.13-12-05334.1993 PMC6576399 8254378

[41] Dacey, D. Origins of perception: retinal ganglion cell diversity and the creation of parallel visual pathways. In Gazzaniga, M.S. (ed.)* The Cognitive Neurosciences III* 281–301 (2004).

[42] Soto, F. et al. Efficient coding by midget and parasol ganglion cells in the human retina. *Neuron* **107**, 656–666 (2020). 10.1016/j.neuron.2020.05.030 PMC7442669 32533915

[43] Kling, A. et al. Functional organization of midget and parasol ganglion cells in the human retina. Preprint at *bioRxiv* 10.1101/2020.08.07.240762 (2020).

[44] Lee, B. B. Receptive field structure in the primate retina. *Vision Res.* **36**, 631–644 (1996). 10.1016/0042-6989(95)00167-0 8762295

[45] Field, G. D. et al. Spatial properties and functional organization of small bistratified ganglion cells in primate retina. *J. Neurosci.* **27**, 13261–13272 (2007). 10.1523/JNEUROSCI.3437-07.2007 PMC6673390 18045920

[46] Devries, S. H. & Baylor, D. A. Mosaic arrangement of ganglion cell receptive fields in rabbit retina. *J. Neurophysiol.* **78**, 2048–2060 (1997). 10.1152/jn.1997.78.4.2048 9325372

[47] Wässle, H., Peichl, L. & Boycott, B. B. Morphology and topography of on-and off-alpha cells in the cat retina. *Proc. R. Soc. Lond. Ser. B Biol. Sci.* **212**, 157–175 (1981). 10.1098/rspb.1981.0032 6166012

[48] Field, G. D. & Chichilnisky, E. Information processing in the primate retina: circuitry and coding. *Annu. Rev. Neurosci.* **30**, 1–30 (2007). 10.1146/annurev.neuro.30.051606.094252 17335403

[49] Ravi, S., Ahn, D., Greschner, M., Chichilnisky, E. & Field, G. D. Pathway-specific asymmetries between on and off visual signals. *J. Neurosci.* **38**, 9728–9740 (2018). 10.1523/JNEUROSCI.2008-18.2018 PMC6222062 30249795

[50] Dacey, D. M. Physiology, morphology and spatial densities of identified ganglion cell types in primate retina. In Bock, G. R. & Goode, J. A. (eds.) *Proc. Ciba Foundation Symposium 184-Higher-Order Processing in the Visual System: Higher-Order Processing in the Visual System* 12–28 (Wiley, 2007). 10.1002/9780470514610.ch2 7882750

[51] Ratliff, C. P., Borghuis, B. G., Kao, Y.-H., Sterling, P. & Balasubramanian, V. Retina is structured to process an excess of darkness in natural scenes. *Proc. Natl. Acad. Sci. USA* **107**, 17368–17373 (2010). 10.1073/pnas.1005846107 PMC2951394 20855627

[52] O’Brien, B. J., Isayama, T., Richardson, R. & Berson, D. M. Intrinsic physiological properties of cat retinal ganglion cells. * J. Physiol.* **538**, 787–802 (2002). 10.1113/jphysiol.2001.013009 PMC2290089 11826165

[53] Borghuis, B. G., Ratliff, C. P. & Smith, R. G. Impact of light-adaptive mechanisms on mammalian retinal visual encoding at high light levels. *J. Neurophysiol.* **119**, 1437–1449 (2018). 10.1152/jn.00682.2017 PMC5966735 29357459

[54] Kaplan, E. & Benardete, E. The dynamics of primate retinal ganglion cells. *Prog. Brain Res.* **134**, 17–34 (2001). 10.1016/s0079-6123(01)34003-7 11702542

[55] Sabbah, S. et al. A retinal code for motion along the gravitational and body axes. *Nature* **546**, 492–497 (2017). 10.1038/nature22818 PMC5729591 28607486

[56] Oyster, C. W. & Barlow, H. B. Direction-selective units in rabbit retina: distribution of preferred directions. *Science* **155**, 841–842 (1967). 10.1126/science.155.3764.841 6019094

[57] Kühn, N. K. & Gollisch, T. Joint encoding of object motion and motion direction in the salamander retina. *J. Neurosci.* **36**, 12203–12216 (2016). 10.1523/JNEUROSCI.1971-16.2016 PMC6601980 27903729

[58] Maximov, V., Maximova, E. & Maximov, P. Direction selectivity in the goldfish tectum revisited. *Ann. N. Y. Acad. Sci.* **1048**, 198–205 (2005). 10.1196/annals.1342.018 16154933

[59] Bowling, D. Light responses of ganglion cells in the retina of the turtle. * J. Physiol.* **299**, 173–196 (1980). 10.1113/jphysiol.1980.sp013118 PMC1279218 7381765

[60] Nath, A. & Schwartz, G. W. Cardinal orientation selectivity is represented by two distinct ganglion cell types in mouse retina. *J. Neurosci.* **36**, 3208–3221 (2016). 10.1523/JNEUROSCI.4554-15.2016 PMC4792935 26985031

[61] Levick, W. R. Receptive fields and trigger features of ganglion cells in the visual streak of the rabbit’s retina. * J. Physiol.* **188**, 285 (1967). 10.1113/jphysiol.1967.sp008140 PMC1396015 6032202

[62] Amthor, F. R., Takahashi, E. S. & Oyster, C. W. Morphologies of rabbit retinal ganglion cells with complex receptive fields. *J. Comp. Neurol.* **280**, 97–121 (1989). 10.1002/cne.902800108 2918098

[63] Rosenberg, A. & Talebi, V. The primate retina contains distinct types of Y-like ganglion cells. *J. Neurosci.* **29**, 5048–5050 (2009). 10.1523/JNEUROSCI.0423-09.2009 PMC6665473 19386900

[64] Enroth-Cugell, C. & Robson, J. G. The contrast sensitivity of retinal ganglion cells of the cat. * J. Physiol.* **187**, 517–552 (1966). 10.1113/jphysiol.1966.sp008107 PMC1395960 16783910

[65] Caldwell, J. & Daw, N. New properties of rabbit retinal ganglion cells. * J. Physiol.* **276**, 257–276 (1978). 10.1113/jphysiol.1978.sp012232 PMC1282423 650447

[66] Petrusca, D. et al. Identification and characterization of a Y-like primate retinal ganglion cell type. *J. Neurosci.* **27**, 11019–11027 (2007). 10.1523/JNEUROSCI.2836-07.2007 PMC6672861 17928443

[67] Lettvin, J. Y., Maturana, H. R., McCulloch, W. S. & Pitts, W. H. What the frog’s eye tells the frog’s brain. *Proc. IRE* **47**, 1940–1951 (1959).

[68] Baccus, S. A., Ölveczky, B. P., Manu, M. & Meister, M. A retinal circuit that computes object motion. *J. Neurosci.* **28**, 6807–6817 (2008). 10.1523/JNEUROSCI.4206-07.2008 PMC6670970 18596156

[69] Dayan, P. & Abbott, L. F. *Theoretical Neuroscience: Computational and Mathematical Modeling of Neural Systems* (MIT Press, 2005).

[70] Stanley, G. B. Reading and writing the neural code. *Nat. Neurosci.* **16**, 259–263 (2013). 10.1038/nn.3330 23434978

[71] Judge, S. J., Richmond, B. J. & Chu, F. C. Implantation of magnetic search coils for measurement of eye position: an improved method. *Vision Res.* **20**, 535–538 (1980). 10.1016/0042-6989(80)90128-5 6776685

[72] Martinez-Conde, S., Macknik, S. L. & Hubel, D. H. The role of fixational eye movements in visual perception. *Nat. Rev. Neurosci.* **5**, 229–240 (2004). 10.1038/nrn1348 14976522

[73] Karamanlis, D., Schreyer, H. M. & Gollisch, T. Retinal encoding of natural scenes. *Annu. Rev. Vis. Sci.* **8**, 171–193 (2022). 10.1146/annurev-vision-100820-114239 35676096

[74] Sterling, P. & Laughlin, S. *Principles of Neural Design* (MIT Press, 2015).

[75] Auksztulewicz, R., Rajendran, V. G., Peng, F., Schnupp, J. W. H. & Harper, N. S. Omission responses in local field potentials in rat auditory cortex. *BMC Biol.* **21**, 130 (2023). 10.1186/s12915-023-01592-4 PMC10230691 37254137

[76] Schoppe, O., Harper, N. S., Willmore, B. D., King, A. J. & Schnupp, J. W. Measuring the performance of neural models. *Front. Comput. Neurosci.* **10**, 10 (2016). 10.3389/fncom.2016.00010 PMC4748266 26903851

[77] Schrimpf, M. et al. Brain-score: Which artificial neural network for object recognition is most brain-like? Preprint at *bioRxiv* 10.1101/407007 (2018).

[78] Kerschensteiner, D. Feature detection by retinal ganglion cells. *Annu. Rev. Vis. Sci.* **8**, 135–169 (2022). 10.1146/annurev-vision-100419-112009 35385673

[79] Tanaka, H. et al. From deep learning to mechanistic understanding in neuroscience: the structure of retinal prediction. *Adv. Neural Inf. Process. Syst.* **32**, 8537–8547 (2019). PMC8916592 35283616

[80] Maheswaranathan, N. et al. Interpreting the retinal neural code for natural scenes: from computations to neurons. *Neuron* **111**, 2742–2755 (2023). 10.1016/j.neuron.2023.06.007 PMC10680974 37451264

[81] Schnapf, J., Kraft, T. & Baylor, D. Spectral sensitivity of human cone photoreceptors. *Nature* **325**, 439–441 (1987). 10.1038/325439a0 3808045

[82] Donner, K. Temporal vision: measures, mechanisms and meaning. *J. Exp. Biol.* **224**, jeb222679 (2021). 10.1242/jeb.222679 PMC8353166 34328511

[83] Bixler, E. O., Bartlett, N. R. & Lansing, R. W. Latency of the blink reflex and stimulus intensity. *Percept. Psychophys.* **2**, 559–560 (1967).

[84] Shapley, R. M. & Victor, J. D. The effect of contrast on the transfer properties of cat retinal ganglion cells. * J. Physiol.* **285**, 275–298 (1978). 10.1113/jphysiol.1978.sp012571 PMC1281756 745079

[85] Kim, K. J. & Rieke, F. Temporal contrast adaptation in the input and output signals of salamander retinal ganglion cells. *J. Neurosci.* **21**, 287–299 (2001). 10.1523/JNEUROSCI.21-01-00287.2001 PMC6762442 11150346

[86] Taylor, L., King, A. & Harper, N. S. Addressing the speed-accuracy simulation trade-off for adaptive spiking neurons. *Adv. Neural Inf. Process. Syst.* **36**, 59360–59374 (2023).

[87] Nategh, N., Manu, M. & Baccus, S. A. Modulation of input sensitivity and output gain by retinal amacrine cells. *eLife* **12**, RP90862 (2023).

[88] Schreyer, H. M. & Gollisch, T. Nonlinear spatial integration in retinal bipolar cells shapes the encoding of artificial and natural stimuli. *Neuron* **109**, 1692–1706 (2021). 10.1016/j.neuron.2021.03.015 PMC8153253 33798407

[89] Idrees, S., Manookin, M. B., Rieke, F., Field, G. D. & Zylberberg, J. Biophysical neural adaptation mechanisms enable artificial neural networks to capture dynamic retinal computation. *Nat. Commun.* **15**, 5957 (2024). 10.1038/s41467-024-50114-5 PMC11251147 39009568

[90] Dacey, D. M. & Packer, O. S. Colour coding in the primate retina: diverse cell types and cone-specific circuitry. *Curr. Opin. Neurobiol.* **13**, 421–427 (2003). 10.1016/s0959-4388(03)00103-x 12965288

[91] Krizhevsky, A., Sutskever, I. & Hinton, G. E. Imagenet classification with deep convolutional neural networks. *Adv. Neural Inf. Process. Syst.* **25**, 1097–1105 (2012).

[92] Baden, T., Euler, T. & Berens, P. Understanding the retinal basis of vision across species. *Nat. Rev. Neurosci.* **21**, 5–20 (2020). 10.1038/s41583-019-0242-1 31780820

[93] Perez-Nieves, N. & Goodman, D. Sparse spiking gradient descent. *Adv. Neural Inf. Process. Syst.* **34**, 11795–11808 (2021).

[94] Chichilnisky, E. & Kalmar, R. S. Functional asymmetries in on and off ganglion cells of primate retina. *J. Neurosci.* **22**, 2737–2747 (2002). 10.1523/JNEUROSCI.22-07-02737.2002 PMC6758315 11923439

[95] Shi, Q., Gupta, P., Boukhvalova, A. K., Singer, J. H. & Butts, D. A. Functional characterization of retinal ganglion cells using tailored nonlinear modeling. *Sci. Rep.* **9**, 8713 (2019). 10.1038/s41598-019-45048-8 PMC6581951 31213620

[96] Sherman, S. M. & Spear, P. D. Organization of visual pathways in normal and visually deprived cats. *Physiol. Rev.* **62**, 738–855 (1982). 10.1152/physrev.1982.62.2.738 6280221

[97] Watanabe, M. & Rodieck, R. Parasol and midget ganglion cells of the primate retina. *J. Comp. Neurol.* **289**, 434–454 (1989). 10.1002/cne.902890308 2808778

[98] Goetz, J. et al. Unified classification of mouse retinal ganglion cells using function, morphology, and gene expression. *Cell Rep.* **40**, 111040 (2022). 10.1016/j.celrep.2022.111040 PMC9364428 35830791

[99] Dhande, O. S. et al. Molecular fingerprinting of on–off direction-selective retinal ganglion cells across species and relevance to primate visual circuits. *J. Neurosci.* **39**, 78–95 (2019). 10.1523/JNEUROSCI.1784-18.2018 PMC6325260 30377226

[100] Parikh, N., Itti, L. & Weiland, J. Saliency-based image processing for retinal prostheses. *J. Neural Eng.* **7**, 016006 (2010). 10.1088/1741-2560/7/1/016006 20075505

[101] Feng, C., Dai, S., Zhao, Y. & Liu, S. Edge-preserving image decomposition based on saliency map. In *Proc. 7th International Congress on Image and Signal Processing* 159–163 (IEEE, 2014).

[102] Erickson-Davis, C. & Korzybska, H. What do blind people “see” with retinal prostheses? Observations and qualitative reports of epiretinal implant users. *PLoS ONE* **16**, e0229189 (2021). 10.1371/journal.pone.0229189 PMC7875418 33566851

[103] Klavinskis-Whiting, S. et al. Prediction of future input explains lateral connectivity in primary visual cortex. *Curr. Biol.* **35**, 530–541 (2025). 10.1016/j.cub.2024.11.073 PMC7617481 39798566

[104] Klavinskis-Whiting, S., King, A. J. & Harper, N. S. Hierarchical recurrent temporal prediction as a model of the mammalian dorsal visual pathway. *PLOS Comput. Biol.* **22**, e1013138 (2026). 10.1371/journal.pcbi.1013138 PMC13252843 42207826

[105] Taylor, L. & Nitschke, G. Improving deep learning with generic data augmentation.* IEEE Symposium Series on Computational Intelligence*,1542–1547 (2018).

[106] Lee, S. et al. An unconventional glutamatergic circuit in the retina formed by vGluT3 amacrine cells. *Neuron* **84**, 708–715 (2014). 10.1016/j.neuron.2014.10.021 PMC4254642 25456497

[107] Demb, J. B. & Singer, J. H. Functional circuitry of the retina. *Annu. Rev. Vis. Sci.* **1**, 263–289 (2015). 10.1146/annurev-vision-082114-035334 PMC5749398 28532365

[108] Weber, A. I., Shea-Brown, E. & Rieke, F. Identification of multiple noise sources improves estimation of neural responses across stimulus conditions. *eNeuro* **8**, ENEURO.0191 (2021). 10.1523/ENEURO.0191-21.2021 PMC8260275 34083382

[109] Perez-Nieves, N., Leung, V. C., Dragotti, P. L. & Goodman, D. F. Neural heterogeneity promotes robust learning. *Nat. Commun.* **12**, 5791 (2021). 10.1038/s41467-021-26022-3 PMC8490404 34608134

[110] Faria-Pereira, A. & Morais, V. A. Synapses: the brain’s energy-demanding sites. *Int. J. Mol. Sci.* **23**, 3627 (2022). 10.3390/ijms23073627 PMC8998888 35408993

[111] Harris, J. J., Jolivet, R. & Attwell, D. Synaptic energy use and supply. *Neuron* **75**, 762–777 (2012). 10.1016/j.neuron.2012.08.019 22958818

[112] Laughlin, S. B., de Ruyter van Steveninck, R. R. & Anderson, J. C. The metabolic cost of neural information. *Nat. Neurosci.* **1**, 36–41 (1998). 10.1038/236 10195106

[113] Niven, J. E. & Laughlin, S. B. Energy limitation as a selective pressure on the evolution of sensory systems. *J. Exp. Biol.* **211**, 1792–1804 (2008). 10.1242/jeb.017574 18490395

[114] Rumelhart, D. E., Hinton, G. E. & Williams, R. J. Learning representations by back-propagating errors. *Nature* **323**, 533–536 (1986).

[115] Neftci, E. O., Mostafa, H. & Zenke, F. Surrogate gradient learning in spiking neural networks: Bringing the power of gradient-based optimization to spiking neural networks. *IEEE Signal Process. Mag.* **36**, 51–63 (2019).

[116] Zenke, F. & Ganguli, S. Superspike: supervised learning in multilayer spiking neural networks. *Neural Comput.* **30**, 1514–1541 (2018). 10.1162/neco_a_01086 PMC6118408 29652587

[117] Zenke, F. & Vogels, T. P. The remarkable robustness of surrogate gradient learning for instilling complex function in spiking neural networks. *Neural Comput.***33**, 899–925 (2021).10.1162/neco_a_0136733513328

[118] Kingma, D. P. & Ba, J. Adam: a method for stochastic optimization. Preprint at arXiv:1412.6980v9. 10.48550/arXiv.1412.6980 (2017).

[119] Rasch, M. J., Schuch, K., Logothetis, N. K. & Maass, W. Statistical comparison of spike responses to natural stimuli in monkey area V1 with simulated responses of a detailed laminar network model for a patch of V1. *J. Neurophysiol.* **105**, 757–778 (2011). 10.1152/jn.00845.2009 PMC3059174 21106898

[120] Olmos, A. & Kingdom, F. A. A biologically inspired algorithm for the recovery of shading and reflectance images. *Perception* **33**, 1463–1473 (2004). 10.1068/p5321 15729913

[121] Ruda, K., Rudzite, A. M. & Field, G. D. The functional organization of retinal ganglion cell receptive fields across light levels. Preprint at *bioRxiv* 10.1101/2022.09.15.508164 (2022).

[122] Van Der Linde, I., Rajashekar, U., Bovik, A. C. & Cormack, L. K. DOVES: a database of visual eye movements. *Spat. Vis.* **22**, 161 (2009). 10.1163/156856809787465636 19228456

[123] Van Hateren, J. H. & van der Schaaf, A. Independent component filters of natural images compared with simple cells in primary visual cortex. *Proc. R. Soc. Lond. Ser. B Biol. Sci.* **265**, 359–366 (1998). 10.1098/rspb.1998.0303 PMC1688904 9523437

[124] Storrs, K. R., Kietzmann, T. C., Walther, A., Mehrer, J. & Kriegeskorte, N. Diverse deep neural networks all predict human inferior temporal cortex well, after training and fitting. *J. Cogn. Neurosci.* **33**, 2044–2064 (2021). 10.1162/jocn_a_01755 34272948

[125] Cadena, S. A. et al. Deep convolutional models improve predictions of macaque V1 responses to natural images. *PLoS Comput. Biol.* **15**, e1006897 (2019). 10.1371/journal.pcbi.1006897 PMC6499433 31013278

[126] Mineault, P., Bakhtiari, S., Richards, B. & Pack, C. Your head is there to move you around: goal-driven models of the primate dorsal pathway. *Adv. Neural Inf. Process. Syst.* **34**, 28757–28771 (2021).

[127] Hsu, A., Borst, A. & Theunissen, F. E. Quantifying variability in neural responses and its application for the validation of model predictions. *Netw. Comput. Neural Syst.* **15**, 91–109 (2004). 15214701

[128] Taylor, L. Natural movies, Version 2 10.6084/m9.figshare.24265498 (2024).

[129] Taylor, L., Zenke, F., King, A. J. & Harper, N. S. A spiking temporal prediction model of the retina. https://github.com/webstorms/RetinalModel. Version archived on Zenodo, GitHub repository (accessed 16 July 2026) .

[130] Paszke, A. et al. PyTorch: an imperative style, high-performance deep learning library. *Adv. Neural Inf. Process. Syst.* **32**, 8026–8037 (2019).

[131] Taylor, L. DevTorch: a lightweight deep learning framework to rapidly prototype AI models. 10.5281/zenodo.11383797 (2024).

[132] Taylor, L. Brainbox: simplifying the life of a computational neuroscientist. 10.5281/zenodo.14787822 (2025).

