# Supplementary material for: Temporal prediction captures retinal spiking responses across animal species
Source: Nat Commun. 2026 Sep 8;17:10624. doi: 10.1038/s41467-026-77399-y (PMC13650136; doi:10.1038/s41467-026-77399-y)
Supplement: Supplementary file 1 [file 41467_2026_77399_MOESM1_ESM.pdf]

Dataset	Scale	CC_{norm}
Macaque movie	× 0.66	0.40
	**× 1.0**	**0.42**
	× 1.5	0.39
Macaque image	× 0.66	0.60
	**× 1.0**	**0.70**
	× 1.5	0.52
Marmoset movie	× 0.66	0.24
	× 1.0	0.26
	**× 1.5**	**0.27**
Salamander image	× 0.66	0.48
	**× 1.0**	**0.52**
	× 1.5	0.52
Mouse movie	× 0.66	0.13
	× 1.0	0.16
	**× 1.5**	**0.17**

Supplementary Table. 1: **Prediction accuracy across different scalings.** Prediction score using the non-predictive encoding retinal model across the different datasets at different spatial scales. Bold values indicate the best performing scale, which was chosen to fit the remaining predictive retinal models in each respective dataset.

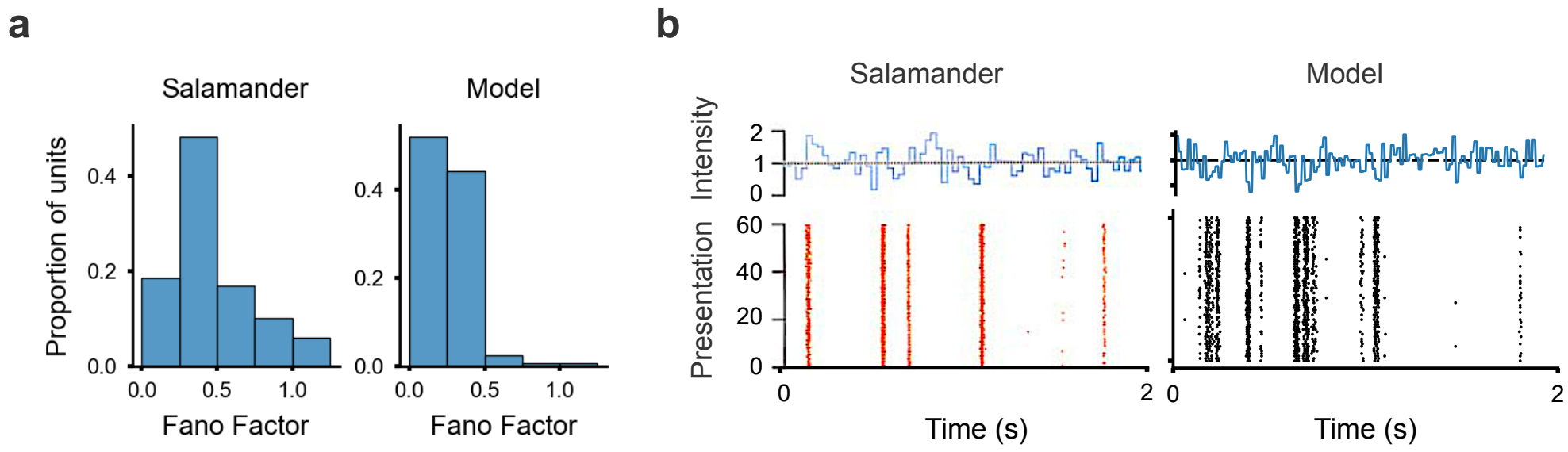

Supplementary Fig. 1: **Model response variability. a** Histogram of Fano factors in the salamander retina (data from [37]) (left) and the temporal prediction model (right) in response to the same natural images. **b** Spike raster over 60 trials in response to a uniform full-field illumination of randomly-varying intensity in the salamander retina [3] (left) and the model (right). The salamander response is reproduced from The neural code of the retina. Meister M, Berry MJ. Neuron 22:435-50. Copyright (1999), with permission from Elsevier.

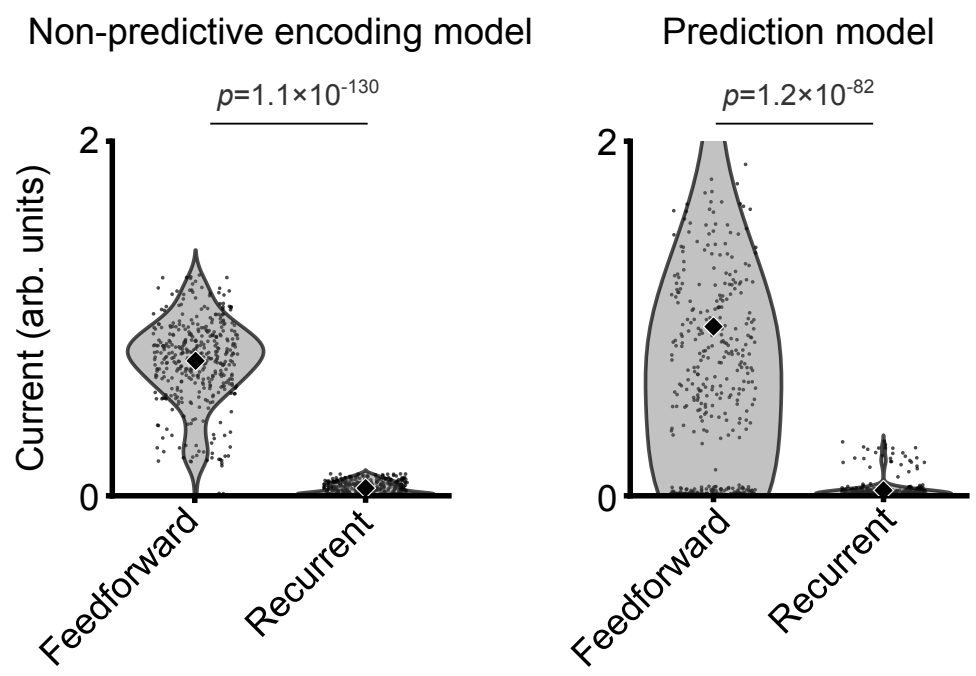

Supplementary Fig. 2: **Feedforward vs recurrent current contribution.** Violin plots of mean absolute input current across model units using the held-out test set data (significance assessed using the one-sided Mann-Whitney U test). Black diamonds show the means, each point is a model unit, and the violins show the distributions.

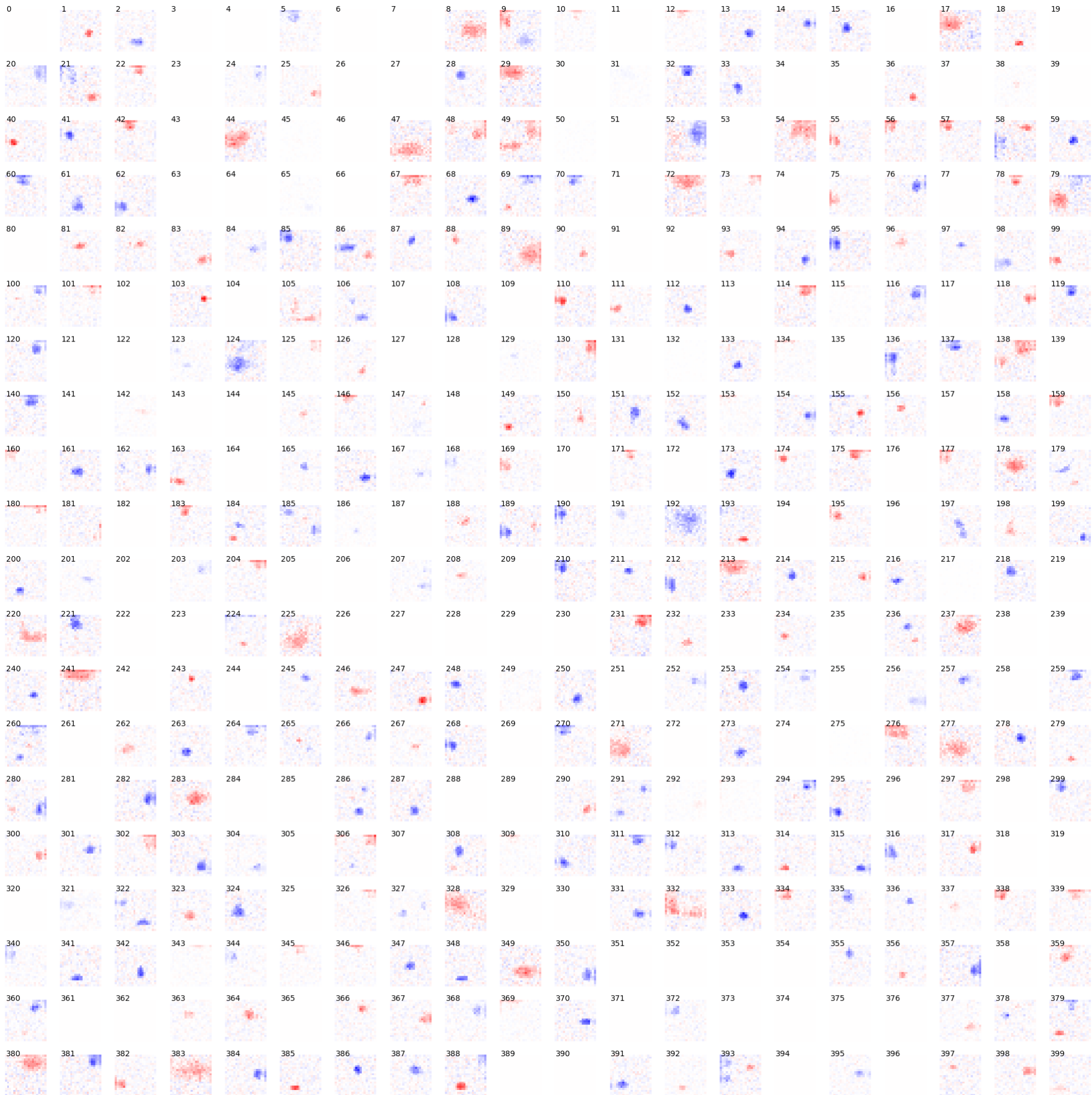

Supplementary Fig. 3: **Temporal prediction model spatial RFs.** Each square corresponds to a model unit's spatial RF, i.e., a slice through the spatiotemporal RF at the time point with the largest power (sum of squared weights over space). Colormap as Fig. 3b, red is positive weight, blue is negative weight. Blank squares denote dead units.

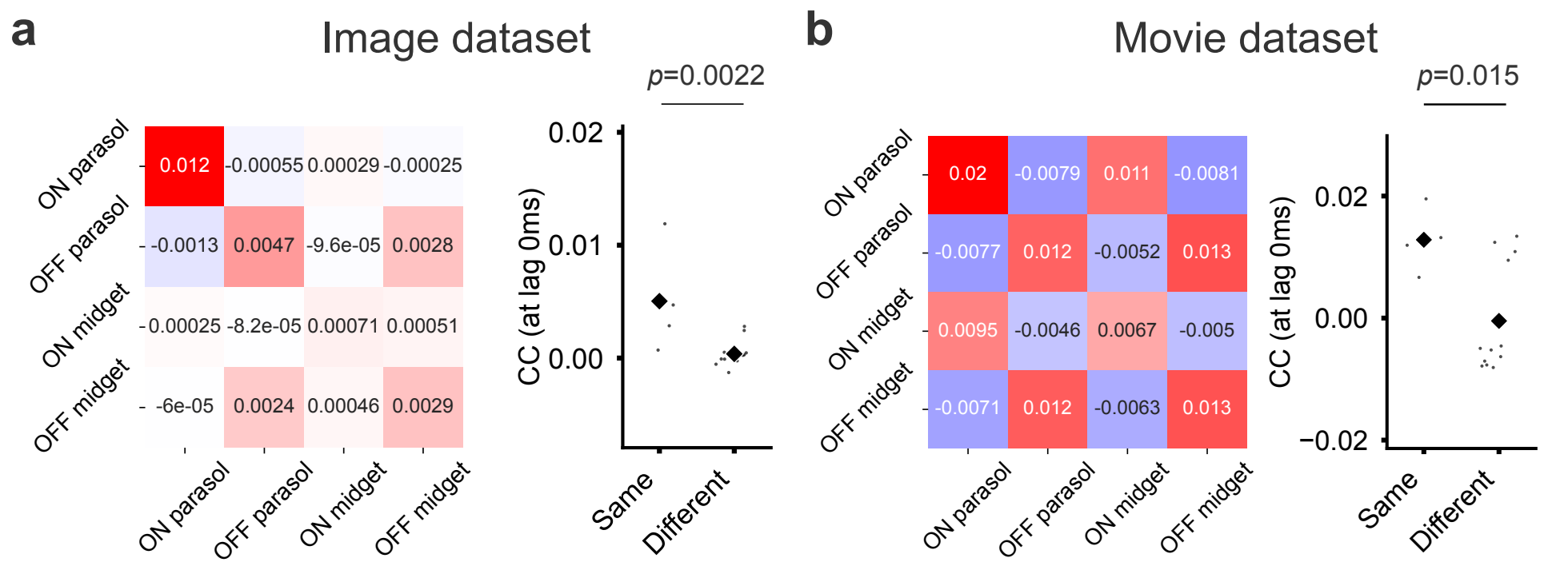

Supplementary Fig. 4: **Response correlations between RGC-like functional classes of the prediction model units.** **a** Left: response correlations between the same and different unit functional classes of the prediction model for the image dataset. Right: Comparison of within-class and different-class correlation coefficients. Black diamonds show the means, each point is a correlation coefficient. Statistical significance was assessed using the Mann-Whitney U test. All correlations are taken from the cross-correlogram at lag zero. **b** Same as in **a**, but using the response correlations obtained for the movie dataset.

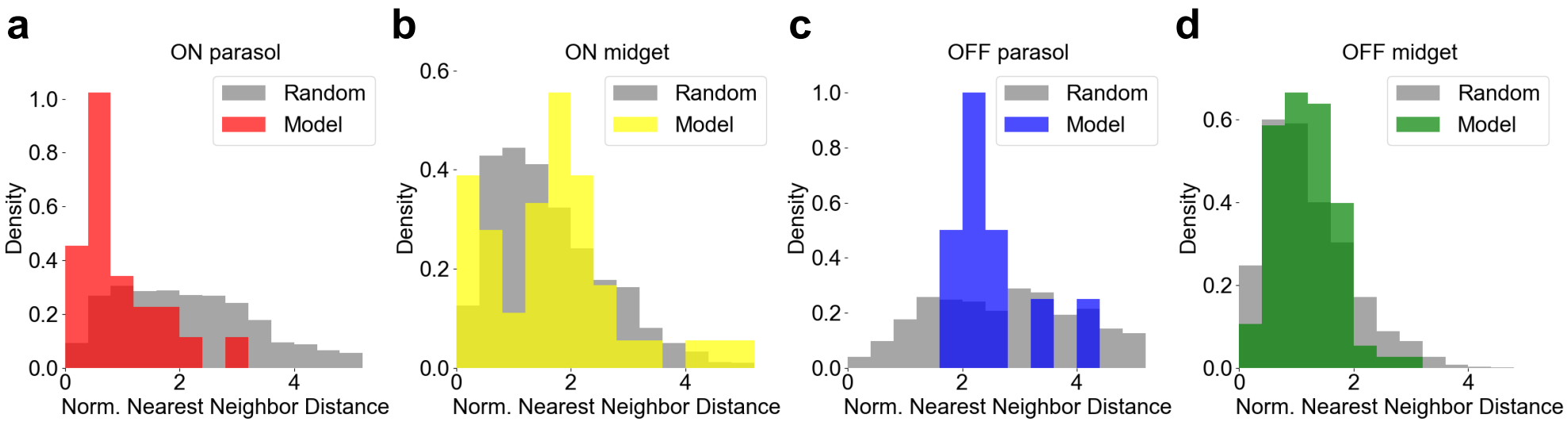

Supplementary Fig. 5: **Mosaic tiling quantification.** We assessed the tiling overlap of the spatial receptive fields of the prediction model units using the mosaic tiling measure known as the normalized nearest neighbor distance [49]; a low value means more overlap and a high value means less overlap. In the retina, the different cell types each show a peak in the distribution of this measure below ∼2, with clear space between the peak and 0, and have measure distributions that differ from that of a RGC population with randomly drawn locations [49]. This figure shows the distributions of the measure from the prediction model for **a** ON-type parasol-like units, **b** ON-type midget-like units, **c** OFF-type parasol-like units and **d** OFF-type midget-like units. The OFF-type midget-like units show a distribution resembling that seen in real RGCs. The rest of the units either overlap more (ON parasol, ON midget) or less (OFF parasol) than the corresponding cell types in the retina. However, consistent with the biology, all the cell-type-like model unit populations, with the exception of the OFF-parasol-like units (which are a very small population) are tiled significantly differently compared to the distributions obtained by drawing their positions randomly from that population (random position distribution in gray, made using 1000 iterations), as confirmed with a KS test between model distribution and random position distribution (ON parasol, $p = 1,7 \times 10^{-4}$; ON midget, $p = 9.6 \times 10^{-3}$, OFF parasol, $p = 0.11$; OFF midget, $p = 1.1 \times 10^{-17}$).

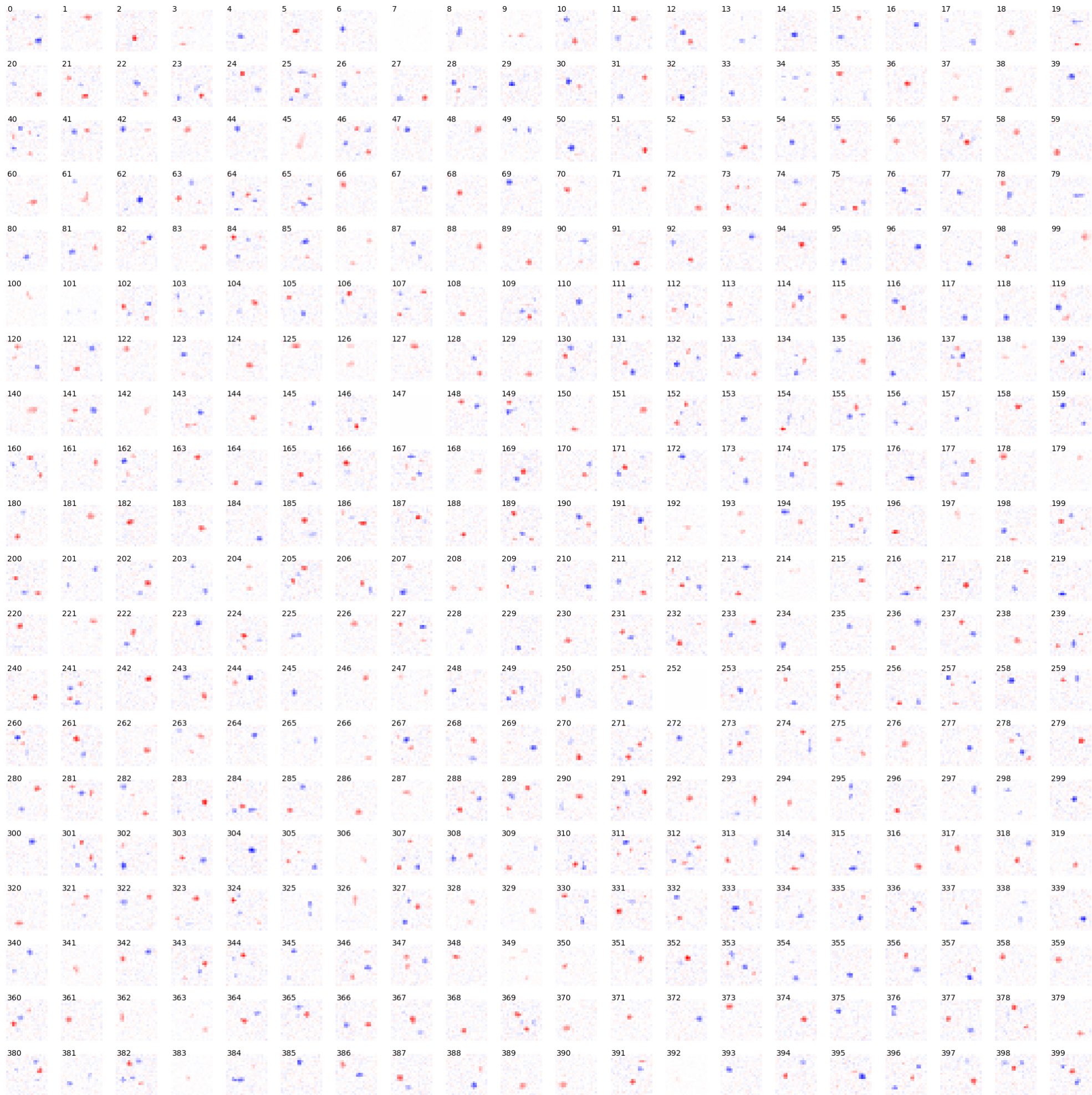

Supplementary Fig. 6: **Non-predictive encoding model spatial RFs.** Every square corresponds to each unit's spatial RF. Colormap as Fig. 3b, red is positive weight, blue is negative weight.

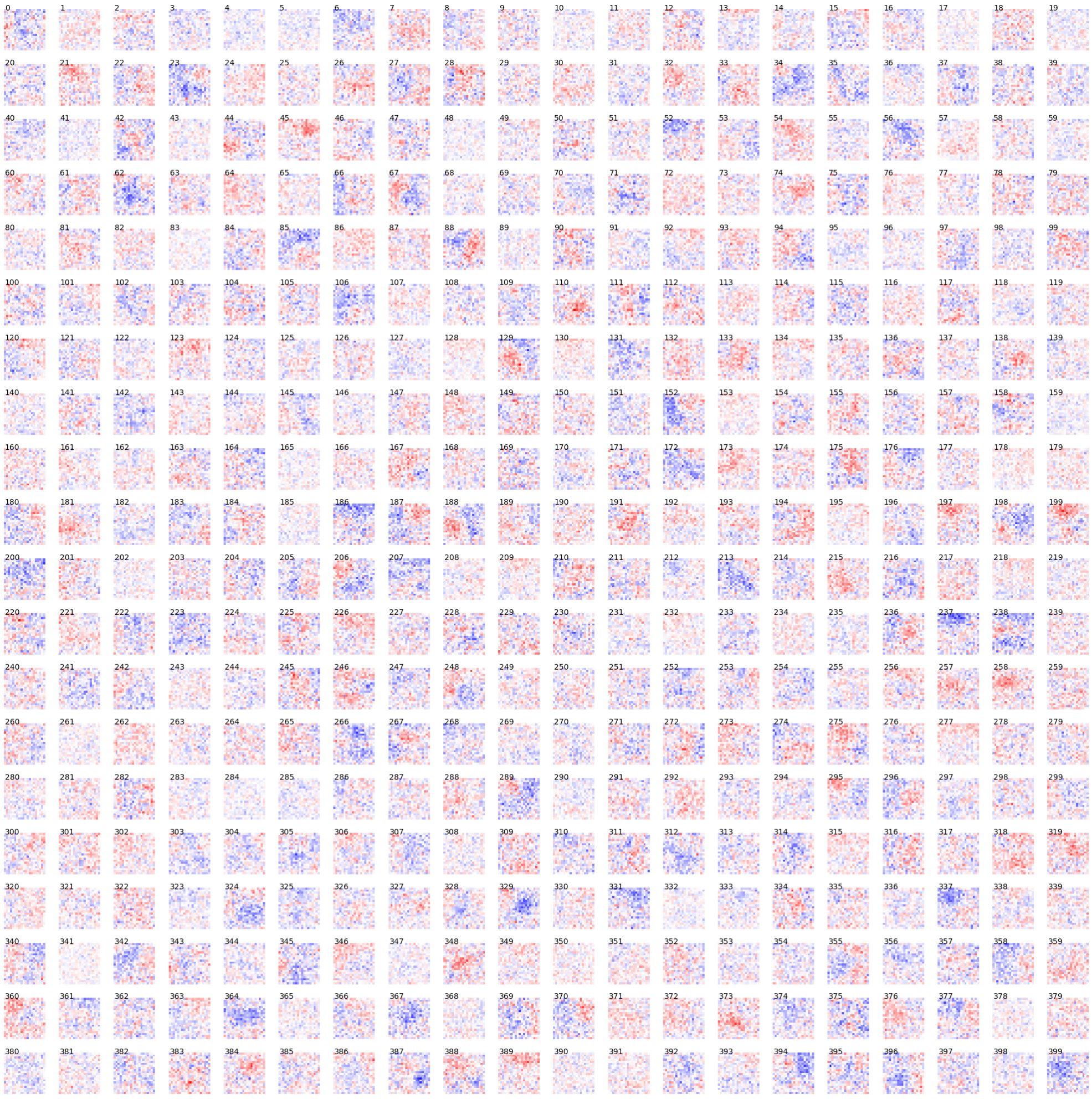

Supplementary Fig. 7: **Temporal prediction model spatial RFs trained without metabolic regularization.** Every square corresponds to each unit's spatial RF. Colormap as Fig. 3b, red is positive weight, blue is negative weight.

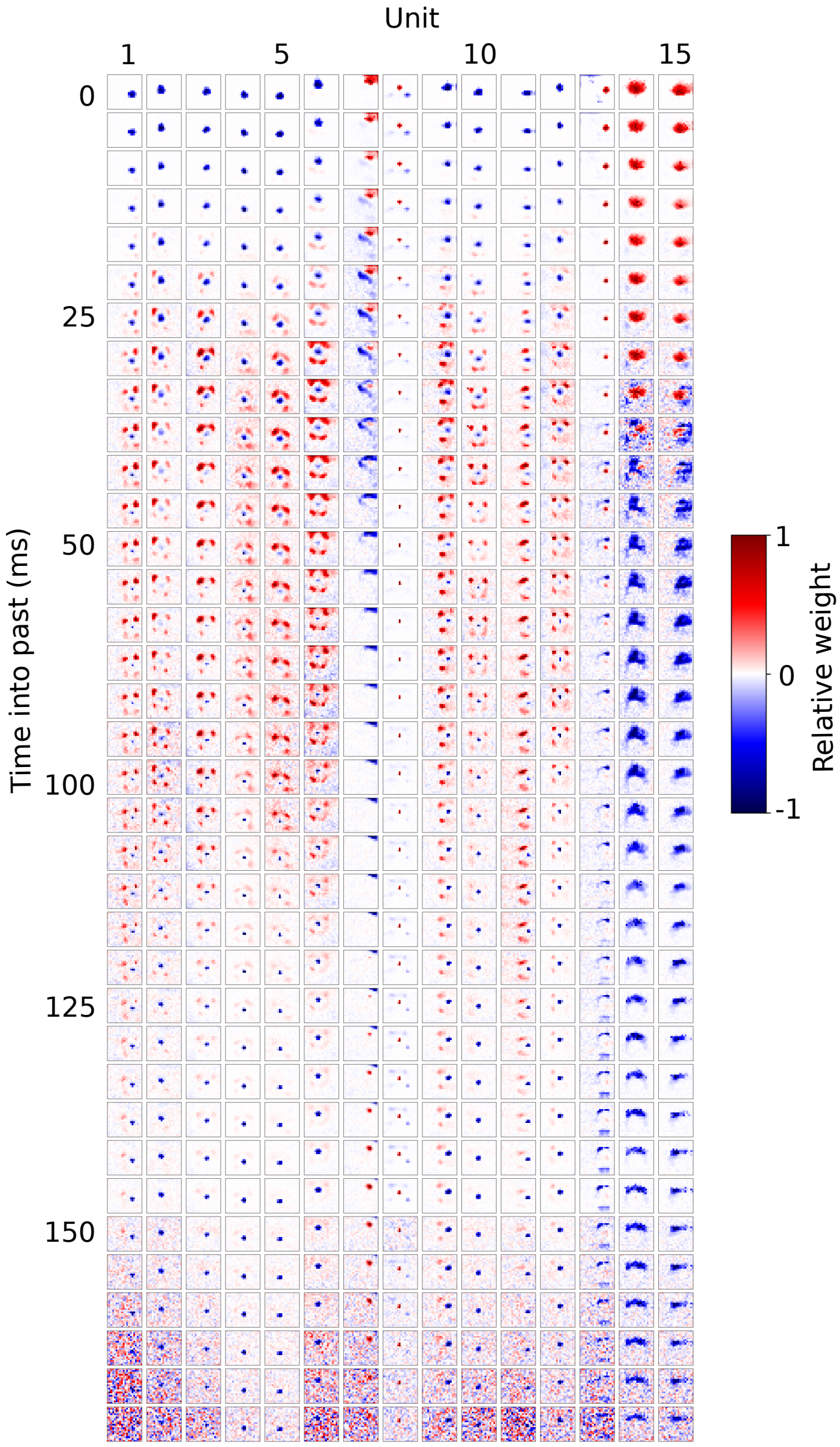

Supplementary Fig. 8: **Example spatiotemporal receptive fields of the prediction model showing lagging surrounds**. The receptive fields were measured using 500 times more noise snippets of twice the length than for the other figures. Also, to display the RF, each time point was independently normalized and a more sensitive colormap was used.

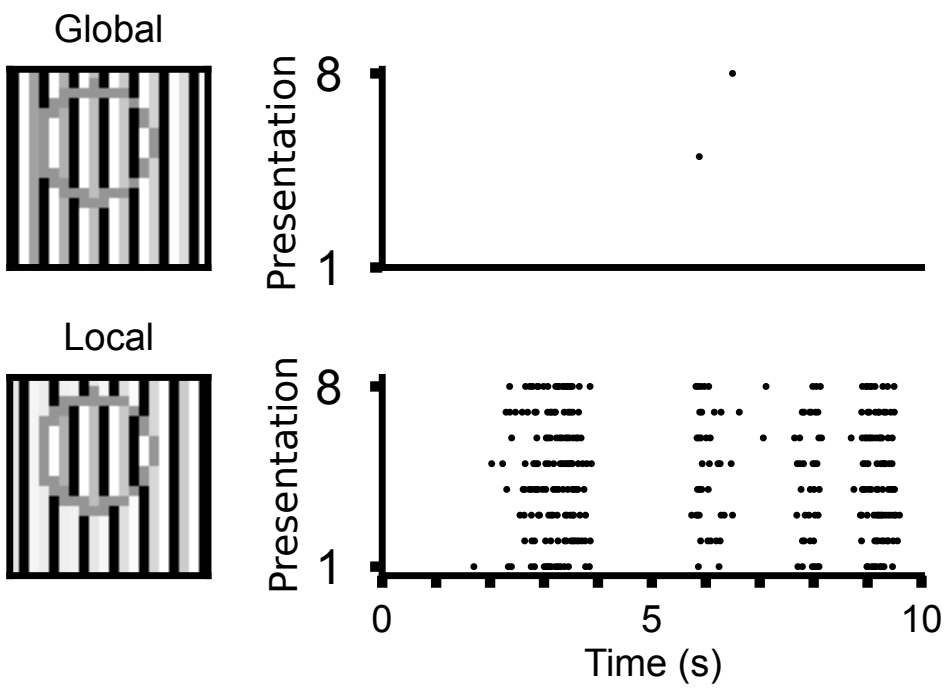

Supplementary Fig. 9: **Object-motion sensitivity** Top: Raster plot showing the response of an example temporal prediction model unit, with coherent jitter between the center and surround (global). Bottom: The same unit, with differential (independent) jitter between the center and surround (local). Note the stronger response to local than global motion.

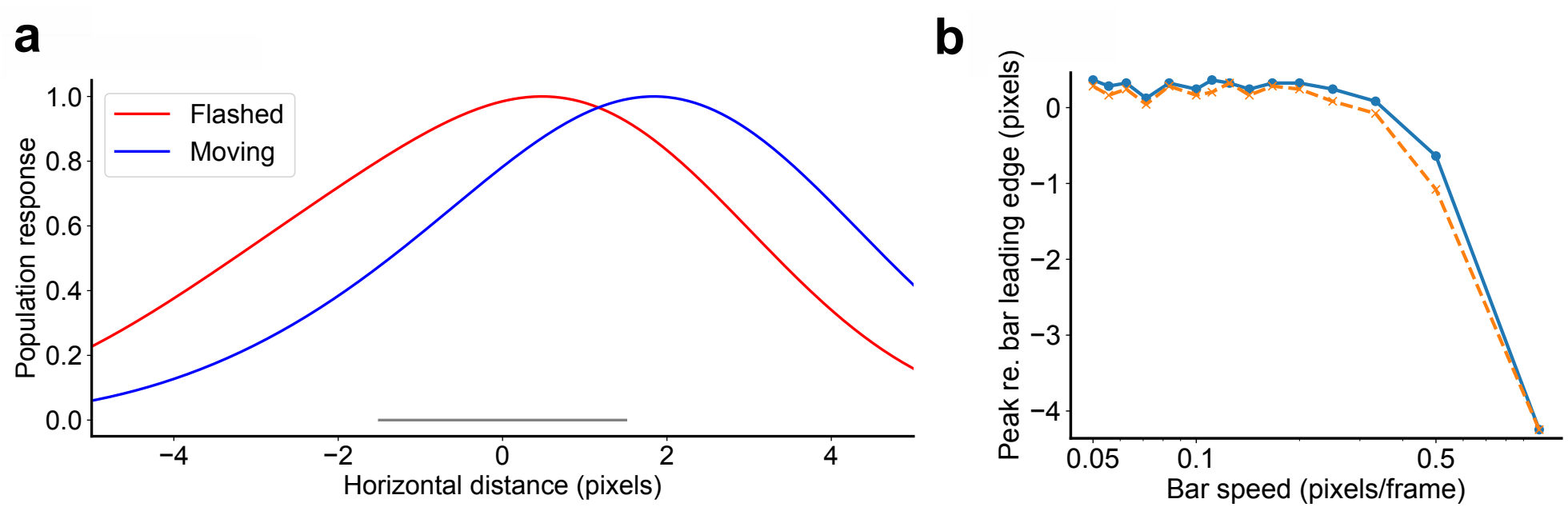

Supplementary Fig. 10: **Anticipation-like population responses**. **a** The population response (blue line), i.e., the sum of firing rates over units, of the OFF-type parasol-like units of the prediction model to a rightward moving vertical bar. This is plotted as a function of the horizontal spatial position of the units' receptive fields relative to a reference horizontal position, at the time when the bar first arrives at that reference position. The horizontal gray line shows the width of the bar at the reference position. Also plotted for the same units is the population response (red line) at stimulus onset time to the same vertical bar, but in this case the bar is not moving but is flashed on at the reference position. These population responses are the mean of the population responses for 4 different reference positions. The reference positions were at 11, 12, 13 or 14 pixels from the left of the frame, to allow sufficient time for any anticipation of the moving bar to develop. The population responses were then smoothed over space using a Gaussian with a 2 pixel standard deviation and normalized by their peak. **b** The position of the peak of the population response to the moving bar relative to the leading edge of the bar, as a function of the bar speed, for the prediction model (cyan solid line) and the prediction model with the recurrent connections ablated (orange dashed line).

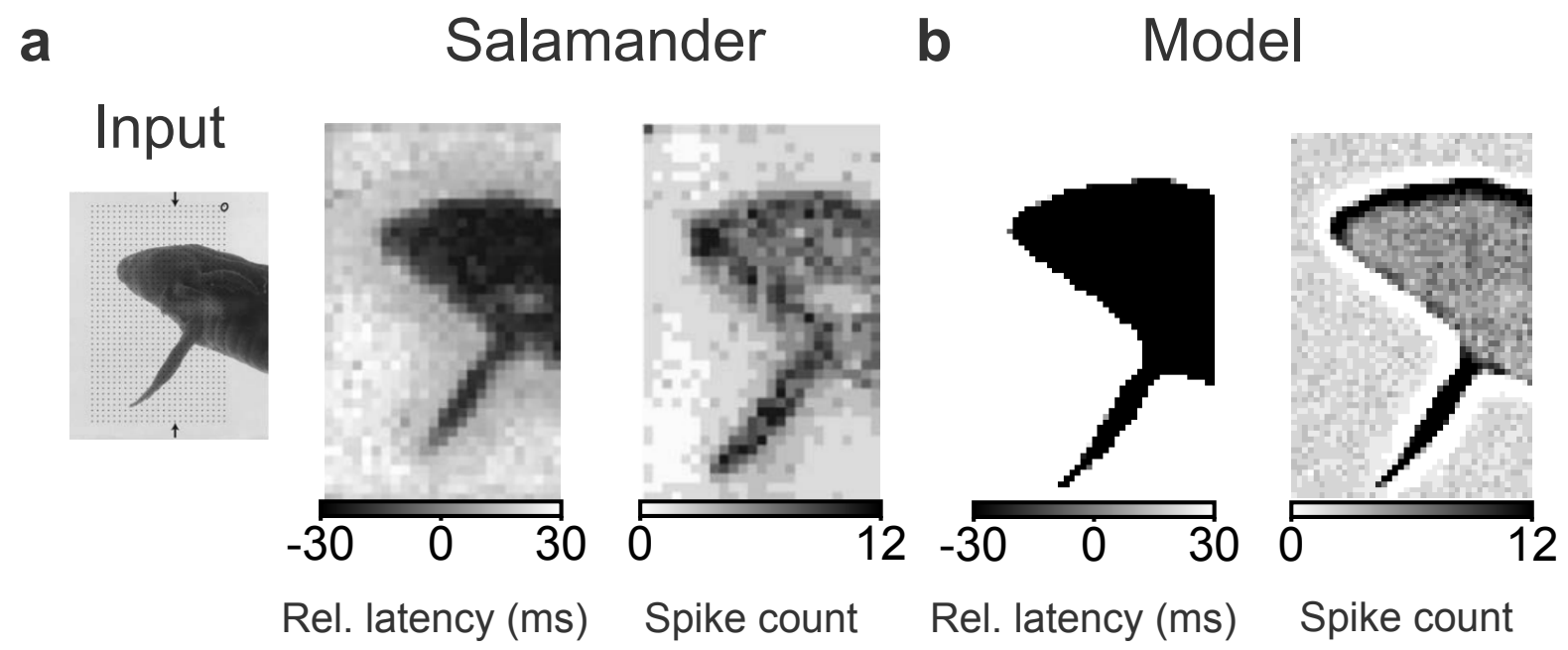

Supplementary Fig. 11: **Reconstruction of an image using a single OFF-type neuron or model unit. a** Reconstruction of an input image using either the relative spike latencies (left) or spike counts (right) recorded from a single OFF-type neuron in the salamander retina [23]. **b** Same for a single OFF-type model unit. Note the over emphasis of edges with the spike count reconstruction in both the experimental data and the model. The image and salamander figures in **a** are reproduced with permission from Gollisch and Meister, Science, doi 10.1126/science.1149639 [2008], AAAS.

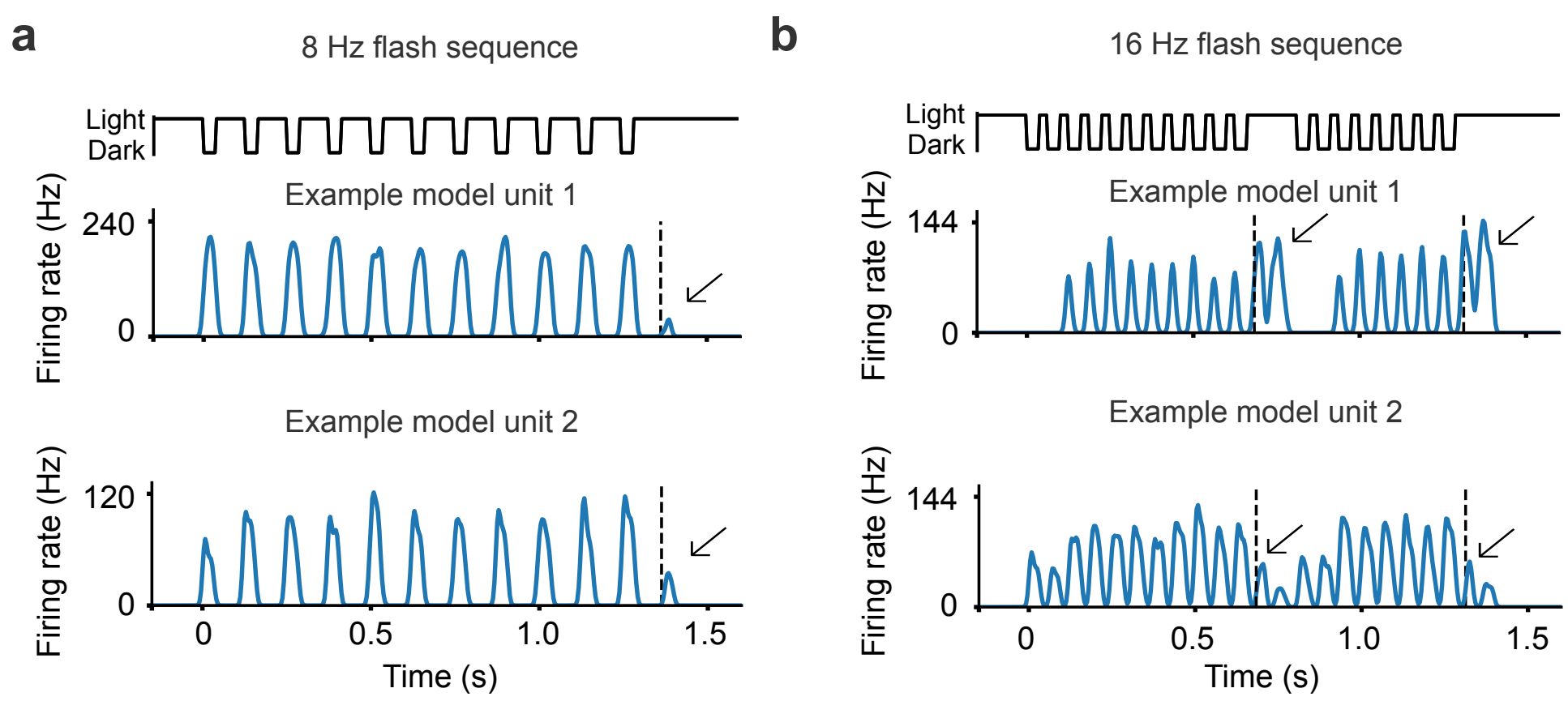

Supplementary Fig. 12: **Omission stimulus responses. a** Example model unit responses to a sequence of dark-flashes occurring at 8 Hz. **b** Example model unit responses to a sequence flashing at 16Hz with a dark-flash omission in the middle of the sequence. The dashed line marks the time when the dark-flash was omitted, with the arrow indicating the omitted-stimulus response.

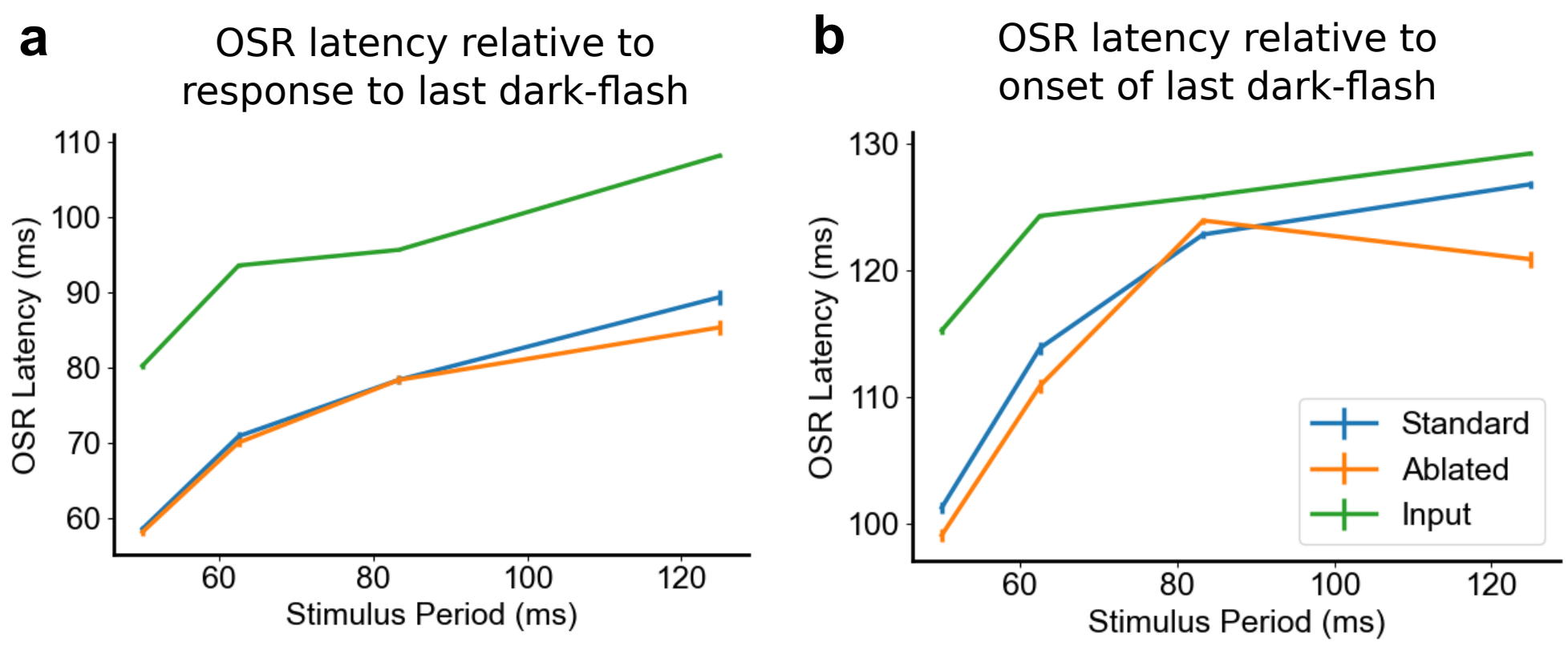

Supplementary Fig. 13: **Dependence of OSR latency on the flash sequence period.** Plots show average latency over all temporal prediction model units in the population that gave an omission response for that stimulus rate. Blue is the latency measured using the firing rate in the standard model, orange is the latency measured using the firing rate in the recurrency ablated model, and green is the value measured in the feedforward input current. **a** OSR latency measured from the peak of the response to the last dark-flash to the peak of the OSR. **b** OSR latency measured from the onset of the last dark-flash to the peak of the OSR. The error bars show the standard error of the mean across model units.

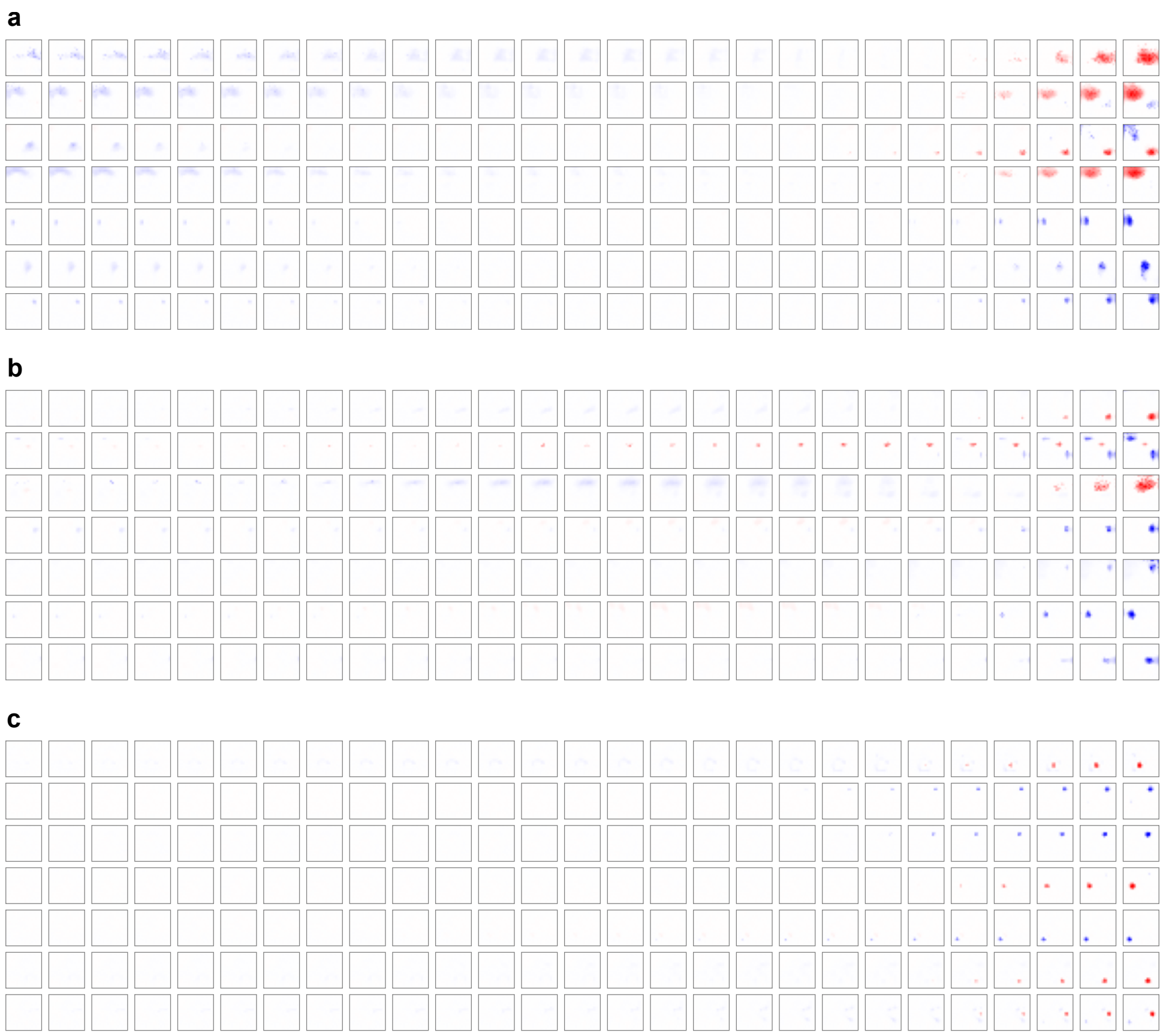

Supplementary Fig. 14: **Input weights of model units that either did or did not produce OSRs. a** Each row depicts the input weights, W, of a prediction model unit that produced an OSR. **b** Each row depicts the input weights of a prediction model unit that did not produce an OSR. **c** Each row depicts the input weights of a unit from the non-predictive encoding model, for which no units produced OSRs.

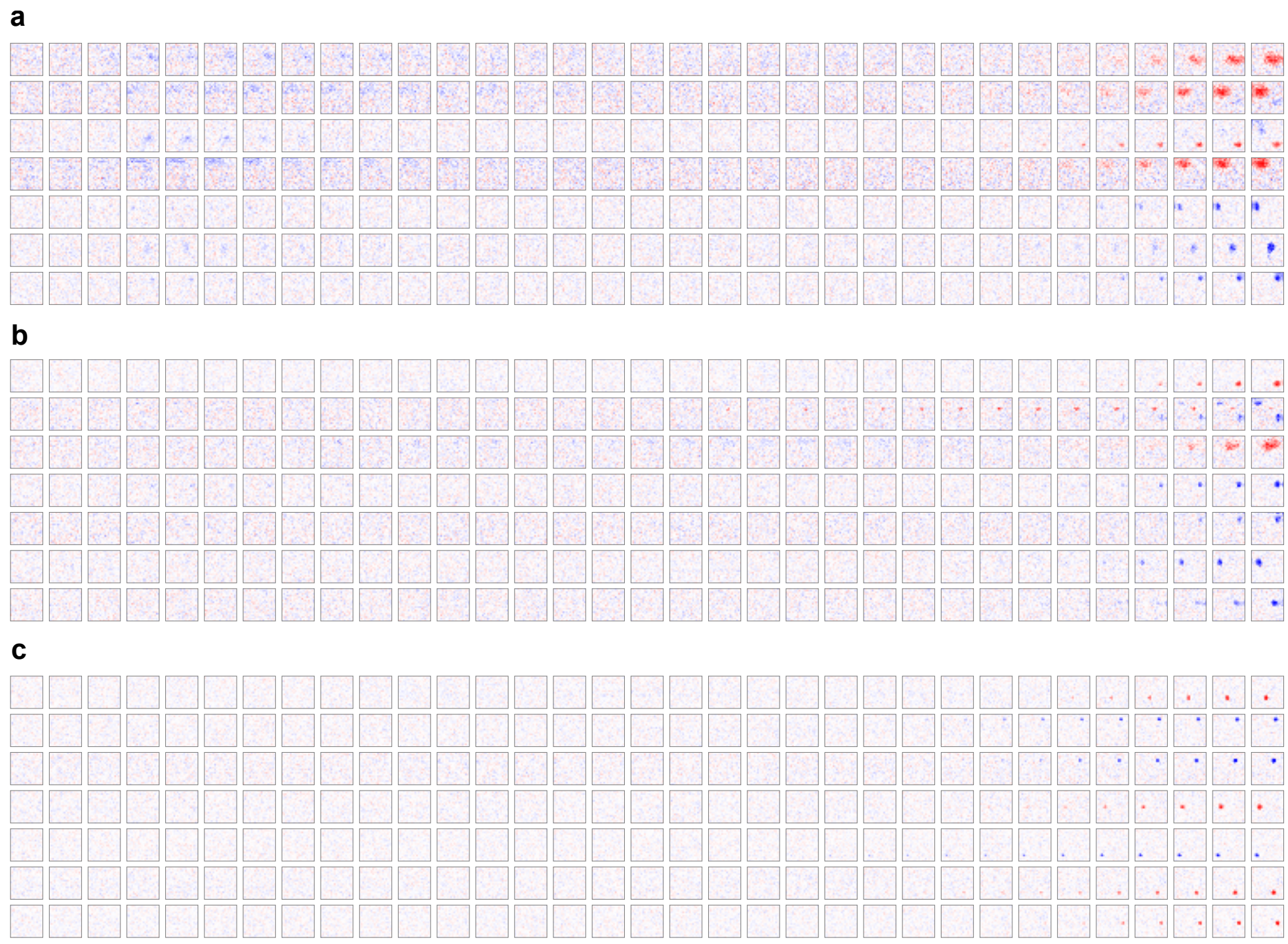

Supplementary Fig. 15: **Spatiotemporal RFs of model units that either did or did not produce OSRs. a** Each row depicts the spatiotemporal RF of a prediction model unit that produced an OSR. **b** Each row depicts the spatiotemporal RF of a prediction model unit that did not produce an OSR. **c** Each row depicts the spatiotemporal RF of a unit from the non-predictive encoding model, for which no units produced OSRs.

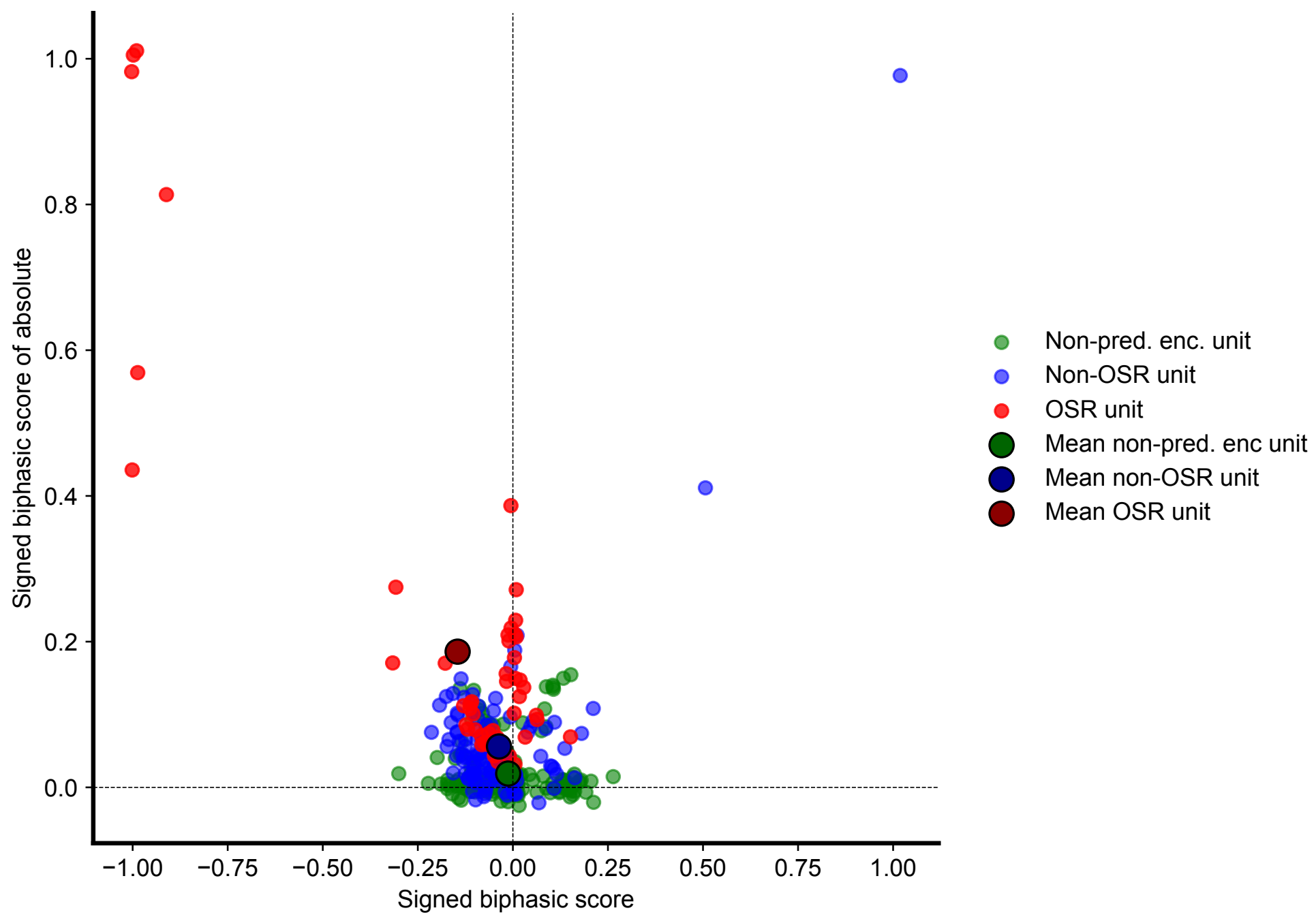

Supplementary Fig. 16: **Measurement of the bi-phasic character of the input weights.** First, we averaged the input weights over space for each latency, giving an array of 30 values extending into the past. The first, most recent value typically has the largest magnitude. If this first value (z_1) was positive, we found the minimum value of the array (z_{min}), and measured the highest value to the past of it ($z_{rebound}$). We then measured $(z_{rebound} - z_{min})/(z_1 - z_{min})$. If this first value ($z_1$) was negative, we found the maximum value of the array (z_{max}), and measured the lowest value to the past of it ($z_{rebound}$). We then measured $(z_{rebound} - z_{max})/(z_1 - z_{max})$. The measure was clipped between -1 and 1. Hence, a measure above -1 and below 0 indicates an array that dips partially after a rise, 0 indicates a monotonic array, and above 0 and below 1 indicates an array that rises partially after a dip, with the magnitude giving the degree of return. We calculated this measure for each OSR unit and non-OSR unit of the prediction model, defining an OSR unit as a unit that showed an OSR for at least one sequence rate. We also calculated this for the non-predictive encoding model units. This measure provides the x-axis of the plot. The y-axis is provided by this measure applied to the absolute values of the array, to measure dips in magnitude irrespective of sign. Units in the left half typically show negative weights that dip and rise slightly in magnitude, whereas units in the right half typically show positive weights that do the same, and units with a zero x-measure but non-zero y-measure are monotonic but flip sign into the past. Note that the OSR units typically have either negative dip-to-rise weights or positive-to-negative weights, whereas the non-OSR and non-predictive encoding model weights are closer to the origin, indicating that they are less biphasic.
