# Supplementary material for: Temporal prediction captures retinal spiking responses across animal species
Source: Nat Commun. 2026 Sep 8;17:10624. doi: 10.1038/s41467-026-77399-y (PMC13650136; doi:10.1038/s41467-026-77399-y)
Supplement: Supplementary file 2 [file 41467_2026_77399_MOESM2_ESM.pdf]

nature portfolio

Corresponding author(s): Luke Taylor, Andrew J King, Nicol S Harper

Last updated by author(s): Apr 24, 2026

Reporting Summary

Nature Portfolio wishes to improve the reproducibility of the work that we publish. This form provides structure for consistency and transparency in reporting. For further information on Nature Portfolio policies, see our Editorial Policies and the Editorial Policy Checklist.

Statistics

For all statistical analyses, confirm that the following items are present in the figure legend, table legend, main text, or Methods section.

n/a	Confirmed	
	☒	The exact sample size (*n*) for each experimental group/condition, given as a discrete number and unit of measurement
☒		A statement on whether measurements were taken from distinct samples or whether the same sample was measured repeatedly
	☒	The statistical test(s) used AND whether they are one- or two-sided<br>*Only common tests should be described solely by name; describe more complex techniques in the Methods section.*
☒		A description of all covariates tested
☒		A description of any assumptions or corrections, such as tests of normality and adjustment for multiple comparisons
	☒	A full description of the statistical parameters including central tendency (e.g. means) or other basic estimates (e.g. regression coefficient) AND variation (e.g. standard deviation) or associated estimates of uncertainty (e.g. confidence intervals)
	☒	For null hypothesis testing, the test statistic (e.g. *F*, *t*, *r*) with confidence intervals, effect sizes, degrees of freedom and *P* value noted<br>*Give P values as exact values whenever suitable.*
☒		For Bayesian analysis, information on the choice of priors and Markov chain Monte Carlo settings
	☒	For hierarchical and complex designs, identification of the appropriate level for tests and full reporting of outcomes
	☒	Estimates of effect sizes (e.g. Cohen's *d*, Pearson's *r*), indicating how they were calculated

Our web collection on statistics for biologists contains articles on many of the points above.

Software and code

Policy information about availability of computer code

Data collection	Computational study, not relevant
Data analysis	The code of the spiking retinal model and the code for reproducing the results can be found at [1]. The code was mostly written in Python (3:10) using Jupyter Notebook (6.0.3), with a very small amount of Matlab (R2025b). We used PyTorch (2.1.2) [2] and DevTorch (0.0.5) [3] for implementing the models and BrainBox (0.0.9) [4] for analyzing the spike statistics, receptive fields and tuning properties. The code was run on an Ubuntu desktop using an NVIDIA GeForce RTX 3090 GPU.<br><br>1. Taylor, L., Zenke, F., King, A. J. & Harper, N. S. Retinalmodel. https://github.com/webstorms/RetinalModel (2026). GitHub repository, accessed 16 July 2026.<br>2. Paszke, A. et al. Pytorch: An imperative style, high-performance deep learning library. Advances in neural information processing systems 32 (2019)<br>3. Taylor, L. Devtorch: A lightweight deep learning framework to rapidly prototype ai models (2024). URL https://doi.org/10.5281/ zenodo.11383797.<br>4. Taylor, L. Brainbox: Simplifying the life of a computational neuroscientist. (2025). URL https://doi.org/10.5281/zenodo.14787822

For manuscripts utilizing custom algorithms or software that are central to the research but not yet described in published literature, software must be made available to editors and reviewers. We strongly encourage code deposition in a community repository (e.g. GitHub). See the Nature Portfolio guidelines for submitting code & software for further information.

Data

Policy information about availability of data

All manuscripts must include a data availability statement. This statement should provide the following information, where applicable:

- Accession codes, unique identifiers, or web links for publicly available datasets
- A description of any restrictions on data availability
- For clinical datasets or third party data, please ensure that the statement adheres to our policy

The natural movie training data recorded for this study have been deposited in the Figshare repository [1]. Pre-trained spiking models can be found at [2]. We did not perform any of the retinal recordings and had no role in the design of these studies. Inspect the data availability sections in the relevant papers cited in this manuscript to access the retinal data. We specify in the section of the Methods in which these studies are described whether the retinal recording data were obtained from one or both sexes, as stated in the original publications.

1. Taylor, L. Natural movies (2024). URL https://doi.org/10.6084/m9.figshare.
24265498. Version 2.
2. Taylor, L., Zenke, F., King, A. J. & Harper, N. S. Retinalmodel. https://github.com/
webstorms/RetinalModel (2026). GitHub repository, accessed 16 July 2026.

Research involving human participants, their data, or biological material

Policy information about studies with human participants or human data. See also policy information about sex, gender (identity/presentation), and sexual orientation and race, ethnicity and racism.

Reporting on sex and gender	Computational study, not relevant
Reporting on race, ethnicity, or other socially relevant groupings	Computational study, not relevant
Population characteristics	Computational study, not relevant
Recruitment	Computational study, not relevant
Ethics oversight	Computational study, not relevant

Note that full information on the approval of the study protocol must also be provided in the manuscript.

Field-specific reporting

Please select the one below that is the best fit for your research. If you are not sure, read the appropriate sections before making your selection.

☒ Life sciences ☐ Behavioural & social sciences ☐ Ecological, evolutionary & environmental sciences

For a reference copy of the document with all sections, see nature.com/documents/nr-reporting-summary-flat.pdf

Life sciences study design

All studies must disclose on these points even when the disclosure is negative.

Sample size	No formal sample size calculation was performed beforehand, as this is a computational study that used publicly available datasets, and sample sizes were determined by data availability.
Data exclusions	The following exclusions were applied: inactive units and units that did not respond to the held-out test set of natural clips were excluded from all analyses (113 of 400 units excluded). For CV(ISI) analyses, values were excluded when fewer than three spikes were present for a given stimulus presentation. For receptive-field analyses, units were excluded unless they had a sufficiently good 2D Gaussian fit to the spatial RF (correlation coefficient > 0.7 and σX and σY > 0.5 pixels), leaving 173 of 287 active units. For neural prediction analyses, the most responsive neurons were selected using CC_{max} > 0.93 for the marmoset and mouse datasets. These exclusion criteria were not formally preregistered, but were applied as standard quality-control thresholds.
Replication	Reproducibility was supported by using held-out datasets where predictive evaluation was required: this includes the spike-statistics comparisons in Fig. 2 and Supplementary Fig. 2, the noise-corrupted image decoding analysis in Fig. 5a–b, and the cross-species neural-prediction analysis in Fig. 6. Other analyses, such as the receptive-field analyses in Fig. 3 and Supplementary Figs. 3–8, and the motion-tuning and omitted-stimulus analyses in Fig. 4, Fig. 5c, and Supplementary Figs. 9–10, 12-13 and 15, used long input streams and/or repeated stimulus presentations to estimate relevant measures. In Fig. 6 we also compare against different datasets from multiple species to ensure robustness of results.
Randomization	Not applicable. This is a computational study that did not involve allocation to experimental groups.
Blinding	Blinding and group allocation not relevant to this computational study.

Reporting for specific materials, systems and methods

We require information from authors about some types of materials, experimental systems and methods used in many studies. Here, indicate whether each material, system or method listed is relevant to your study. If you are not sure if a list item applies to your research, read the appropriate section before selecting a response.

Materials & experimental systems

n/a	Involved in the study
☒	☐ Antibodies
☒	☐ Eukaryotic cell lines
☒	☐ Palaeontology and archaeology
☒	☐ Animals and other organisms
☒	☐ Clinical data
☒	☐ Dual use research of concern
☒	☐ Plants

Methods

n/a	Involved in the study
☒	☐ ChIP-seq
☒	☐ Flow cytometry
☒	☐ MRI-based neuroimaging

Plants

Seed stocks

Report on the source of all seed stocks or other plant material used. If applicable, state the seed stock centre and catalogue number. If plant specimens were collected from the field, describe the collection location, date and sampling procedures.

Novel plant genotypes

Describe the methods by which all novel plant genotypes were produced. This includes those generated by transgenic approaches, gene editing, chemical/radiation-based mutagenesis and hybridization. For transgenic lines, describe the transformation method, the number of independent lines analyzed and the generation upon which experiments were performed. For gene-edited lines, describe the editor used, the endogenous sequence targeted for editing, the targeting guide RNA sequence (if applicable) and how the editor was applied.

Authentication

Describe any authentication procedures for each seed stock used or novel genotype generated. Describe any experiments used to assess the effect of a mutation and, where applicable, how potential secondary effects (e.g. second site T-DNA insertions, mosiacism, off-target gene editing) were examined.
