# Supplementary material for: Temporal prediction captures retinal spiking responses across animal species
Source: Nat Commun. 2026 Sep 8;17:10624. doi: 10.1038/s41467-026-77399-y (PMC13650136; doi:10.1038/s41467-026-77399-y)
Supplement: Supplementary file 3 [file 41467_2026_77399_MOESM3_ESM.pdf]

nature portfolio

Peer Review File

Temporal prediction captures retinal spiking responses across animal species

Corresponding Author: Dr Luke Taylor

This file contains all reviewer reports in order by version, followed by all author rebuttals in order by version.

Version 0:

Reviewer comments:

Reviewer #1

(Remarks to the Author)
The manuscript by Taylor and colleagues explores a modeling framework for the retina based on integrate and fire model of retinal ganglion cell activity. The model yields receptive field mosaics of major retinal ganglion cell types encoding stimulus motifs, across different species. The model captures neural responses more reliably when trained to predict future events in a dynamic natural world.

The scientific question is important and deserves inquiry. The authors did an excellent job at developing the model and implementing it to test a fundamental principle: predictive coding that the retina might exploit for processing natural stimuli. The effort of the authors and the premise of the work, both deserve merit. However, the disconnect between the underlying assumptions of the model and the biological circuit, and disparities between the model output and empirical data in the literature, weaken the study.

Major comments:
1. The argument presented in the manuscript for including recurrent connections between ganglion cells is rather weak. Recurrent signaling is present in the retina, but not extensively. There are a few ganglion cell types are homotypically coupled, ipRGCs provide recurrent connections, others are coupled with specific amacrine cell types. While these connections can result in recurrent signaling, it is only a small number of ganglion cell types (at least in the mouse retina). Most of the ganglion cell types are still driven by glutamatergic signals from bipolar cells. The authors state that they preclude gap-junctions in their model of recurrent activity. Then what do these recurrent connections represent? And if recurrent connections do represent coupled activity, then shouldn't the model better predict responses of those ganglion cell types that are actually coupled?
2. Since the noise term in Eq.2 is folded into the current term that sets the membrane voltage of ganglion cells, it is reasonable to surmise that this noise term is pre-spike, suggesting its origin is from upstream circuits. Photoreceptor noise (approximately Poisson-like) and stochastic vesicle release at the bipolar synapse are the major sources of this noise (see Weber etal. 2021). Maybe I am missing something but is there an explanation for why the noise should be Gaussian instead of Poisson? Full-field illumination (line 141) for a test of noise also doesn't seem the right approach because the nonlinearity at the bipolar synapse can be significantly different for patterned stimulus.
3. There are ~45 ganglion cell types in the mouse retina. The authors show only one or two cell types in mouse that is predicted by the model. Why do the authors not find more cell types? Is it because of limited data, or is it because the model does not incorporate presynaptic nonlinearities, which are shaped by bipolar and amacrine cells to support feature detection?
4. The authors use spike rate and CV for comparing model responses with responses of actual neurons. But these metrics are inadequate in capturing the temporal dynamics of neurons driven by correlations in the natural stimuli. The authors should consider including alternative metrics such as cross-correlations or synchrony or spike train distance for cross-validating the results.
5. Responses of model units appear strongly correlated in time (Figure 2 a, b) much more than actual neurons. I wonder if this is an artifact of the recurrent term since that can force a large population of cells to fire synchronously to different phases of the stimulus. If that is not the case, then do those correlated cells belong to the same type?
6. Does the model yield receptive field mosaics of other major ganglion cell types in salamander and mouse? If it does, then the authors should show the results at least for a few other cell types in a separate figure or as a supplement.
7. Why does the model not produce a RF surround? Studies by Karamanlis-Gollisch, and others, suggest that not

accounting for nonlinearities or subunits in bipolar cells may be a factor. The authors should provide an explanation, or at the minimum speculate in Discussion. Also, the temporal RFs of model units are ~4 times faster than those estimated from actual neurons (Fig. 3b). The authors should clarify if this was expected or if it is a shortcoming of the model.
8. Is the predictive model (line 430) supposed to predict the retinal output to natural images, or predict responses of V1 neurons from retinal output? The authors refer the Cadena paper which focused on predicting V1 neuron responses, which has a different design and different underlying assumptions that a CNN based model that predicts ganglion cell responses from natural images/movies. For example, filters emulating RFs in the retina are not gabor like and V1 response is influenced strongly by recurrent and feedback signals unlike the retina. How are these differences reconciled?
9. To test OSR, the flash needs to be absent in a sequence of period flashes. The stimulus trace in Extended Data Fig. 2 shows something different, in that the flash is not omitted, rather the absence of flash is omitted. The frequency (16hz==62.5ms period) also does not correlate with what is described in methods: 80ms duration following termination of flash. The authors should explain this discrepancy, and/or retest the model output with the correct stimulus.

Minor comments:
1. Line 661: The authors state that majority of amacrine cells use spikes. This is not true. Most of the amacrine cells respond through graded potentials (there are a few displaced amacrine cell types that do spike). The ‘spikes’ in the references are calcium spikes, which can result in action potentials but not always.
2. The phrases ‘predictive model’, ‘predicting neural responses’, ‘efficient prediction’, ‘optimized for prediction’ have been used interchangeably across the manuscript. These phrases have different meanings, and the authors should carefully consider how these phrases are being used and where.
3. P-values needed in Fig. 2 c, d.
4. Fig. 6d: The predictive and encoding models perform equally poorly for mouse, compared to macaque or salamander. Why does the model perform so poorly for mouse data?

(Remarks on code availability)
The webpage url is broken or is not available. I was unable to review the codebase.

Reviewer #2

(Remarks to the Author)
This paper aims at showing that many properties of the retina can be obtained by a model that aims at predicting the future stimulus frames in natural movies, while a model that would only encode the past or present stimulus would not reproduce these properties. This would suggest that the retina is organized to predict the future stimulus.

There are many issues in the present paper that need to be solved to support the claims of the authors. There is a lack of proper control, i.e. null model(s) that would give different predictions, to understand what is really important and necessary in the model proposed by the authors.

I also suspect that the results of the authors are less impressive than they claim: the model is simpler than it seems (see below), and the prediction of the future might only be necessary for one or two properties among all the ones they show. If this is the case, the paper might need to be substantially modeified to focus it on what is novel and what is really specific to the model optimized to predict the future.

Major comments:

1) The authors list several properties of the retina that are reproduced by their model, but it is often not clear if optimizing the model to predict the future is required to obtain these properties. For most of them, this is unclear. The only case where the prediction of future stimuli seems necessary is for the OSR phenomenon. If the prediction of the future is not necessary for all the other properties, it is not clear why the authors put so much emphasis on them. This also questions the whole point of the paper. For now, the model is presented as being able to reproduce everything we know about the retina. In fact, it might just be an improvement to predict the OSR (and predict ganglion cell activity), while everything else is already predicted without optimizing the parameters for the prediction of the future.
I recommend that the authors show and demonstrate very clearly, for each property, if prediction of the future is necessary or not. But if it is only necessary for the OSR, it seems that the paper needs to be reorganized to put the focus on this.

2) The description of the model is misleading. An analogy is made with the retinal circuit, but since all the feedforward network is assumed to be linear, and only recurrent connections are added, the model boils down to a LN model with the addition of recurrent connections (possibly similar to the GLM of Pillow et al, 2008). Many works have used 2 layers or even 3 layers to predict the retinal responses, so it seems surprising to do so well with only one layer. This should discussed if not tested.

It would also be necessary to visualize what the filters W look like. If the encoding model cannot reproduce the OSR phenomenon, maybe this is because mosts of the linear filters are monophasic, while they are more biphasic for the model optimized for the prediction of the future.

There is a recurrent part in the model, but its specific role is not explored. It would be good to have a control where this part is specifically removed.

Another topic which seems overlooked is the metabolic constraint: how important is it ? How robust are the results to this ? Previous works (e.g. Chalk et al, PNAS 2018) has shown that sparseness constraint can play a role when comparing prediction of the future vs compression of the past.

3) Quantification of the different properties of the retina
The authors go through several phenomena but do not focus on them carefully enough. For example, for the OSR, the initial paper showed the latency of the response changes with the period of the stimulus, by the same amount. This is not tested here, while this is a crucial property of the OSR.

The same could be said about other properties: for motion anticipation, the neural image was reconstructed, and the position of the peak firing rate relative to the moving bar was estimated for several speeds (and shown to be constant).

For the OMS, they compare a local motion vs a global motion, while the original paper showed a random motion that is either the same in the center and the far surround (eliciting no response) vs two different random motions (eliciting a response).

For the mosaics, the distance between ganglion cells of the same type should be quantified: see e.g. Ravi et al, Journal of Neuroscience 2018, for a method to estimate if there is a real tiling. For some of the mosaics shown, tiling is not even clear.

Also, I find surprising that the mosaics produced by the model are not fully covering the visual space, but only a part of it. This can be explained in experiments because not all the cells of one type are recorded, but it is less clear why you should have this in a model.

(Remarks on code availability)

Reviewer #3

(Remarks to the Author)
In this work, Taylor et al. set out to compare two highly influential perspectives on retinal coding using a novel spiking model. The first perspective, efficient coding, posits that early sensory circuits maximize encoding of information about the stream of incoming natural inputs. The second perspective, predictive coding, argues that these same systems instead extract information most relevant for predicting the future. By training their network to maximize linear decodability of stimuli several time steps into the future, they demonstrate agreement with a variety of previously observed retinal phenomena, including differing cell types, motion tuning, stimulus omission responses, and prediction of retinal firing. They argue that these results give strong support to the predictive coding model and against efficient coding.

There are many things to like about the paper. In particular:
- The work is clearly explained and results well presented.
- The use of both a spiking model and recurrence are strong positives, since these are more accurate than most rate-based models that have been used in the field. Similarly, the accounting of both graded potential and spike costs in the model makes sense biophysically, as both require energy.
- The authors consider a variety of experimental findings to which they match their model, presenting not just one line of argument but several. It is the cumulative force of these results that is the strength of the paper.

However, I have several concerns and questions that I would like to see addressed:

1. **Comparison with efficient coding:** While the framing of the paper is in terms of a test of efficient versus predictive coding, the authors mostly argue past efficient coding, focusing on phenomena explained by their model. Granted, it would be quite difficult to argue that efficient coding _cannot_ account for some particular phenomenon, but the only direct comparison I can see between the two approaches is in the analysis of Figure 6 (predicting retinal responses), which compares an "encoding" model with a "prediction" model. I assume the "encoding" model is the authors' own model predicting T = 0 steps into the future, but I couldn't seem to verify this in either the text or methods. Given the authors' framing, I would have expected to see multiple comparisons between efficient and predictive coding approaches throughout the manuscript, but in the event, this formed only a minor part of the paper.

2. **Future prediction, latency, and information:** While the authors cite Palmer and coauthors multiple times in favor of the predictive coding capacity of the retina, their own formulation of the coding problem is somewhat different in ways that may or may not be important. More specifically, Palmer et al. (and Chalk, Marre, and Tkacik, 2017, which I was surprised not to see cited) consider the predictive information about the _entire future_ given the _entire past_. This is somewhat different than the authors' formulation, which is attempting to predict a _specific point_ in the future from current neural activity. I recognize that this is necessitated in part by the need for a tractable training objective, but the assumption of a specific latency for which prediction should be optimized seems fairly ad hoc and an important theoretical caveat in the paper. While the authors do cite Palmer et al. (ll.564-66) for the 50-100 ms time frame, I believe that number refers to the _maximum_ time over which predictive information was present in that study (at least if the reference is to Fig. 3C in that work). That is, if I

recall correctly, predictive information was highest for the immediate future and fell off quickly in time. It would strengthen the paper if either some theoretical argument could be made by the authors that this specific prediction time lag is the right one or they could show that a model that maximized predictions over the _entire_ future gave the same results.

3. **Understanding model ingredients and limitations:** While the bulk of the paper is (rightly) devoted to the successes of the authors' model, I found discussion of some of its limitations lacking. For instance, it would be good to know which of the experiments the authors report rely critically on future prediction and which simply rely on the presence of recurrence in the model. Given the degree of novelty in the model ablation studies that help pinpoint the role of spikes, recurrence, and prediction time lag would be helpful. One cannot ask them to repeat these experiments for all possible combinations, but I would appreciate more clarity on which innovations are most important for explaining their results.

Along different lines, there are at least two respects in which the model appears to perform poorly in comparison with efficient coding models. The first is mentioned by the authors themselves: receptive fields in their model lack surrounds. Despite the fact that some weak surrounds have been reported in some preps, it is striking that their model does not reproduce one of the most salient features of retinal physiology, one that many efficient coding models successfully reproduce. Second, the mosaics produced by the model (Fig. 3D) are not well-formed. For example, the ON parasol cells identified in the model have substantial overlap between neighbors, as do the OFF midget cells. This is a degeneracy that may have only a mild effect in reducing predictions from the model, but it is one that is well removed by several of the efficient coding models the authors cite.

To clarify: I do not think it is important for models to get everything right, but it is likewise important here to notice what they get wrong. What the authors have provided is a compelling case that their model reproduces some interesting phenomena, but it is likewise clear that the model does a poorer job of explaining some features that efficient coding does well.

Minor questions:
- Why does metabolic cost depend on synaptic weight, exactly in (10)? Isn't the majority of energy usage just proportional to the total number of action potentials, not the input currents? Some further explanation here would be helpful.

(Remarks on code availability)
I attempted to review the code, but the repository has not been made public.

Reviewer #4

(Remarks to the Author)
In this study, Taylor and colleagues constrain a neural network model of the retina to encode predictive information and find that the model recapitulates several known properties of the retina. Overall, the study is interesting and has potentially important results. However, the study does not adequately reference the previous literature and does not provide a deeper mechanistic understanding of predictive processing. Outlined below are several points that should be addressed prior to publication.

Major comments:

1. Two previous studies have developed normative approaches to understand how prediction affects the receptive-field structure of sensory neurons. The authors cite the Singer et al paper, but the second paper is not cited (Matthew Chalk, Olivier Marre, Gašper Tkačik, 2018 PNAS DOI: 10.1073/pnas.1711114115). Besides missing a key study (in my opinion, THE most important study so far), the issue is that the Chalk et al paper covers the same ground as the current study. In other words, the current study does not build on the previous literature, which makes it difficult to understand how the current study is situated relative to previous work and to ascertain how it contributes to our understanding of predictive coding.

2. Another issue is that the paper presents properties of the model that are consistent with retinal processing (e.g., omitted stimulus response), but there is no tie to the underlying structure of the network model. What we need as a field is a more mechanistic understanding of how particular network structures give rise to functional properties. It seems that the authors would have all of the tools at their disposal to provide this – what is it about the connectivity within the network that gives rise to properties such as OSR? Without this, I think the potential impact of the network modeling is greatly diminished.

3. It is unclear to me why midget-like and parasol-like (in size) cells would fall out of a model like this. Midget cells are contributing to high spatial resolution and color vision. How is it that these cells would fall out of a normative model for prediction? Furthermore, it is difficult to determine how the constraints in the model relate to the results – e.g. What are the results if the model is constrained to have 1, 2, 4, or more cell types?

4. I attempted to review the code for this paper using the link provided, but the code was not available. GitHub gave me an error message that the page did not exist. The code for this manuscript needs to be reviewed carefully prior to publication.

Minor comments:

1. Abstract: “Our findings demonstrate that the retina is not merely a compressor of visual input…” I suggest softening this claim. The evidence for this is based solely on a computational model, no new empirical evidence is provided in this study, so “demonstrate” is much too strong a word. The findings are “consistent” with the idea that the retina is not a simple compressor of visual input.

2. Several aspects in the first paragraph of the introduction are difficult for me. First, when the authors state that the retina “sends a representation of the visual world to the brain” (lines 49-50). There are more than 20 distinct output (ganglion cell) pathways in the vertebrate retina and it is thought that each of these pathways provides a distinct representation of the visual world.

Second, lines 50-51: “Spatially downsampled by about 100 times.” This depends on cell type and species. In the primate macula, the cone signals are actually upsampled by a factor of two by the midget system.

Third, “It remains a open question what information this pattern of activity represents” (lines 51-52). I think this is also a very inaccurate statement. There has been a tremendous amount of progress understanding what these pathways represent – we likely know more about how information is represented in the retina than any other part of the brain. The literature is packed with primary studies and reviews on this subject.

Fourth, the phrase, “like a crystal ball foretelling the future” (lines 55-56) is too colloquial for a scientific journal, in my opinion. I would consider rephrasing.

3. Lines 298-302: The authors cite an example of a cell in their model that did not respond to stationary presentations of high spatial frequency gratings, but responded when the gratings moved. They then state that this cell is similar to Y-type cells in mammalian retina. However, Y cells DO respond to flashed gratings (see original Hochstein and Shapley paper [1976] as well as the significant subsequent literature [e.g., Demb et al. 2001]). These cells are defined by their nonlinear spatial summation because they have nonlinear/rectified subunits. In fact, it is puzzling how one could obtain the result described in the present work in which a cell does not respond to a flashed high spatial frequency grating, but responds to a moving grating of the same spatial frequency. This is very non-retina-like. It would be helpful for the authors to describe how this property might arise.

(Remarks on code availability)
The code was not available from the URL provided by the authors. I received an error on GitHub when I attempted to access the code.

Version 1:

Reviewer comments:

Reviewer #1

(Remarks to the Author)
The authors added substantial amounts of data to explain results that were previously unclear or ambiguous. Addition of extended data figures now highlight the details of the RFs predicted by the models, distinguishing subtle spatial and temporal variations that enable encoding of distinct stimulus features. Although, the data and expanded text in the manuscript satisfy most of my comments and comments by other reviewers, there are small details that still need attention:

1. Some of the results are described without explicit mention of the species the data are sourced from. Because spiking dynamics, latency, and RF properties can vary significantly across RGC types between species, it is necessary to clarify the species when presenting data. For example, in Figure 4, it is unclear whether the OS and DS cells are from salamander or mouse or rabbit. DS and OS cells are rare in primates, compared to rodents; hence making this distinction clear both in figure and text across the manuscript, is important.

2. Although the term ‘autoencoding model’ is intended to distinguish this framework from traditional encoding models, ‘autoencoding’ itself is a misnomer, as it may be misconstrued as an auto encoder model, which fall in the category of unsupervised neural networks trained to extract latent structure in data. I suggest using an alternative term such as ‘zero-lag encoding model’ or ‘non-predictive encoding model’.

3. Overlaps in RF mosaics are due to possible contamination from cells that may belong to other types (for example, Fig. 3 d, ON-parasol, OFF-midget). Is it because the spiking model with recurrent connections fail to account for the convergence of bipolar cell inputs or noise in data (spike sorting limitations)? Please clarify this in results or discussion.

4. A key aspect of natural viewing is dynamic changes in luminance. Multi-layered models with gain adaptation at the photoreceptor-BC synapse can better account for dynamic changes in luminance (PMID: 39009568), which may be a limitation of the existing framework. It is worth speculating in discussion, whether a predictive framework may (or not) be sufficient to capture natural luminance changes.

(Remarks on code availability)
The GitHub link works and it contains the codes used in the paper. The README file contains sufficient details for downloading, training, and reproducing results.

Reviewer #3

(Remarks to the Author)

In their revised manuscript, Taylor et al. respond to an extensive list of critiques from myself and other reviewers that all cluster around several key topics. In particular, reviewers found that some model aspects appeared ad hoc, the authors failed to discuss several important caveats and related papers, and too little effort was made to distinguish what temporal prediction and recurrence uniquely contribute to the results.

I found the authors' responses reasonable and a bit unsatisfying. The addition of ablation experiments for the recurrent connections are welcome and shed light on several concerns raised by reviewers, and clarifications about the relation between this recurrence and biology have been made at key points. On the other hand, several concerns raised by reviewers about conceptual issues, particularly relations to previous studies, have been relegated to the discussion, while the introduction remains largely unchanged.

On balance, however, the authors have succeeded in better contextualizing their findings and discussing potential limitations of their model, and in this sense my concerns have been addressed. All models are necessarily limited, and the work now better represents the pros and cons of this particular approach.

A few small points:
- p4, top: I appreciate the authors comments in the rebuttal addressing the practical points that motivate choosing a fixed time interval for prediction, but in the current manuscript, this number, 128 ms, appears in the results with no explanation or justification. The authors must make some effort to justify this choice to readers, since it is critical to the definition and success of the model.
- Extended Data Fig 5 caption: "textbfa" should be "\textbf{a}", as well as for panel d.

(Remarks on code availability)
Notebooks in codebase define paths relative to a `root` variable that expects to find/place results in `PycharmProjects/RetinalModel`, which may not exist. Also, [this line](https://github.com/webstorms/RetinalModel/blob/main/scripts/model/fits.py#L4) appears to reference a specific path that may not exist on users' machines. I may have misunderstood something, but I would strongly encourage the authors to verify that their code will run from a clean install per their own setup/install instructions to ensure that all paths work as intended.

Reviewer #4

(Remarks to the Author)
The authors have addressed all of my previous concerns.

(Remarks on code availability)

Open Access This Peer Review File is licensed under a Creative Commons Attribution 4.0 International License, which permits use, sharing, adaptation, distribution and reproduction in any medium or format, as long as you give appropriate credit to the original author(s) and the source, provide a link to the Creative Commons license, and indicate if changes were made.

In cases where reviewers are anonymous, credit should be given to 'Anonymous Referee' and the source.
The images or other third party material in this Peer Review File are included in the article's Creative Commons license, unless indicated otherwise in a credit line to the material. If material is not included in the article's Creative Commons license and your intended use is not permitted by statutory regulation or exceeds the permitted use, you will need to obtain permission directly from the copyright holder.
To view a copy of this license, visit https://creativecommons.org/licenses/by/4.0/

Reviewer 1

The manuscript by Taylor and colleagues explores a modeling framework for the retina based on integrate and fire model of retinal ganglion cell activity. The model yields receptive field mosaics of major retinal ganglion cell types encoding stimulus motifs, across different species. The model captures neural responses more reliably when trained to predict future events in a dynamic natural world.

The scientific question is important and deserves inquiry. The authors did an excellent job at developing the model and implementing it to test a fundamental principle: predictive coding that the retina might exploit for processing natural stimuli. The effort of the authors and the premise of the work, both deserve merit. However, the disconnect between the underlying assumptions of the model and the biological circuit, and disparities between the model output and empirical data in the literature, weaken the study.

Reply: We thank the reviewer for their positive assessment, and we hope that our answers below satisfy their comments.

Reviewer Point P 1.1 — The argument presented in the manuscript for including recurrent connections between ganglion cells is rather weak. Recurrent signaling is present in the retina, but not extensively. There are a few ganglion cell types are homotypically coupled, ipRGCs provide recurrent connections, others are coupled with specific amacrine cell types. While these connections can result in recurrent signaling, it is only a small number of ganglion cell types (at least in the mouse retina). Most of the ganglion cell types are still driven by glutamatergic signals from bipolar cells. The authors state that they preclude gap-junctions in their model of recurrent activity. Then what do these recurrent connections represent? And if recurrent connections do represent coupled activity, then shouldn't the model better predict responses of those ganglion cell types that are actually coupled?

Reply: We agree that we need to be clearer as to exactly what the recurrent connections in our model represent in the biology. The recurrent connections approximate any ganglion cell activity driven by other ganglion cells, either directly or indirectly. We envision the inhibitory recurrent connections of the model as approximating the effects on ganglion cells of amacrine cells that are activated by other ganglion cells via gap junctions, and the excitatory recurrent connections of the model as approximating the gap junctions between ganglion cells. While we did state this on pg. 3, we agree that the wording may be vague. Hence, to be clear, we do not preclude gap junctions, we simply do not explicitly model them, and instead wrap their effects up in the recurrent model connections. We have now made this more clear in the text. On pg. 16 we now say:

"We could also include constraints on the recurrent connectivity, such as implementing the recurrent connections by explicitly modeling the amacrine cells (Vaney 1994, Gollisch and Meister 2010) and gap junctions between the spiking units to better emulate the coupling between RGCs (Vaney 1994, Vlasiuk et al. 2021) including nonlinear effects of amacrine cells (Nategh et al, 2023)."

On pg. 18 we now say:

"The connections of the model units represent a diverse collection of pathways. The feedforward model connections reflect the effect of photoreceptors on ganglion cells via bipolar cells, including the effects of horizontal cells and those of amacrine cells that are not mediated through RGCs. The excitatory

recurrent model connections approximate the direct excitatory influence of RGCs on other RGCs via gap junctions (Vlasiuk et al. 2011) and indirectly via the rare glutamatergic amacrine cells (Lee et al. 2014). Finally, the inhibitory recurrent model connections approximate the indirect inhibitory effect of RGCs on other RGCs via amacrine cells, either by the amacrine cells impacting RGC activity directly or acting via bipolar cells, with the RGCs themselves activating the amacrine cells by gap junctions (Demb and Singer 2015)."

Furthermore, when we analyse the current contribution of the connections of the trained model, we find that the vast majority of the drive to the model units is feedforward, consistent with the limited recurrent signalling in the retina. Hence, on pg. 4 we now observe that:

"This is consistent with the recurrent connections contributing significantly less (5.6% of total) input current compared to the feedforward connections in the trained prediction model (Extended Data Fig. 2)."

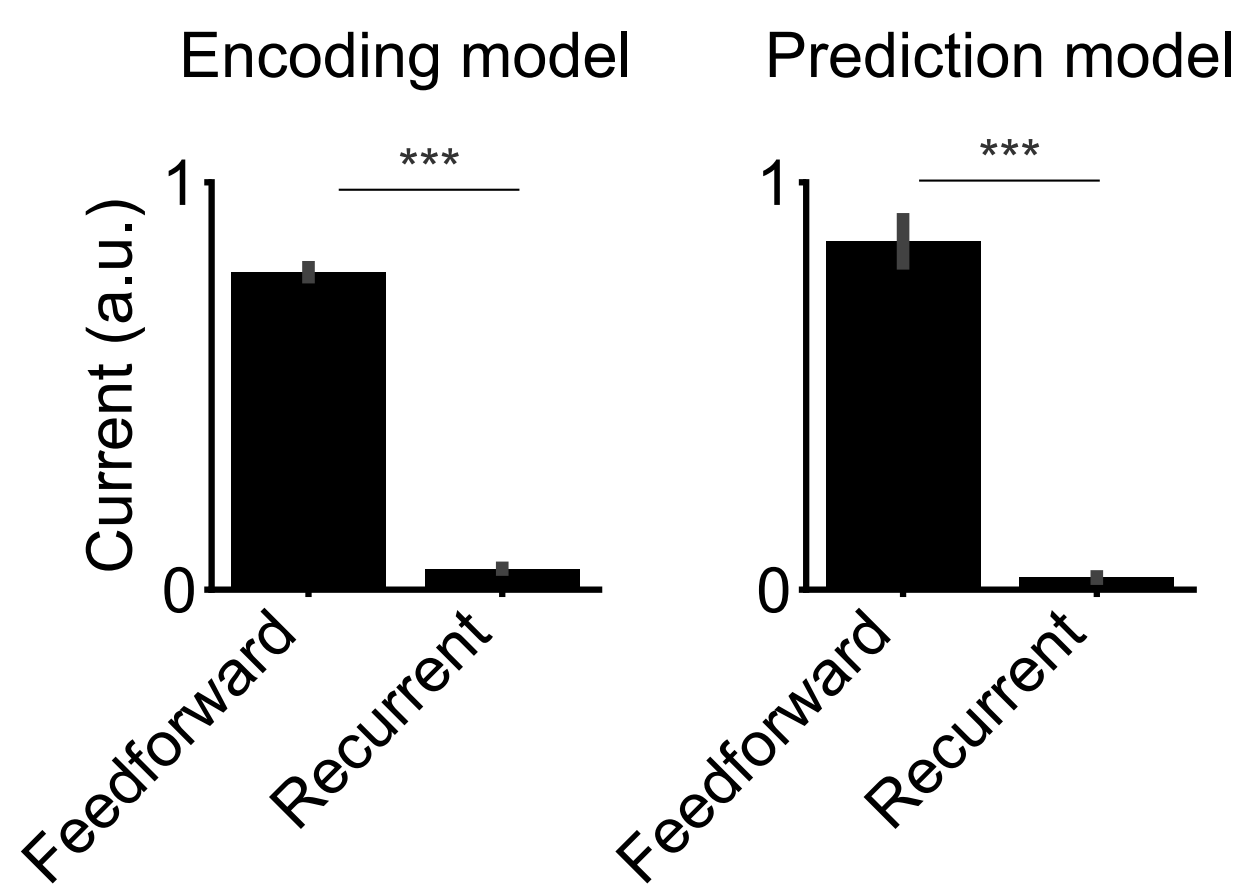

Extended Data Figure 2: Feedforward vs recurrent current contribution. Bars plot mean absolute input current and s.e. over model units using the held-out test set data (significance assessed using the one-sided Mann-Whitney U test).

Regarding your final question, the model contains both feedforward and recurrent components. Hence, we do not think that it should necessarily better predict the types of ganglion cells that are coupled than those that are not, as the model has the capacity to explain both types.

Reviewer Point P 1.2 — Since the noise term in Eq.2 is folded into the current term that sets the membrane voltage of ganglion cells, it is reasonable to surmise that this noise term is pre-spike, suggesting its origin is from upstream circuits. Photoreceptor noise (approximately Poisson-like) and stochastic vesicle release at the bipolar synapse are the major sources of this noise (see Weber et al. 2021). Maybe I am missing something but is there an explanation for why the noise should be Gaussian instead of Poisson? Full-field illumination (line 141) for a test of noise also doesn't seem the right approach because the nonlinearity at the bipolar synapse can be significantly different for patterned stimulus.

Reply: The noise term in Eq. 2 constitutes noise on all inputs to the unit, such as stochastic vesicle release at bipolar or recurrent synapses. Modelling the noise exactly was not the focus of our study, and Gaussian noise was chosen for its simplicity. Nevertheless, the noise term in Eq. 2 acts multiplicatively

upon the current, so this approximates Poisson-like noise for large mean values (Dayan & Abbott, 2001), albeit with the standard deviation rather than the variance proportional to the current. Regardless, we agree that using Poisson noise would be an improvement. We have added the following to the text to pg. 18-19:

"This approximates noise sources such as stochastic vesicle release, and while this noise model could be made more biologically accurate (Weber et al., 2021) it suffices for our purposes."

We used full-field illumination not as a test of the noise, but rather to set the model's noise level, avoiding tuning it based on the more complex stimuli used in the rest of the study. The fact that our subsequent coefficient of variation (CV) measure of noise for natural images and movies was comparable to biological CV values for natural stimuli is indicative that the noise levels we chose were appropriate. To make this clear, the relevant sentence on pg. 4 now reads:

"We set these noise sources so that the model's spike train variability resembled that of the RGCs across repeated stimulus presentations of uniform full-field illumination with randomly varying intensity (Berry et al. 1997; Meister et al. 1999), being sub-Poisson with Fano factors mostly below unity (Berry et al. 1997a, Berry et al. 1997b, Liu et al. 2022) (Extended Data Fig. 1)."

Reviewer Point P 1.3 — There are 45 ganglion cell types in the mouse retina. The authors show only one or two cell types in mouse that is predicted by the model. Why do the authors not find more cell types? Is it because of limited data, or is it because the model does not incorporate presynaptic nonlinearities, which are shaped by bipolar and amacrine cells to support feature detection?

Reply: Our aim was not to focus on one specific species (i.e. the mouse), but rather we searched for general retinal characteristics that are relatively common across species in order to make our findings more generalizable. For example, we focused on the ON- and OFF-sustained and ON- and OFF-transient division of cell types that is commonly found across different species, such as the cat X and Y cells, primate (macaque, marmoset, human) midget and parasol cells, and mouse alpha cells. The exploration of additional cell types is likely to be more challenging, as they are usually linked to cell morphology and gene expression (Goetz et al., 2022), which are not available in the model. However, we do agree that searching for additional functional cell types would be an interesting future avenue for our work. Indeed, a greater number of RGC cell types than we explore may be present in our current model, but relating the exact details of the model to the detailed retinal organization of a specific species, such as the mouse, is inconsistent with the non-species-specific nature of the model design. A model with its constraints and stimulus statistics tailored to a specific species may be more appropriate for looking for diverse species-specific cell types, such as those present in the mouse retina. Such a model would be valuable for examining the potential roles of presynaptic nonlinearities and the exact statistics of stimulus data in producing mouse RGC response types. To address this important point we have added the following to the Discussion on pg. 16:

"We did not focus on one specific species, but rather searched for general retinal characteristics that are relatively common across species. For example, we looked for the ON- and OFF-sustained and ON- and OFF-transient division of cell types that is common across many species, such as the cat X and Y cells (Sherman and Spear 1982), primate (macaque, marmoset, human) midget and parasol cells (Watanabe and Rodieck 1989), and mouse alpha cells (Goetz et al., 2022). It would be interesting in future studies to tailor the model to the particular stimulus statistics and constraints of individual species. This might better capture, for example, mouse retinal responses to natural stimuli, which our models predicted somewhat worse than for the other species we examined. It might also explain species-specific

distinctions between RGC types, for example in the extent to which they exhibit direction selectivity (Dhande et al., 2019)."

We also now further emphasize the value of including presynaptic nonlinearities on pg. 16:

"Furthermore, we modeled the photoreceptors and bipolar cells as a linear transformation, given reports of their near-linear integration properties (Baccus and Meister 2002). However, bipolar cells can exhibit some non-linear integration (Schreyer and Gollisch 2021), thus modeling them with an additional layer of non-linear graded units could be considered"

Reviewer Point P 1.4 — The authors use spike rate and CV for comparing model responses with responses of actual neurons. But these metrics are inadequate in capturing the temporal dynamics of neurons driven by correlations in the natural stimuli. The authors should consider including alternative metrics such as cross-correlations or synchrony or spike train distance for cross-validating the results.

Reply: Thank you for this suggestion. We now also compare the cross-correlations of the responses of the prediction and autoencoding model units and the actual neurons for the natural stimuli (updated Fig. 2C and D), and have included the following in our manuscript pg. 5:

"We measured the correlation between the spike trains of different units by calculating the spike train cross-correlogram, which assesses the correlation between randomly selected pairs of units at different time lags. For pairs of RGCs, the peak correlation is at time lag zero for the image (Fig. 2c) and movie datasets (Fig. 2d). A peak at zero was also seen for the prediction and autoencoding models, with the peak for the prediction model being closer to the biology. The spike train correlations symmetrically decayed for increasing and decreasing lag for both models, as with the biology. However, for the movie dataset, both the prediction and autoencoding model units tended to be more correlated for non-zero lags compared to mouse RGCs. These elevated correlations persisted when we ablated the recurrent connections in the prediction model (although the peak correlation was slightly decreased). For both datasets, the height of the peak correlation is closer to that of real neurons for the prediction model than for the autoencoding model."

On pg. 22 the Methods section of our manuscript has also been updated to include the details of this analysis.

"We measured the neural synchronization between the spike trains of pairs of RGCs or pairs of model units, by calculating their cross-correlogram. This is the Pearson correlation coefficient between the spike trains of two units for varying times lags. To obtain the average cross-correlogram over the population, we calculated cross-correlograms for 1000 randomly sampled unit pairs and averaged them. We binned the spike trains using a bin size of 33ms before calculating the cross-correlograms."

Reviewer Point P 1.5 — Responses of model units appear strongly correlated in time (Figure 2 a, b) much more than actual neurons. I wonder if this is an artifact of the recurrent term since that can force a large population of cells to fire synchronously to different phases of the stimulus. If that is not the case, then do those correlated cells belong to the same type?

Reply: We found the cross-correlations at lag zero to be similar for the prediction model and the actual neurons (see reply to previous comment). However, the model's cross-correlations are larger for increasing and decreasing lag compared to the real neurons (updated Fig. 2c and d). Ablating the recurrent connections did not substantially alter the model's response correlations (see above). On pg. 6 we have now added the following to the main text regarding the correlations between the different model unit types:

"We observed the response correlations between the same functional unit classes to be larger than the correlation between the different classes (Extended Data Fig. 4). The ON parasol-like units exhibited the largest within-class correlations, as has similarly been reported in the biology (Karamanlis et al. 2023)."

We have also created the following Extended Data Figure:

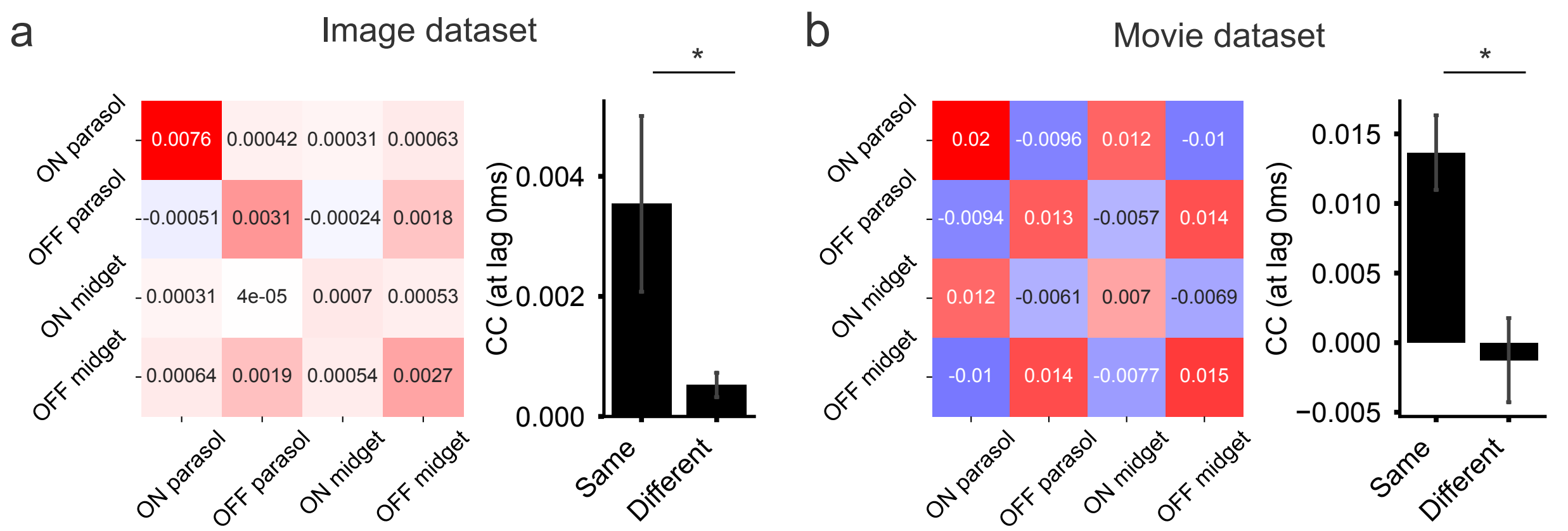

Extended Data Figure 4: Response correlations between functional classes. a. Left: response correlations between the same and different unit functional classes of the prediction model for the image dataset. Right: bar plot comparing the correlations within the same class to the correlations between different classes. Bars plot mean and standard error, with statistical significance assessed using the Mann-Whitney U test (*$p < 0.05$). All correlations are taken from the cross-correlogram at lag zero. **b.** The same as in **a.**, but using the response correlations obtained for our movie dataset.

Reviewer Point P 1.6 — Does the model yield receptive field mosaics of other major ganglion cell types in salamander and mouse? If it does, then the authors should show the results at least for a few other cell types in a separate figure or as a supplement.

Reply: We did not explore any species-specific cell types in our model other than the general ON- and OFF-transient and ON and OFF-sustained retinal cell types. This is for the reasons we give in our reply to P1.3. However, we now include a supplementary figure of all spatial receptive fields for both the prediction (new Extended Data Fig. 3) and autoencoding models (new Extended Data Fig. 6).

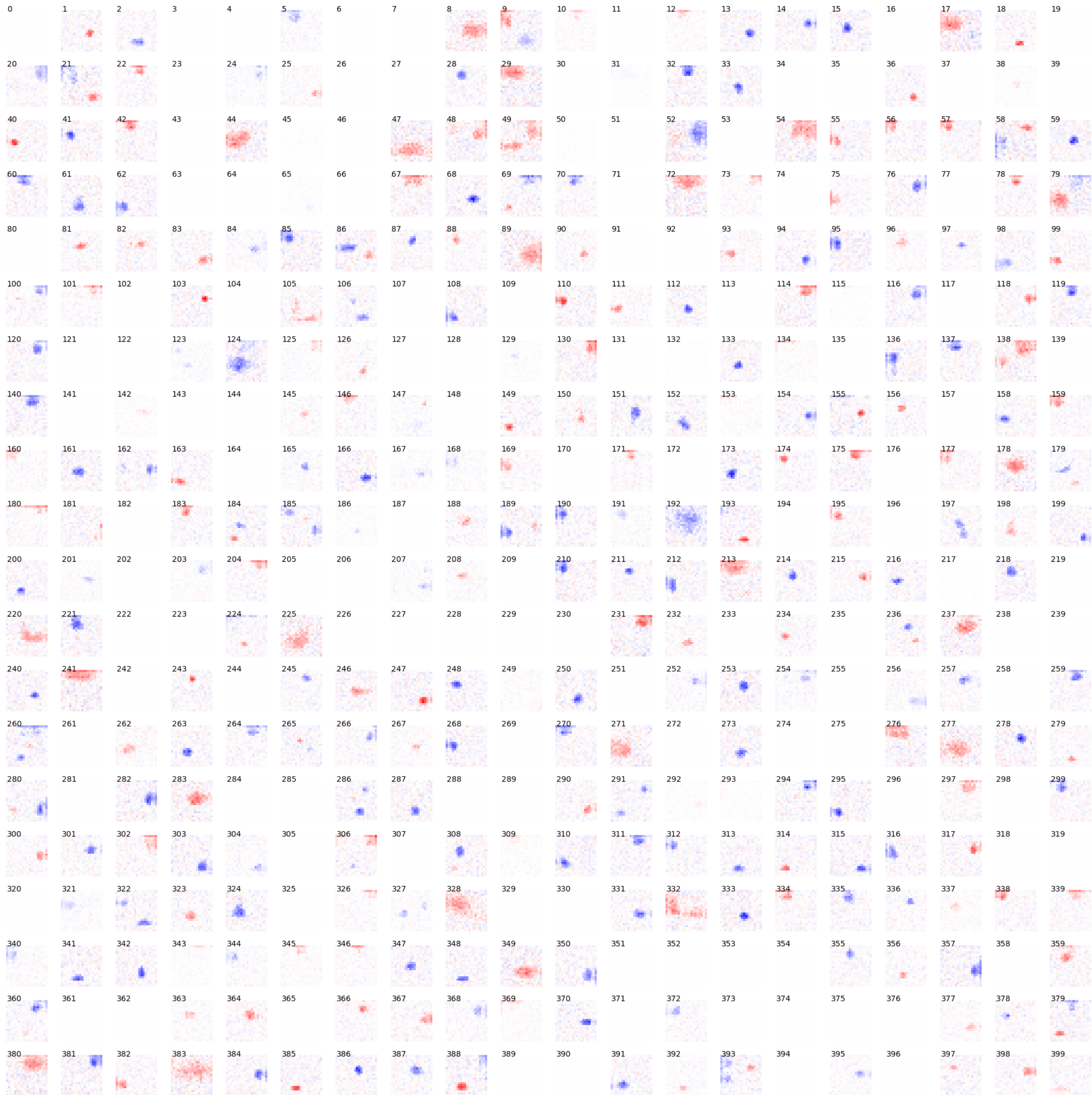

Extended Data Fig. 3: Prediction model spatial RFs. Every square corresponds to each unit's spatial RF with largest power over time (blank squares denote dead units).

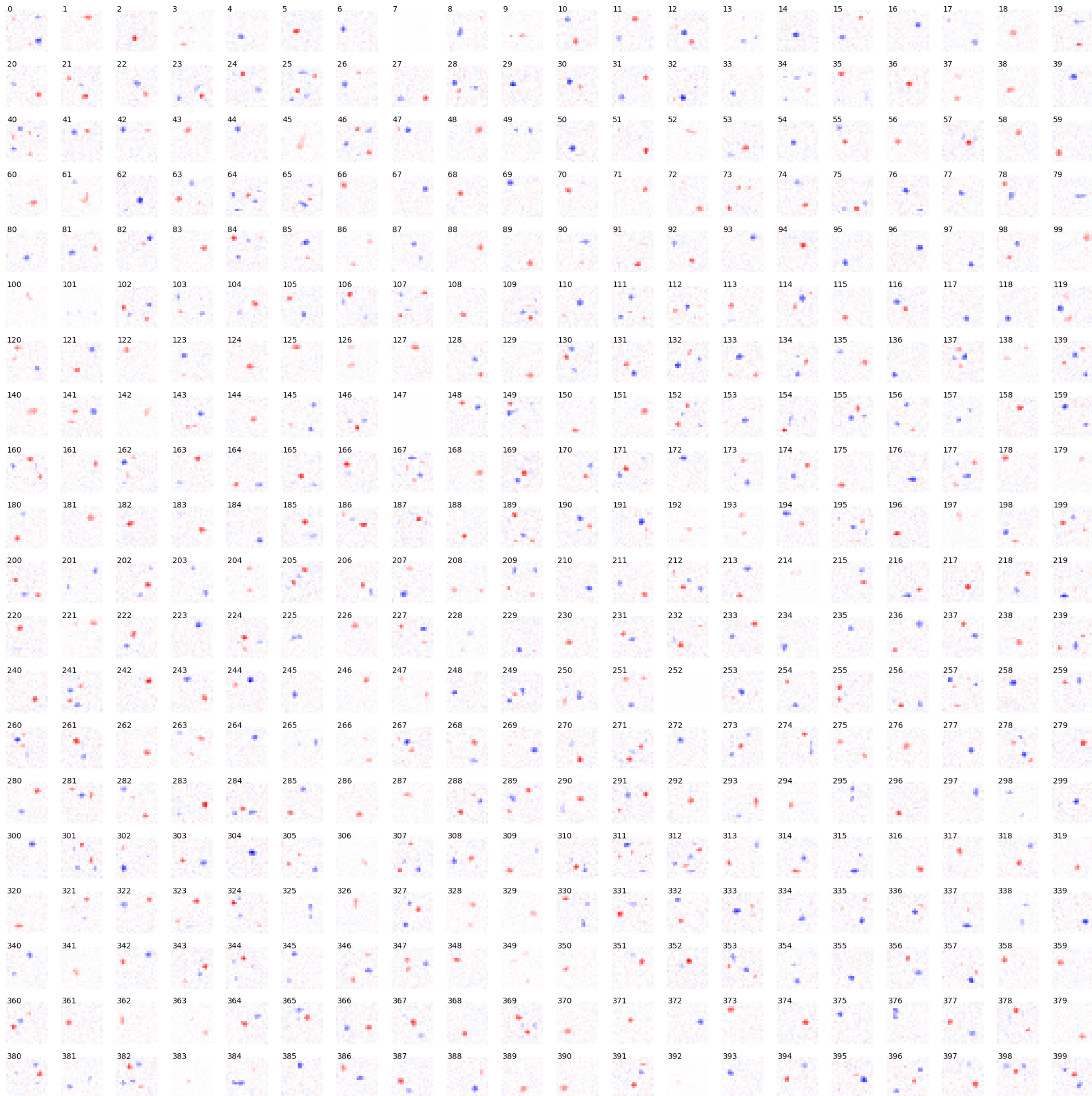

Extended Data Fig. 6: Autoencoding model spatial RFs. Every square corresponds to each unit's spatial RF with largest power over time.

Reviewer Point P 1.7 — Why does the model not produce a RF surround? Studies by Karamanlis-Gollisch, and others, suggest that not accounting for nonlinearities or subunits in bipolar cells may be a factor. The authors should provide an explanation, or at the minimum speculate in Discussion. Also, the temporal RFs of model units are 4 times faster than those estimated from actual neurons (Fig. 3b). The authors should clarify if this was expected or if it is a shortcoming of the model.

Reply: In our original submission, on pg. 15 (amended text now pg. 16) we already discussed reasons why our model units may not display RF surrounds:

"We could also explore the effect of noise in the model. The RFs in our model lack a clear surround, whereas the RFs of RGCs have typically been characterized as center-surround (Gollisch and Meister 2010). Perhaps learning the noise sources in the model, instead of hardcoding them, would result in RFs with surrounds (Karklin and Simoncelli 2011). However, it remains unclear to what degree RGCs exhibit RF surrounds, as several studies report RFs with little to no surrounds (Chichilnisky and Kalmar 2002, Kling et al. 2020). It is possible that these differences result from the way the RFs are mapped, where spike-triggered averages - as employed in our study - have been shown to result in RFs with less defined surrounds (Shi et al. 2019)."

However, thank you for prompting us to explore this further. After some investigation, we have found that many units of the model often do, in fact, have surrounds, though the peak power of these surrounds is slightly delayed relative to the peak power of the receptive field centers. For the spatial receptive fields, we took the time point of the spatiotemporal receptive field with the largest overall power. At this time point the surround is weak or absent, but just a few time points further into the past there is often a clear surround. Notably, in the biology, the surround also tends to lag the center (Kaplan & Bernardete 2001, Borghuis et al. 2017), suggesting that this biological phenomenon may not just be a mechanistic consequence of the circuitry, but it may also serve a functional purpose for improving temporal prediction. We also found that increasing the number of samples used to estimate the receptive fields and using a more sensitive colormap made the surrounds of the model units more apparent. Given this, we have now added the following to the Results on pg. 8:

"Note that the spatial receptive fields were taken at the time point of each spatiotemporal receptive field with the largest overall power. At this time point, the surround is weak or absent (Fig. 3b); however, tens of milliseconds later into the past the model units often have a clear surround (Extended Data Fig. 8). Notably, in the biology, the surround also tends to lag the center (Kaplan & Bernardete 2001, Borghuis et al. 2017), suggesting that this biological phenomenon may not just be a mechanistic consequence of the circuitry, but may also serve a functional purpose for improving temporal prediction."

We have also now added the following Extended Data Figure, that illustrates the surrounds in the spatiotemporal receptive fields:

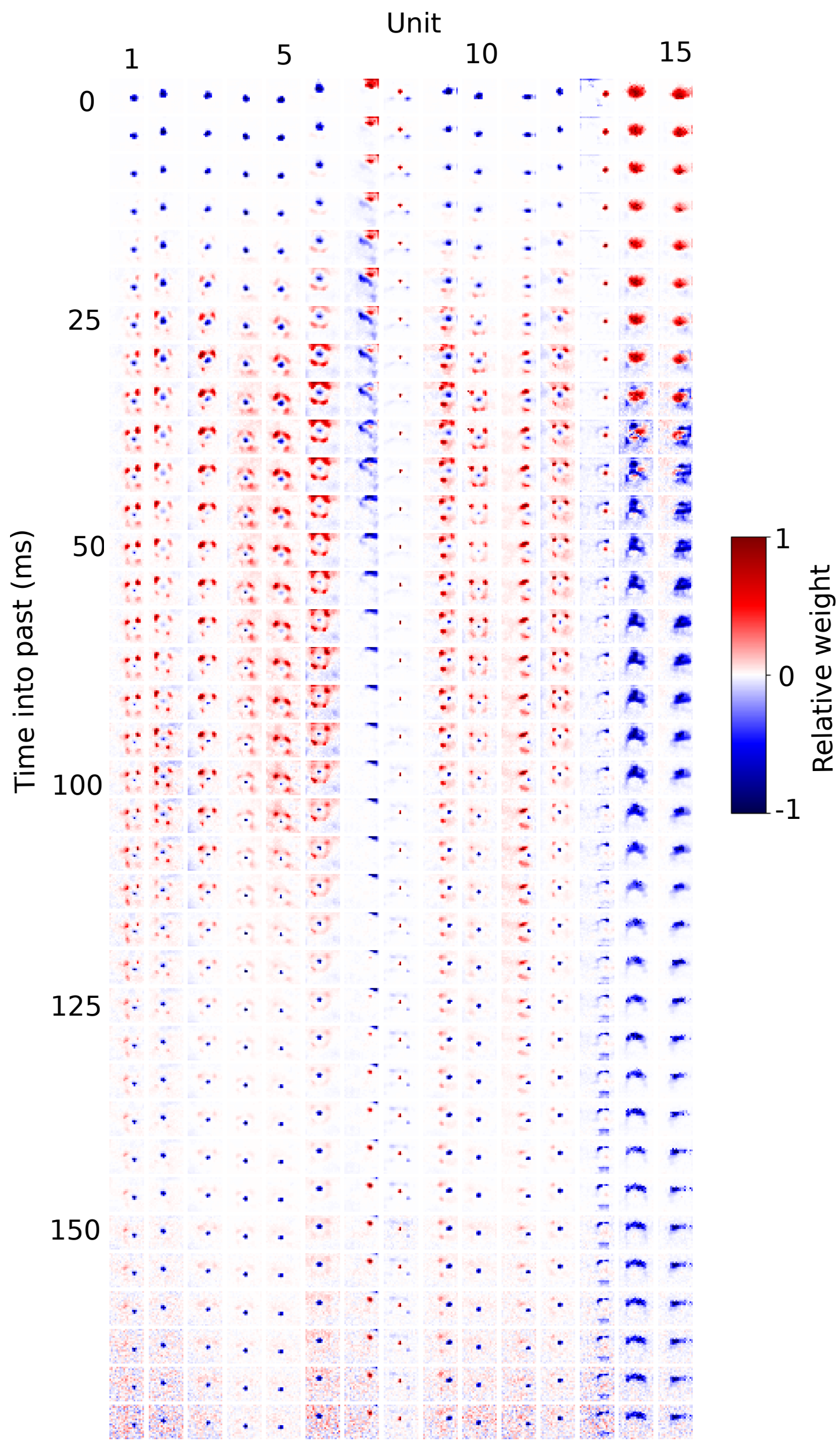

Extended Data Figure 8: Example spatiotemporal receptive fields showing lagging surrounds. The receptive fields were measured using five hundred times more noise clips of twice the length than for the other figures. Also, to display the RF, each time point was independently normalized and a more sensitive colormap was used.

Consequently, we have modified the paragraph in the discussion where we originally discussed the surround to now read (pg. 16):

"We could also explore more the effect of noise in the model. RGCs have typically been characterized as center-surround (Gollisch and Meister 2010). While the RFs of the model often show lagged surrounds, as has been reported in the biology (Kaplan & Bernardete 2001, Borghuis et al. 2017), perhaps learning the noise sources in the model, instead of hardcoding them, would result in RFs with

stronger surrounds (Karklin and Simoncelli 2011). However, it remains unclear to what degree RGCs exhibit RF surrounds, as several studies report RFs with little to no surrounds (Chichilnisky and Kalmar 2002, Kling et al. 2020). It is also possible that differences in surrounds can result from the way RFs are mapped, where spike-triggered averages - as employed in our study - have been shown to result in RFs with less defined surrounds (Shi et al. 2019)."

We have also added the following to the Methods (pg. 23):

"The exception was the spatiotemporal receptive fields in Extended data Fig. 8, which used 1,000,000 white-noise input clips of 200-frame duration."

You are correct, the temporal RFs of the model units appear faster. This is expected as the input to the model units is not constrained to be integrated over time, in contrast to photoreceptors which are required to integrate incoming light energy over time due to slowness of the transduction mechanism. We now acknowledge this, and the potential role of nonlinearities in bipolar cells in creating RF surrounds, in our discussion on pg. 16:

"However, bipolar cells can exhibit some non-linear integration (Schreyer and Gollisch 2021), thus modeling them with an additional hidden layer of graded units could be considered. Also, it may be worth applying to the clips some low-pass temporal integration reflecting the relative slowness of the phototransduction, alongside substantially limiting the span of input latencies. This may address the difference in the temporal RF profile of the model units compared to RGCs, and also perhaps help to produce stronger RF surrounds (Karamanlis and Gollisch, 2023) and a stronger role for recurrent connections."

Reviewer Point P 1.8 — Is the predictive model (line 430) supposed to predict the retinal output to natural images, or predict responses of V1 neurons from retinal output? The authors refer the Cadena paper which focused on predicting V1 neuron responses, which has a different design and different underlying assumptions that a CNN based model that predicts ganglion cell responses from natural images/movies. For example, filters emulating RFs in the retina are not Gabor-like and V1 response is influenced strongly by recurrent and feedback signals unlike the retina. How are these differences reconciled?

Reply: We fitted a linear-nonlinear mapping to predict RGC responses from the activity of the predictive and autoencoding retinal models. We did not predict any responses of V1 neurons and have removed the Cadena reference to avoid any confusion.

Reviewer Point P 1.9 — To test OSR, the flash needs to be absent in a sequence of period flashes. The stimulus trace in Extended Data Fig. 2 shows something different, in that the flash is not omitted, rather the absence of flash is omitted. The frequency (16hz==62.5ms period) also does not correlate with what is described in methods: 80ms duration following termination of flash. The authors should explain this discrepancy, and/or retest the model output with the correct stimulus.

Reply: You are correct that a flash needs to be omitted from the stimulus flash-sequence to test for an OSR. Indeed, this is what we did in Extended Data Fig. 2. (now Extended Data Fig. 11). However, to clarify, instead of omitting an "on" light flash, we omitted an "off" light flash in accordance with the original analysis by Schwartz et al. 2007 (see their Fig. 1a and b). We have updated the figure captions and methods to make this more explicit. On pg. 10 in the caption of Fig. 5d it now reads:

"Firing rate of a single salamander RGC (Schwartz et al. 2007) (top) and of two example model units (bottom) during a sequence of 16 40-ms dark-flashes (brief luminance reductions) presented at 12Hz.

The dashed line marks the time when the dark-flash sequence was halted, with the arrow indicating the omitted-stimulus response. Omission stimulus responses were also present at different dark-flash rates, as well as when dark-flashes were omitted in the middle of the sequence (Extended Data Fig. 11)."

Extended Data Fig. 11 caption now reads:

"**Omission stimulus responses. a.** Example model unit responses to a sequence of dark-flashes flashing at 8 Hz. **b.** Example model unit responses to a sequence flashing at 16Hz with a dark-flash omission in the middle of the sequence. The dashed line marks the time when the dark-flash was omitted, with the arrow indicating the omitted-stimulus response."

On pg. 23 the Methods now read:

"RGCs exhibit OSRs at the end of a dark-flash sequence around the expected time of the next dark-flash."

Minor comments

Reviewer Point P 1.10 — Line 661: The authors state that majority of amacrine cells use spikes. This is not true. Most of the amacrine cells respond through graded potentials (there are a few displaced amacrine cell types that do spike). The 'spikes' in the references are calcium spikes, which can result in action potentials but not always.

Reply: We have now removed this statement.

Reviewer Point P 1.11 — The phrases 'predictive model', 'predicting neural responses', 'efficient prediction', 'optimized for prediction' have been used interchangeably across the manuscript. These phrases have different meanings, and the authors should carefully consider how these phrases are being used and where.

Reply: Thank you for pointing this out. We have now more carefully considered how these phrases are being used. For example, now when we first mention our central model we introduce it as "efficient temporal prediction" so that any future time we refer to the "predictive model" or "temporal prediction" or "efficient prediction" it is clear that we are referring to the same model.

Reviewer Point P 1.12 — P-values needed in Fig. 2 c, d.

Reply: As requested, we have added P-values for Fig. 2c and d, where on pg. 4 we say:

"Similarly, we found the prediction model units to exhibit a lower firing rate (images 8.3Hz vs movies 17Hz; $p = 6.0 \times 10^{-14}$, one-sided Mann-Whitney U test) and CV(ISI) (images 0.38 vs movies 1.9; $p = 2.4 \times 10^{-166}$, one-sided Mann-Whitney U test) to the images compared to the movies."

Reviewer Point P 1.13 — Fig. 6d: The predictive and encoding models perform equally poorly for mouse, compared to macaque or salamander. Why does the model perform so poorly for mouse data?

Reply: The prediction model performs about 35% better on the mouse movie data than the autoencoding model. Although this is hard to make out in Figure 6d, it is apparent in Fig. 6c. Nonetheless, it is still the case that both models perform worse for the mouse than for the macaque or the salamander data. The reason that the models perform better for the salamander data is most likely because the

salamander results are for images, whereas the mouse results are for movies, the dynamics of which add additional difficulty to the modelling of retinal responses. However, it is still the case that the mouse movie results are about 40-50% worse than the macaque movie results (and the marmoset movie results for that matter). The reason for the poorer performance here of the models for the mouse may be due to species differences, which may include hard-to-capture non-linearities, or it may be due to differences in the stimulus set or experimental setup. We now point out this performance difference for the mouse - see the new text in our response to your major point P1.3.

Reviewer 1 (Remarks on code availability)

Reviewer Point P 1.14 — The webpage url is broken or is not available. I was unable to review the codebase.

Reply: Apologies for this, the codebase is now online.

Reviewer 2

This paper aims at showing that many properties of the retina can be obtained by a model that aims at predicting the future stimulus frames in natural movies, while a model that would only encode the past or present stimulus would not reproduce these properties. This would suggest that the retina is organized to predict the future stimulus.

There are many issues in the present paper that need to be solved to support the claims of the authors. There is a lack of proper control, i.e. null model(s) that would give different predictions, to understand what is really important and necessary in the model proposed by the authors.

I also suspect that the results of the authors are less impressive than they claim: the model is simpler than it seems (see below), and the prediction of the future might only be necessary for one or two properties among all the ones they show. If this is the case, the paper might need to be substantially modeified to focus it on what is novel and what is really specific to the model optimized to predict the future.

Reply: We thank the reviewer for their assessment, and we hope that our answers below satisfy their comments.

Reviewer Point P 2.1 — The authors list several properties of the retina that are reproduced by their model, but it is often not clear if optimizing the model to predict the future is required to obtain these properties. For most of them, this is unclear. The only case where the prediction of future stimuli seems necessary is for the OSR phenomenon. If the prediction of the future is not necessary for all the other properties, it is not clear why the authors put so much emphasis on them. This also questions the whole point of the paper. For now, the model is presented as being able to reproduce everything we know about the retina. In fact, it might just be an improvement to predict the OSR (and predict ganglion cell activity), while everything else is already predicted without optimizing the parameters for the prediction of the future. I recommend that the authors show and demonstrate very clearly, for each property, if prediction of the future is necessary or not. But if it is only necessary for the OSR, it seems that the paper needs to be reorganized to put the focus on this.

Reply: For any given phenomenon, there might be a number of different normative principles that could potentially explain it. The strength of temporal prediction is that this single principle can explain so many different neural response properties at once, rather than having separate principles for each one. This is why we explore multiple retinal properties, including those compatible with other computational principles, in order to find a unifying principle that can explain multiple aspects of retinal function. Indeed, different normative principles may in fact subserve temporal prediction; for example, distinct midget and parasol cells may be needed to capture the small-scale detail and large-scale dynamics of natural scenes, but this property of spatiotemporal coverage may in turn be needed to subserve the more general goal of efficient temporal prediction in the scenes. Furthermore, there are properties, such as the OSRs, which perhaps only temporal prediction can explain.

Additionally, and importantly, as we now show in more detail, temporal prediction both quantitatively and qualitatively outperforms the alternative model of a compressed encoding of the current stimulus input. Hence, in addition to the lack of OSRs we observed for the autoencoding model, we now also provide additional side-by-side comparisons between the predictive and autoencoding model:

1. The predictive model better captures the firing rates and spiking variability of RGCs compared to the autoencoding model (updated Fig. 2c and d). On pg. 4 we now say: "The autoencoding model exhibited similar spike statistics to the prediction model, albeit slightly less aligned to the biology with respect to the CV(ISI)."

2. The predictive model exhibits more realistic correlations between spike trains compared to the autoencoding model (new addition to Fig. 2c and d), with the autoencoding model over-estimating the correlations at lag equal to zero. See our response to P1.4.

3. In contrast to the temporal prediction model, the spiking autoencoding model does not exhibit large parasol-like spatial RFs (new Extended Data Fig. 6). Additionally, and unlike the biology, many of the RFs are not uni-peaked and oval shaped, but rather display power in various disjoint regions of the frame. To this end on pg. 6 we now say: "The RGC-like RF tuning properties were less evident in the autoencoding model in which we found the parasol-cell like RFs to be missing (Extended Data Fig. 6)."

Most importantly, we found that the predictive spiking model better captures the recorded RGC responses to natural images and movies across different animal species compared to the autoencoding model. In our opinion, this is our strongest evidence in favour of the retina employing predictive processing.

Hence, it is a unifying single principle we a looking for, and while individual phenomena may also be compatible with alternative principles, we know of no principle which captures such a breadth of phenomena as temporal prediction.

To emphasize this general point we have added the following to the Introduction on pg. 2:

"While individual retinal properties may each reflect different functions, the breadth of phenomena that can be explained by efficient temporal prediction suggests that many of these distinct functions subserve this overall simple objective."

Reviewer Point P 2.2 — The description of the model is misleading. An analogy is made with the retinal circuit, but since all the feedforward network is assumed to be linear, and only recurrent connections are added, the model boils down to a LN model with the addition of recurrent connections (possibly similar to the GLM of Pillow et al, 2008). Many works have used 2 layers

or even 3 layers to predict the retinal responses, so it seems surprising to do so well with only one layer. This should discussed if not tested.

Reply: We believe that the general and flexible nature of our model - comprising a simple single-hidden layer of recurrently connected spiking units - is a strength of our approach. We discuss why this is the case, and why our model does surprisingly well with only one layer, in a new paragraph of the Discussion on pg. 15-16:

"It is striking that a single layer network, with many simplifications compared to the biology, reproduces so many features of the retina. This may partly be because these simplifications, such as linear photoreceptor/bipolar layers and ignoring the details of amacrine cells and gap junctions, still appropriately capture many of the crucial features for the response properties examined. However, this finding also suggests that the response properties of the network (and the retina) are more a consequence of the objective and stimulus statistics than the details of the architecture, and that different architectures may achieve the same functional results when optimized for temporal prediction. Indeed, this was our reason for using a rather generic recurrent architecture, rather than incorporating lots of species-specific biological detail, in order to show that diverse retinal features could arise as a consequence of a temporal prediction objective and stimulus statistics, rather than be contingent on particular biological structures that are hard-coded into the model.

This is not to suggest, however, that including more biological detail would not further extend the explanatory power of our spiking temporal prediction model."

That being said, it would be interesting in future work to add more layers. We have expanded the Discussion to address this (see our reply to Reviewer point P1.7). Also, to clarity the similarities and differences between our model and LN and GLM models, we have added the following sentences to the Methods on pg. 18:

"We modeled the RGCs in the retina using a single hidden layer of $N = 400$ recurrently-connected spiking units, providing a flexible model for optimization to an objective. Unlike simpler linear-nonlinear models (Singer et al. 2018; 2023), which lack spikes, and generalized linear models (Pillow et al., 2008), which use a Poisson-process spike generator, we simulate our units using the leaky-integrate and fire model. This model - like actual neurons - includes an evolving membrane potential and a threshold-governed spiking output with a reset mechanism."

Of course, another major difference between our model and basic LN and GLM models is that our model is normative, being trained for the hypothesized function of temporal prediction, rather than being simply fitted to retinal data. Hence, our models reproduce retinal properties from first principles and provide a method to test general principles of retinal function, rather than just provide a compact description of the experimental data.

Reviewer Point P 2.3 — It would also be necessary to visualize what the filters W look like. If the encoding model cannot reproduce the OSR phenomenon, maybe this is because mosts of the linear filters are monophasic, while they are more biphasic for the model optimized for the prediction of the future.

Reply: As the reviewer suggests, we found the units in the predictive model with an OSR to exhibit biphasic input weights (W) filters (new Extended Data Fig. 13a) and that the input weight filters in the autoencoding model were monophasic (new Extended Data Fig. 13c). Predictive model input weight filters for units without an OSR were also less biphasic (new Extended Data Fig. 13b). We also saw comparable results when we looked at the corresponding spatiotemporal receptive fields of the

units (new Extended Data Fig. 14). We have added the following text to the Results on pg. 11-12, referencing Extended Data Figs. 13 and 14:

"Inspection of the input weights of the OSR units revealed them to often have a biphasic temporal profile - with the net weighting changing from positive to negative as the weights extend into the past, or with decreasing negativity and then a slight increase (Extended Data Fig. 13). This biphasic tendency was also reflected in their receptive fields (Extended Data Fig. 14). We observed these properties much less in the autoencoding model units and in predictive model units that did not produce OSRs. We confirmed these differences between OSR and non-OSR units with a quantification of the biphasic character of the input weights (Extended Data Fig. 15), although there was considerable overlap between OSR and non-OSR units."

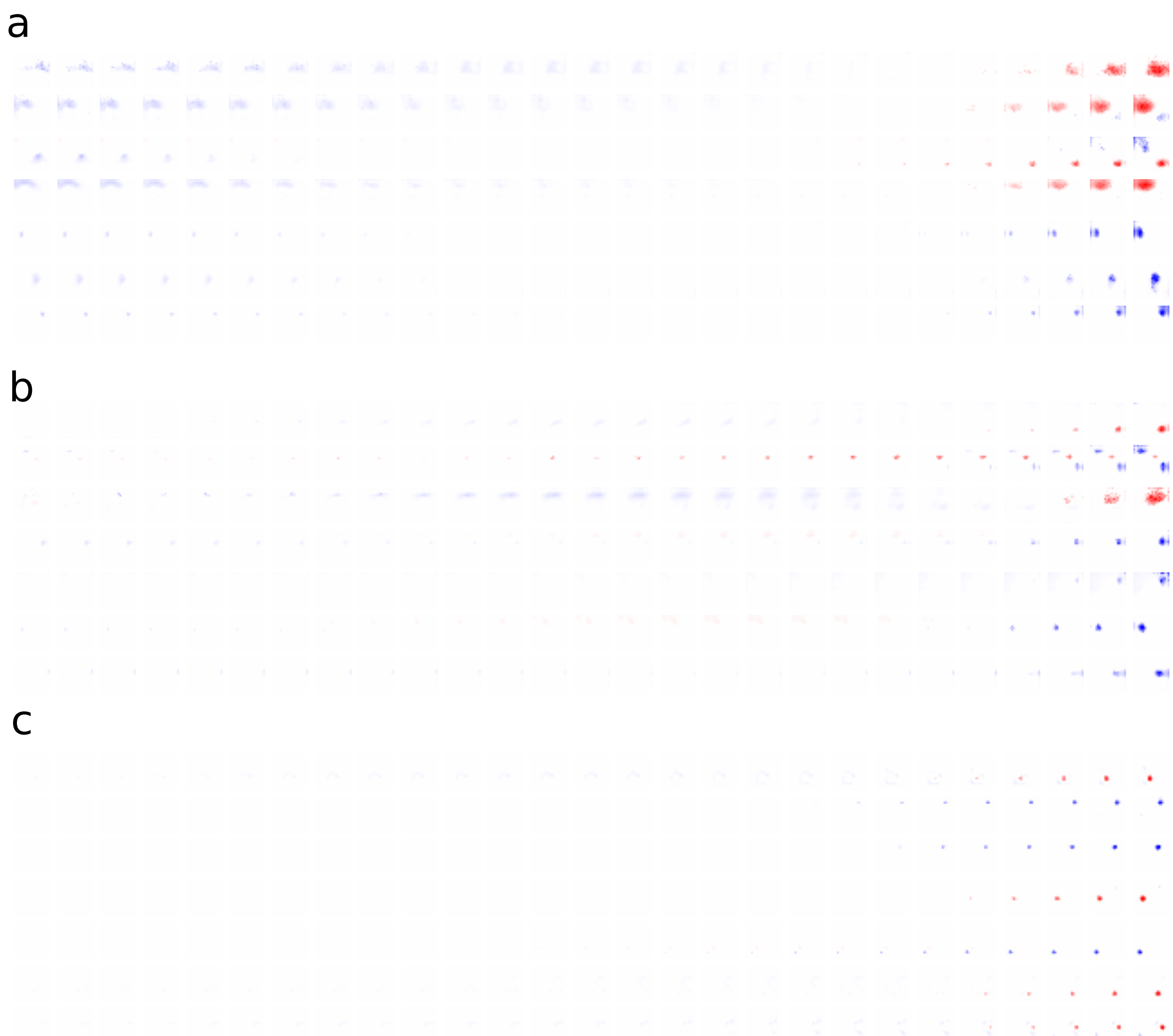

Extended Data Fig. 13: Input weights of model units with (and without) OSRs. a. Every row depicts the input weights, W, of a unit with an OSR from the prediction model. **b.** Every row depicts the input weights of a unit without an OSR from the prediction model. **c.** Every row depicts the input weights of a unit from the autoencoding model for which we found no OSRs.

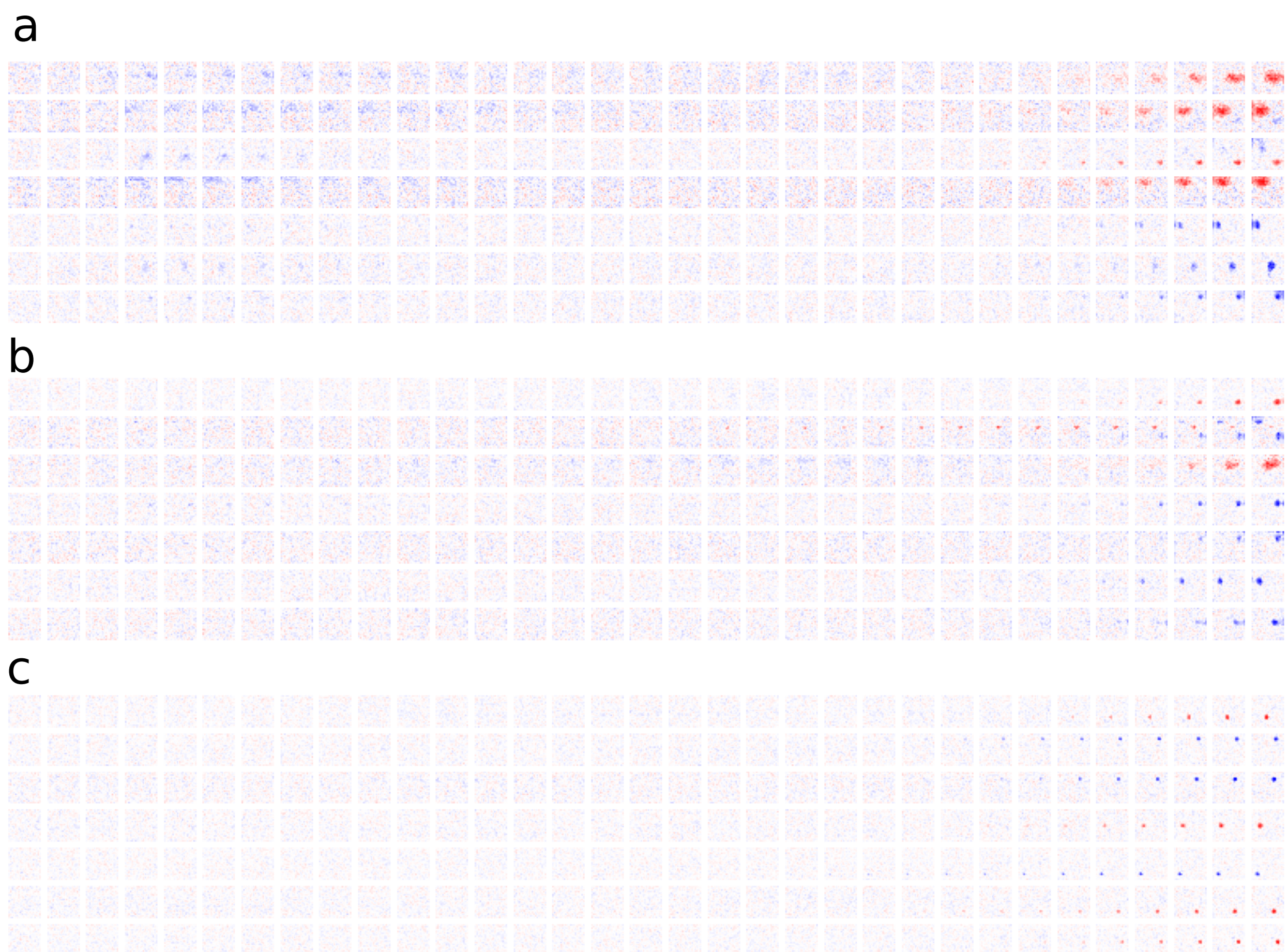

Extended Data Fig. 14: Spatiotemporal RFs of model units with (and without) OSRs.
a. Every row depicts the spatiotemporal RF of a unit with an OSR from the prediction model.
b. Every row depicts the spatiotemporal RF of a unit without an OSR from the prediction model.
c. Every row depicts the spatiotemporal RF of a unit from the autoencoding model for which we found no OSRs.

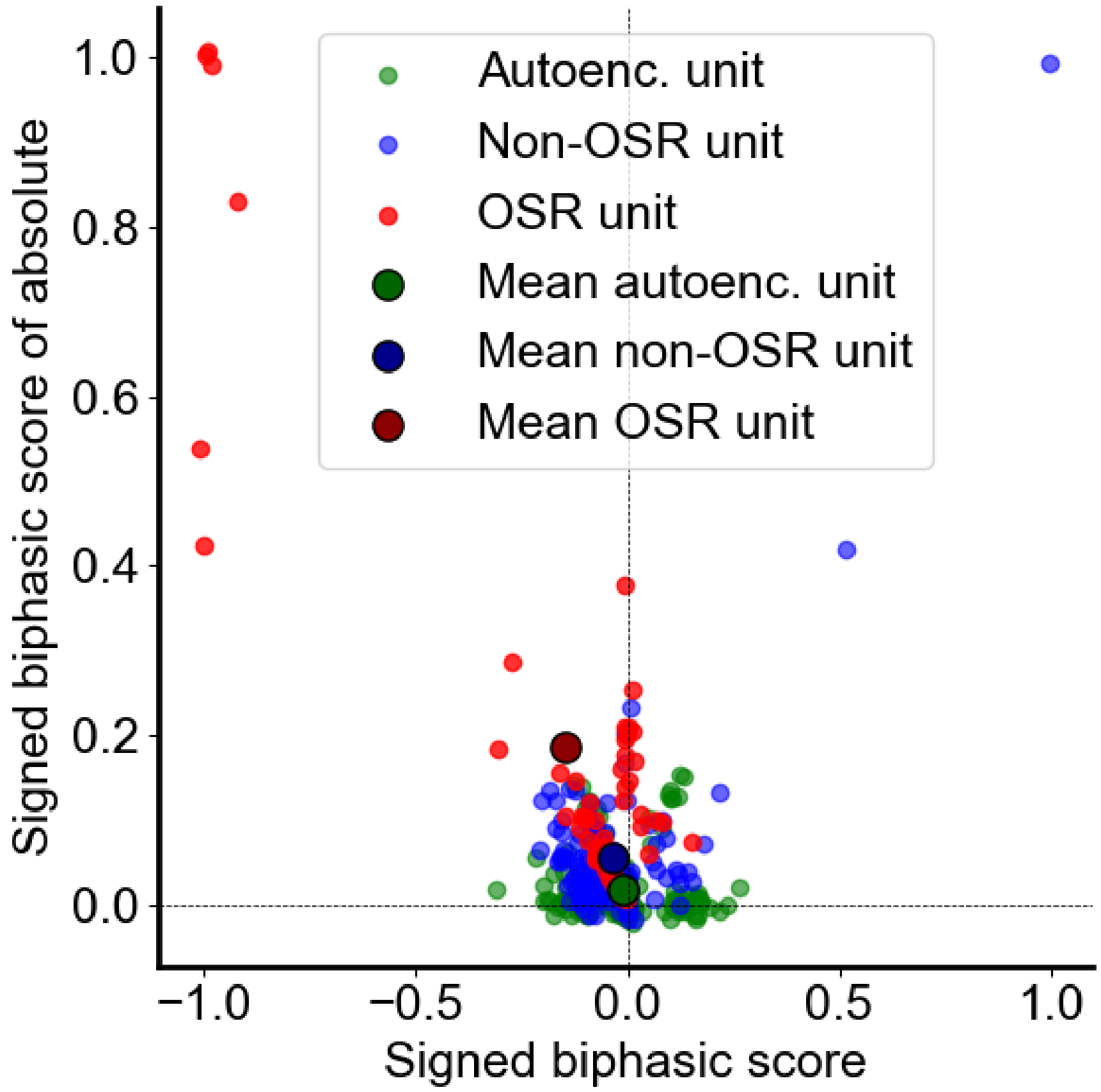

Extended Data Fig. 15: Measurement of the bi-phasic character of the input weights. First, we averaged the input weights over space for each latency, giving an array of 30 values extending into the past. The first, most recent, value typically has the largest magnitude. If this first value (z_1) was positive, then we found the minimum value of the array (z_{min}), and measured the highest value to the past of it ($z_{rebound}$). We then measured $(z_{rebound} - z_{min})/(z_1 - z_{min})$. If this first value ($z_1$) was negative, then we found the maximum value of the array (z_{max}), and measured the lowest value to the past of it ($z_{rebound}$). We then measured $(z_{rebound} - z_{max})/(z_1 - z_{max})$. The measure was then clipped between -1 and 1. Hence, a measure above -1 and below 0 indicates an array that dips partially after a rise, 0 indicates a monotonic array, and above 0 and below 1 indicates an array that rises partially after a dip, with the magnitude giving the degree of return. We calculated this measure for each OSR unit and non-OSR unit of the prediction model, defining an OSR unit as a unit that showed an OSR for at least one sequence rate. We also calculated this measure for the autoencoding model units. This measure provides the x-axis of the plot. The y-axis is provided by this measure applied to the absolute values of the array, to measure dips in magnitude irrespective of sign. Units in the left half typically show negative weights that dip and rise slightly in magnitude, whereas units in the right half typically show positive weights that do the same, and units with a zero x-measure but non-zero y-measure are monotonic but flip sign into the past. Note that the OSR units typically have either negative dip-to-rise weights or positive-to-negative weights, whereas the non-OSR and autoencoding model weights are closer to the origin, indicating that they are less biphasic.

Reviewer Point P 2.4 — There is a recurrent part in the model, but its specific role is not explored. It would be good to have a control where this part is specifically removed.

Reply: We have now further explored the role of the recurrent connections for the spike statistics, the orientation and direction tuning, the texture motion response, the object motion response, the motion anticipation response, and the omission stimulus response. Regarding spike statistics, we have added the following on pg. 4:

"The autoencoding model exhibited similar spike statistics to the prediction model, albeit slightly less aligned to the biology with respect to the spike correlations. Furthermore, the firing rates and spike variability remained intact when we ablated the recurrent connections from the prediction model. This is consistent with the recurrent connections contributing significantly less (6% of total) input current compared to the feedforward connections (Extended Data Fig. 2)."

We have also added on pg. 5-6:

"These elevated correlations persisted when we ablated the recurrent connections in the prediction model (although the peak correlation was slightly decreased)."

Regarding orientation and direction tuning we have added the following on pg. 9:

"Similar to the biology, we found some units to be selective for orientation (16% of units with $OSI > 0.5$) and some selective for direction (4.5% of units with $DSI > 0.5$), with a preference for motion along the horizontal and vertical axes (Fig. 4a). Ablating the recurrent connections slightly increases the number of orientation tuned units to 17% but decreases the number of direction tuned units to 4.2%.

Regarding the texture motion response on pg. 9 we now say:

"Similar to these neurons, we found units in the model (9.1% of units) that did not respond to a static grating of high spatial frequency (for each unit's preferred orientation), yet fired as soon as the grating moved (Fig. 4b). Ablating the recurrent connections reduced the number of units with this property to 8.4%."

Regarding the motion anticipation response on pg. 9 we now say:

"The peak of the anticipation-like response of the model unit population depended to a degree on the recurrent connections, their ablation resulting in it leading the moving bar by a shorter distance (Extended Data Fig. 10). This suggests that gap junctions and/or amacrine cells between RGCs may play a role in motion anticipation."

"Regarding the omission stimulus response on pg. 11 we now say:

"Furthermore, when we ablated the recurrent connections, the OSRs were still present and for the population of units, the dependence of the OSR timing on the period was little altered except for the high frequency sequences (at 20 Hz). ... Together this suggests that OSRs in the model are largely a consequence of the biphasically-sculpted input current interacting with the spiking mechanisms, with a lesser role for the recurrent connections at high frequencies. This is consistent with experimental results suggesting that inhibition from amacrine cells is not needed for OSRs at 12 Hz, but ON bipolar cells are (Schwartz et al., 2008). The importance of the spiking mechanism, and the recurrent connections at high frequencies, for this phenomenon are predictions of the model."

See our response to Point 2.6 for more detail.

Reviewer Point P 2.5 — Another topic which seems overlooked is the metabolic constraint: how important is it ? How robust are the results to this ? Previous works (e.g. Chalk et al, PNAS 2018) has shown that sparseness constraint can play a role when comparing prediction of the future vs compression of the past.

Reply: The metabolic constraint is important in obtaining our results. We found that excluding the constraint renders the model's RFs to lose their oval-like shape and be less aligned to the biology. Importantly, we used the same metabolic regularization for the predictive and autoencoding model for a fairer comparison between the models. We have added the following text to the Results on pg. 6:

"We also found that the metabolic constraint is important in obtaining the results of the prediction model. Excluding the constraint causes the model's RFs to become broad and indistinct, losing their uni-peaked oval-like shape, which are hence less similar to the recorded RFs (Extended Data Fig. 7)"

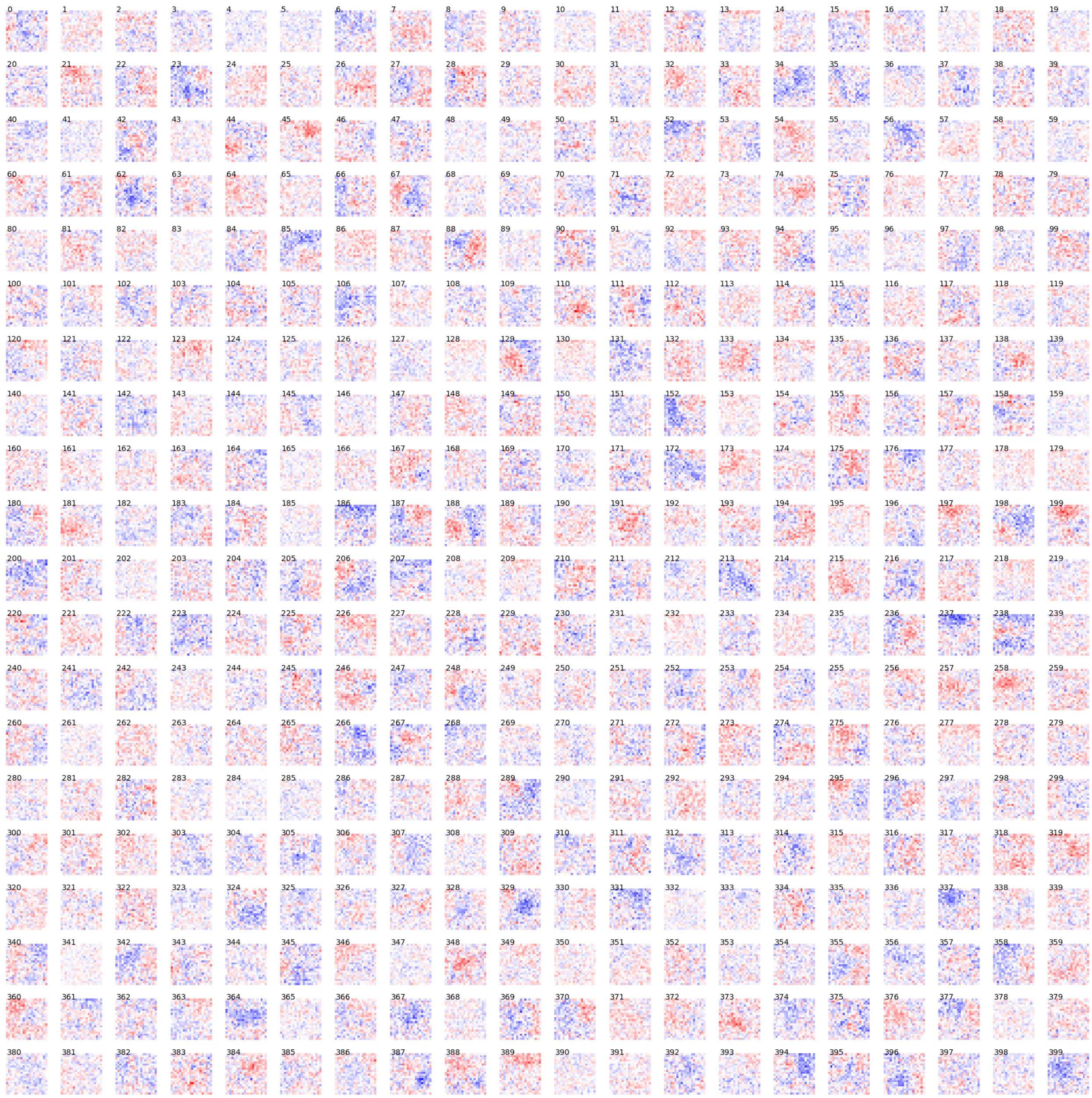

Extended Data Fig. 7: Prediction model spatial RFs trained without metabolic regularization. Every square corresponds to each unit's spatial RF with the largest power over time.

Reviewer Point P 2.6 — Quantification of the different properties of the retina The authors go through several phenomena but do not focus on them carefully enough. For example, for the OSR, the initial paper showed the latency of the response changes with the period of the stimulus, by the same amount. This is not tested here, while this is a crucial property of the OSR.

The same could be said about other properties: for motion anticipation, the neural image was reconstructed, and the position of the peak firing rate relative to the moving bar was estimated for several speeds (and shown to be constant).

For the OMS, they compare a local motion vs a global motion, while the original paper showed a random motion that is either the same in the center and the far surround (eliciting no response) vs two different random motions (eliciting a response).

For the mosaics, the distance between ganglion cells of the same type should be quantified: see e.g. Ravi et al, Journal of Neuroscience 2018, for a method to estimate if there is a real tiling. For some of the mosaics shown, tiling is not even clear.

Also, I find surprising that the mosaics produced by the model are not fully covering the visual space, but only a part of it. This can be explained in experiments because not all the cells of one type are recorded, but it is less clear why you should have this in a model.

Reply: We agree that further quantification and analysis of these properties would be beneficial, and have added the following sections. First, to address the timing of the OSR, we have now included new Extended Data Fig. 12 to illustrate the relationship between the OSR latency and stimulus period and have updated the manuscript on pg. 11 as:

"Certain RGCs in the salamander and mouse retina have been reported to produce an omitted-stimulus response (OSR) (Schwartz et al., 2007; 2008). Such neural responses are generally thought to encode prediction or prediction errors (Auksztulewicz et al., 203). RGCs exhibiting OSRs fire in response to a violation in a periodic temporal pattern, such as an omitted dark-flash (brief luminance reduction) within a steady sequence of dark flashes (Schwartz et al. 2007; 2008). We found that some of the model units (16%) produced an OSR to a violation in a periodic sequence dark-flashing at a rate of 12 Hz (see Methods). These units' responses were sustained throughout the dark-flash sequence, but also responded at the end of the sequence, around the expected time of the response to the omitted dark-flash (Fig. 5d). We also found OSRs to be present for slower (8 Hz) and faster (16 Hz and 20 Hz) dark-flash sequences, as well as when dark-flashes were omitted in the middle of the sequence (Extended Data Fig. 11). Interestingly, substantial OSR responses were only present in the model optimized for temporal prediction and not in the model optimized for autoencoding.

For the temporal prediction model, across the population of units, the latency of the OSR peak relative to the peak of the response to the last dark-flash increased with respect to the period of the dark-flash sequence (Extended Data Fig. 12), as has similarly been reported in the biology (Schwartz et al., 2007). Furthermore, when we ablated the recurrent connections, the OSRs were still present and for the population of units, the dependence of the OSR timing on the period was little altered except for the high frequency sequences (at 20 Hz). In contrast, this OSR timing dependence on period seen in the population was greatly weakened when it was examined for the feedforward input current alone. This suggests a role for the integrate-and-fire mechanism in the OSR. Inspection of the input weights of the OSR units revealed them to often have a biphasic temporal profile - with the net weighting changing from positive to negative as the weight extend into the past, or with decreasing negativity and then a slight increase (Extended Data Fig. 13). This biphasic tendency was also reflected in their receptive fields (Extended Data Fig. 14). We observed these biphasic properties much less in the autoencoding model units and in predictive model units that did not produce OSRs. We confirmed these differences

between OSR and non-OSR units with a quantification of the biphasic character of the input weights (Extended Data Fig. 15), although there was considerable overlap between OSR and non-OSR units. Together, this suggests that the OSRs in the model are largely a consequence of the biphasically-sculpted input current interacting with the spiking mechanisms, with a lesser role for the recurrent connections at high frequencies. This is consistent with experimental results suggesting that inhibition from amacrine cells is not needed for OSRs at 12 Hz, but that ON bipolar cells are needed (Schwartz et al., 2008). The importance of the spiking mechanism, and the recurrent connections at high frequencies, for this phenomenon are predictions of the model."

We have also added the following Extended Data Figure:

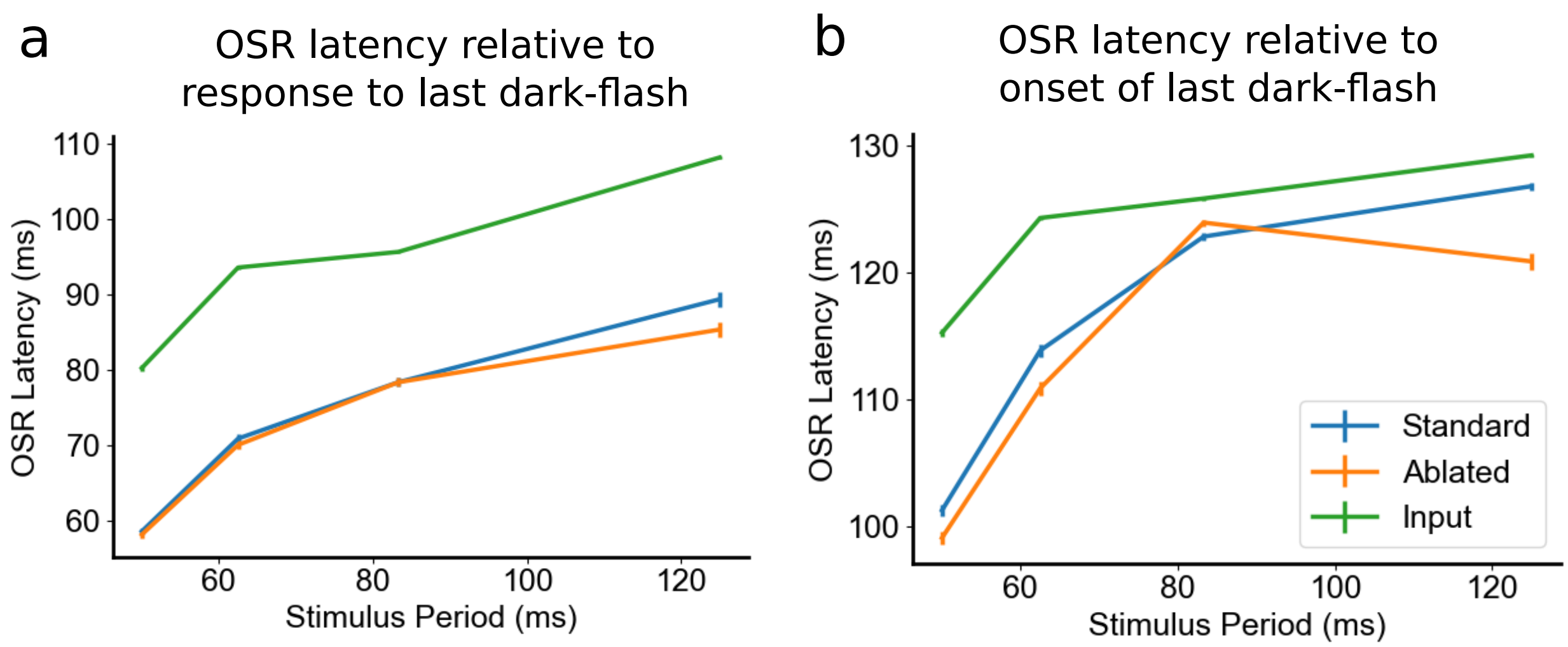

Extended data Fig. 12: Dependence of OSR latency on the flash sequence period. Plots show average latency over all units in the population that show an omission response for that stimulus rate. Blue is the latency measured using the firing rate in the standard model, orange is the latency measured using the firing rate in the recurrency ablated model, and green is the value measured in the feedforward input current. **a.** OSR latency measured from the peak of the response to the peak of the OSR. **b.** OSR latency measured from the onset of the last flash to the peak of the OSR.

We have added the following to the Methods (pg. 23):

"RGCs exhibit OSRs at the end of a dark-flash sequence around the expected time of the next dark-flash. To determine if a unit showed an OSR, we first found the prominent peaks in its PSTH to an OSR stimulus (Fig. 5d). Then we found any peaks that came after the onset of the last dark-flash and if there were at least two such following peaks we took this as an OSR unit. The first of these peaks was taken as the response to the last dark flash, and second one was taken as the OSR."

We have now examined the motion anticipation results in more depth. We have added the following text (pg. 9):

"Certain RGCs have an anticipation-like response to moving objects (such as a moving bar), where

the neuron fires even before the object crosses the neuron's RF, whereas there is a delayed response to simply flashing the bar onto the RF (Berry et al. 1999). This phenomenon is most prominent in transient OFF-type units. When the spatial profile of the population response of such units is examined along the axis of motion, it is ahead of the population response of a coincident flashed bar. Indeed, the peak of the population response also tends to be equal to or ahead of the leading edge of the moving bar by an approximately constant distance, except at high speeds. Similarly, we found certain units in our model to also have an anticipation-like response to a moving bar (Fig. 4d). Furthermore, we found similar population response properties in the OFF-type parasol-like units of the model (Extended Data Fig. 10), although given the small number of such units these results are tentative. The peak of the anticipation-like response of the model unit population depended to a degree on the recurrent connections, their ablation resulting in it leading the moving bar by a shorter distance (Extended Data Fig. 10). This suggests that gap junctions and/or amacrine cells between RGCs may play a role in motion anticipation."

We have also added the following Extended Data Figure:

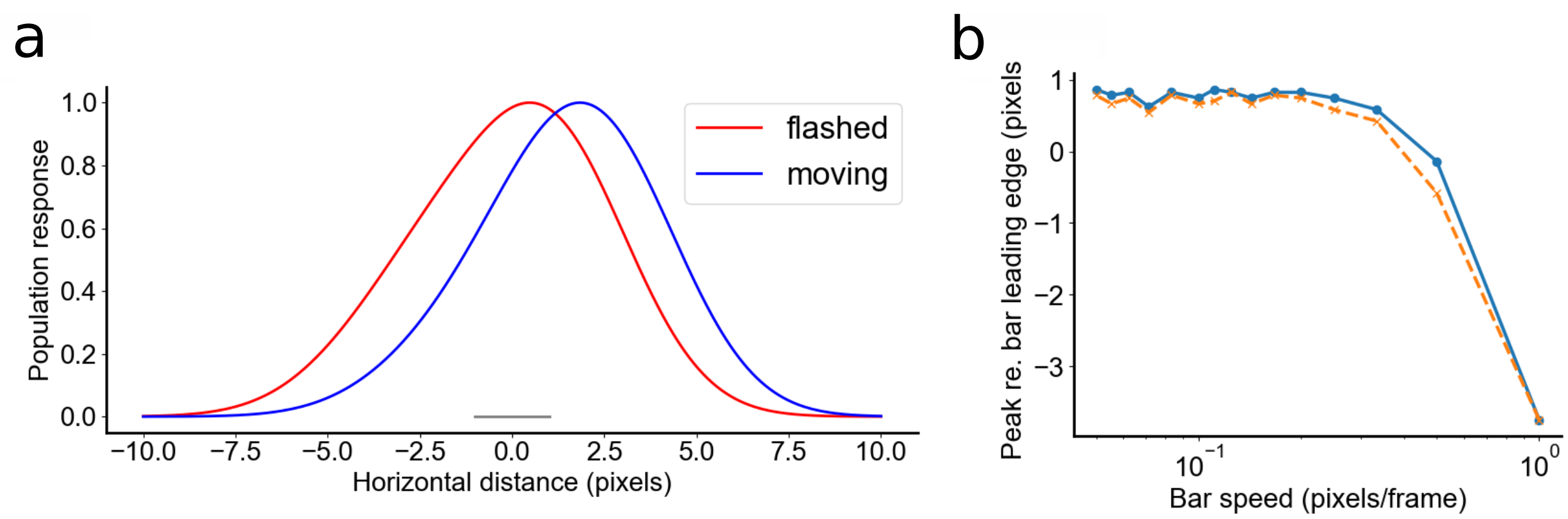

Extended Data Fig. 10: Anticipation-like population responses. a. The population response (blue line) of the OFF-type parasol-like units of the prediction model to a rightward moving bar as a function of the horizontal spatial position of their receptive fields, at the time when the bar first arrives at the position of the flashed bar (gray line). The population response (red line) at the onset time of the flashed bar. The population response was measured as firing rate of each unit as a function of it spatial position relative to the bar, for a moving or flashed bar at 4 different horizontal positions (horizontal pixel positions 11-14). Then the responses were smoothed over space with a Gaussian with a 2 pixel standard deviation. **b.** The position of the peak of the population response to the moving bar relative to the leading edge of the bar, as a function of the bar speed, with (orange dashed line) and without (blue solid line) the recurrent connections ablated.

We have now implemented the OMS with random motion that is either the same or different between the center and the far surround, and investigated the model results to this stimulus in more depth. We have updated the manuscript on pg. 9 to read:

"Some RGCs are object-motion-sensitive (OMS) cells that exhibit differential motion selectivity. This is where a neuron remains silent when an image (or grating) moves across the retina yet fires if the motion in the RF center differs from that in the wider surround (Kuhn et al., 2016; Lettvin et al.,

1959; Olveczky et al., 2003; Baccus et al., 2008), such when the center motion differs from that of the surround or the surround is masked. We also found such OMS tuning in some of the model units for both masked (Fig. 4c) and differential surrounds (Extended Data Fig. 9). We measured the OMS tuning of the population for the differential surround stimulus - the mean object motion sensitivity index (Kuhn & Gollisch, 2016) was positive at 0.11, indicating net sensitivity to differential motion, and this was somewhat reduced to 0.10 when the recurrency was ablated ($p = 3.9 \times 10^{-3}$, Wilcoxon signed-rank test). This is consistent with results suggesting a role for amacrine cells in this phenomenon (Kuhn & Gollisch, 2016; Lettvin et al., 1959; Olveczky et al., 2003; Baccus et al., 2008)."

We have also added the following Extended Data Figure:

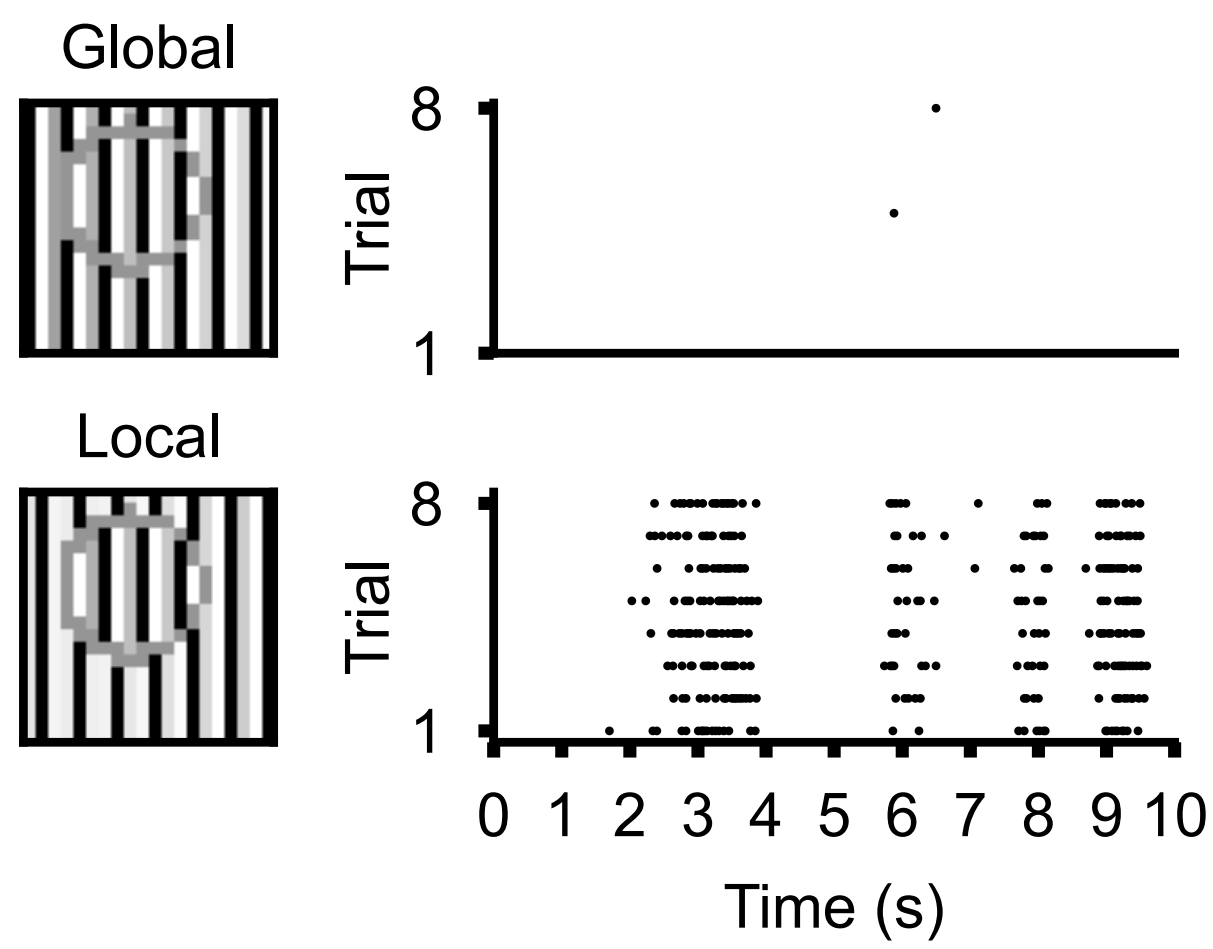

Extended Data Fig. 9: Object-motion sensitivity. An example unit, with coherent jitter between the center and surround ('global', top) and differential (independent) jitter between the center and surround ('local', bottom).

We have added the following to the Methods (pg. 25):

"We implemented two types of object motion stimulus pairs. In the first type (Figure 4c) the stimulus was either a global smoothly moving grating, or the same grating moving only in small circular area centered on the RF of the relevant unit but masked with gray outside the area. In the second type (Extended Data Fig. 9) the stimulus had the same grating moving with random jittered motion both inside and outside the circular area, barring a thin gray annulus, but the random movement was either the same (coherent) or independent (differential) between the inside and outside of the area. For the second stimulus type, we quantified the object-motion-sensitivity of each unit using the object motion sensitivity index; this is given by $(f_d - f_c)/(f_d + f_c)$, where f_d is the firing rate for the differential stimulus and f_c is the firing rate to the coherent stimulus. To calculate the index, we average over 10 different pairs of the stimuli, each pair using different random seeds and of 200 s duration. We then averaged over units."

We have also examined the tiling of the cell types in more detail. We have added the following to the Results (pg. 6):

"The degree of overlap between OFF-type midget-like units better resembles the biology (Ravi et al., 2018) than do the other unit types, which were more overlapping or widely spaced (Extended Data Fig. 5)"

We have also added the following Extended Data Figure:

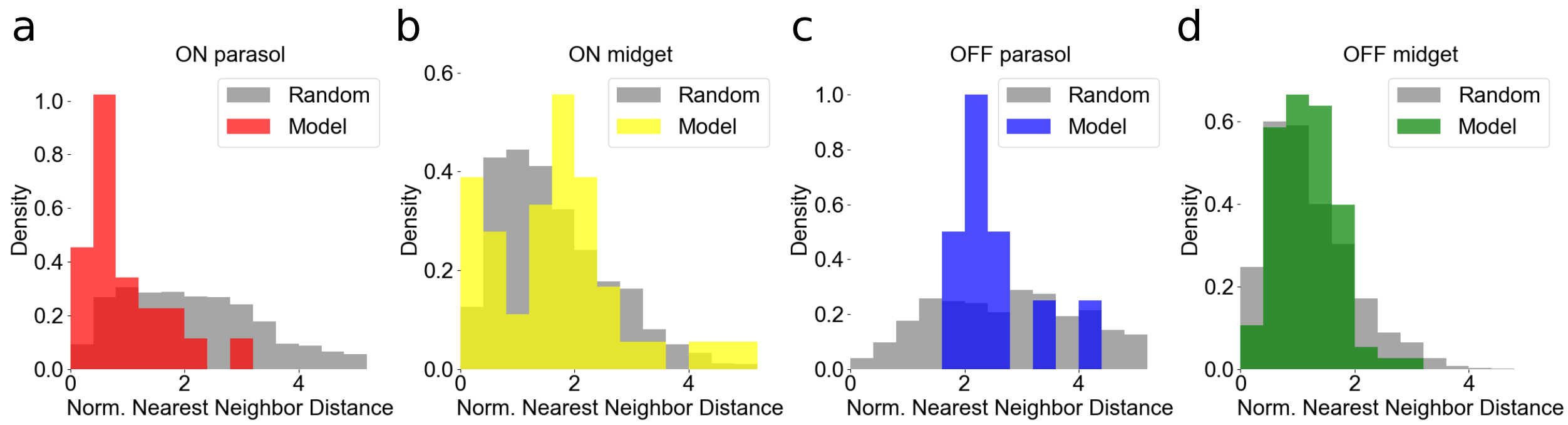

Extended Data Fig. 5: Mosaic tiling. We assessed the tiling overlap of the spatial receptive fields of the predictive model unit using the mosaic tiling measure known as the normalized nearest neighbor distance (Ravi et al., 2018); a low value means more overlap and a high value means less overlap. In the retina, the different cell types each show a peak in the distribution of this measure below about two, with clear space between the peak and zero, and have measure distributions that differ from that of a RGC population with randomly drawn locations. This figure shows the distributions of the measure from the predictive model for the textbfa. ON-type parasol-like units, **b.** ON-type midget-like units, **c.** OFF-type parasol-like units and textbfd. OFF-type midget-like units. The OFF-type midget-like units show a distribution resembling that seen in real RGCs. The rest of the units either overlap more (ON parasol, ON midget) or less (OFF parasol) than the biology. However, consistent with the real RGCs, all the cell-type-like populations, with the exception of the OFF-parasol-like units, which are a very small population, are tiled significantly differently than if their positions were just drawn randomly from that population ("Random" distribution in gray, made using 1000 iterations), as checked with a KS test between "Model" distribution and "Random" distribution (ON parasol, $p = 1, 7 \times 10^{-4}$; ON midget, $p = 9.6 \times 10^{-3}$, OFF parasol, $p = 0.11$; OFF midget, $p = 1.1 \times 10^{-17}$).

We have added the following to the Methods on pg. 24:

"We assessed the character of each cell-type mosaic by measuring its normalized nearest neighbor distribution (NNND) (Ravi et al, 2018). This is given by $2R/(S1+S2)$, where R is the distance between the center of the RF of a RGC and that of its nearest neighbor, and $S1$ and $S2$ are the standard deviations of their respective 2D Gaussian fits upon the straight line joining their centers. When the RFs touch at their standard deviation contours then the NNND is two, if the contours overlap it is less than two, and if they do not touch it is greater than two."

We now also state in the Discussion on pg. 17:

"Finally, while each cell type produced by our model does approximately cover visual space, this mosaic tiling is imperfect with noticeable gaps and overlaps that are not so apparent in the biology. Such tiling has been better captured by some efficient coding models (Jun et al., 2021; 2022; Karlin & Simoncelli 2011; Royt et al., 2021). Nevertheless, that we do see tiling to some degree despite our model objective focusing on temporal rather than spatial aspects is notable. Training the temporal prediction model with more units or with additional efficiency constraints, such as on physical space, may enable more even and well-spaced tiling."

Reviewer 3

In this work, Taylor et al. set out to compare two highly influential perspectives on retinal coding using a novel spiking model. The first perspective, efficient coding, posits that early sensory circuits maximize encoding of information about the stream of incoming natural inputs. The second perspective, predictive coding, argues that these same systems instead extract information most relevant for predicting the future. By training their network to maximize linear decodability of stimuli several time steps into the future, they demonstrate agreement with a variety of previously observed retinal phenomena, including differing cell types, motion tuning, stimulus omission responses, and prediction of retinal firing. They argue that these results give strong support to the predictive coding model and against efficient coding.

There are many things to like about the paper. In particular: - The work is clearly explained and results well presented. - The use of both a spiking model and recurrence are strong positives, since these are more accurate than most rate-based models that have been used in the field. Similarly, the accounting of both graded potential and spike costs in the model makes sense biophysically, as both require energy. - The authors consider a variety of experimental findings to which they match their model, presenting not just one line of argument but several. It is the cumulative force of these results that is the strength of the paper.

However, I have several concerns and questions that I would like to see addressed:

Reply: We thank the reviewer for their positive assessment, and we hope that our answers below satisfy their comments.

Reviewer Point P 3.1 — **Comparison with efficient coding:** While the framing of the paper is in terms of a test of efficient versus predictive coding, the authors mostly argue past efficient coding, focusing on phenomena explained by their model. Granted, it would be quite difficult to argue that efficient coding *cannot* account for some particular phenomenon, but the only direct comparison I can see between the two approaches is in the analysis of Figure 6 (predicting retinal responses), which compares an "encoding" model with a "prediction" model. I assume the "encoding" model is the authors' own model predicting T = 0 steps into the future, but I couldn't seem to verify this in either the text or methods. Given the authors' framing, I would have expected to see multiple comparisons between efficient and predictive coding approaches throughout the manuscript, but in the event, this formed only a minor part of the paper.

Reply: The reviewer is correct that the "encoding" model is the model but predicting T=0. Note, we are now calling the "encoding" model the "autoencoding" model to avoid confusion with descriptive encoding models that are fitted to data. We have now clarified this in the text on pg. 4:

"Apart from the different prediction offsets T_{off}, the autoencoding and prediction models had exactly the same architectures."

In addition to our retinal response prediction results - which we believe to be the most pertinent in our argument in favor of prediction over efficient encoding - we have now incorporated other comparisons between between the models throughout the text:

1. We now compare the firing rates and spiking variability of RGCs to that of both the predictive and autoencoding models (updated Fig. 2c and d). On pg. 4 we now say: "The autoencoding model exhibited similar spike statistics to the prediction model, albeit slightly less aligned to the biology with respect to the CV(ISI)."

2. The predictive model exhibits more realistic correlations between spike trains compared to the autoencoding model (new addition to Fig. 2c and d), with the autoencoding model over-estimating the correlations at lag equal to zero. On pg. 5 we now say:

   "We measured the correlation between the spike trains of different units by calculating the spike train cross-correlogram, which assesses the correlation between randomly selected pairs of units at different time lags. For pairs of RGCs, the peak correlation is at time lag zero for the image (Fig. 2c) and movie datasets (Fig. 2d). A peak at zero was also seen for the prediction and autoencoding models, with the peak for the prediction model being closer to that of the biology. The spike train correlations symmetrically decayed for increasing and decreasing lag for both models, as with the biology. However, for the movie dataset, both the prediction and autoencoding model units tended to be more correlated for non-zero lags compared to mouse RGCs."

   We have now also updated the caption of Figure 6d to read: "**d.** Neuron prediction scores of the best predictive model (y-axis, optimal offset into the future) against the autoencoding model (x-axis, 0ms into the future) across the different datasets (with the red dot plotting the centroid)."

3. We found that the spiking autoencoding model does not exhibit the large parasol-like spatial RFs (new Extended Data Fig. 4). Additionally, and unlike the biology, many of the RFs are not uniquely localized and oval shaped, but rather display power in various regions of the frame. On pg. 6 we now say: "The RGC-like RF tuning properties were less evident in the autoencoding model in which we found the parasol-cell like RFs to be missing (Extended Data Fig. 6)." See our response to Reviewer point P1.6 for the figures.

4. We did not find any substantial OSRs in the autoencoding model units (see section "Emergence of a relative spike latency code and stimulus omission responses"). On pg. 11 we say: "Interestingly, substantial OSR responses were only present in the model optimized for temporal prediction and not in the model optimized for autoencoding."

Reviewer Point P 3.2 — **Future prediction, latency, and information:** While the authors cite Palmer and coauthors multiple times in favor of the predictive coding capacity of the retina, their own formulation of the coding problem is somewhat different in ways that may or may not be important. More specifically, Palmer et al. (and Chalk, Marre, and Tkacik, 2017, which I was surprised not to see cited) consider the predictive information about the *entire future* given the *entire past*. This is somewhat different than the authors' formulation, which is attempting to predict a *specific point* in the future from current neural activity. I recognize that this is necessitated in part by the need for a tractable training objective, but the assumption of a specific latency for which prediction should be optimized seems fairly ad hoc and an important theoretical caveat in the paper. While the authors do cite Palmer et al. (ll.564-66) for the 50-100 ms time frame, I believe that number refers to the *maximum* time over which predictive information was present in that study (at least if the reference is to Fig. 3C in that work). That is, if I recall correctly, predictive information was highest for the immediate future and fell off quickly in time. It would strengthen the paper if either some theoretical argument could be made by the authors that this specific prediction time lag is the right one or they could show that a model that maximized predictions over the *entire* future gave the same results.

Reply: We have now cited Chalk et al. (2017) in a new paragraph the Discussion. We did cite this work in our earlier temporal prediction papers, and it was an oversight on our part to not cite this important paper here. For discussion on the relation of our work to Chalk et al. (2017) see Reviewer Point P 4.1.

We agree that our formulation is different from that in Palmer et al. (2015) in a number of ways, including that we use linearly decodable information and aim to predict a single time point. We also think that our wording regarding the comparison to Palmer et al. (2015) was clumsy. We simply wished to note that the timescales involved in both studies were of a similar order of magnitude.

Figure 3C in Palmer et al. (2015) measures the predictive information retinal responses have about the entire future of a one-dimensional artificial Brownian motion stimulus. For this stimulus, unlike for natural stimuli, the predictive information about the entire future can be determined using just the two immediate future time-points, due to its Markovian structure. We expect that for our model with natural stimuli, this predictive information would fall away from the present in a similar manner, given the temporally smooth nature of natural stimuli, but calculating this would be intractable because of the high-dimensionality of our model and input. In contrast our Figure 6c measures how well the model captures retinal responses as a function of distance into the future predicted, a quite different measure, which we would not at all expect to follow the same form as Figure 3C of Palmer et al. but that we might suspect would operate over similar timescales.

We have now reworded the relevant paragraph on pg. 15, and expanded upon it to discuss why the specific time lag is relevant:

"It is interesting to speculate on why this particular timescale might be important. One reason could be that an organism needs to know the likely state of the world at the time of future immediate action, for example to catch prey or avoid predators. This would be a fraction of a second into the future, where shorter or longer timescales may be less effective for this role. This timescale could account for transduction delays, which are on the order of tens of milliseconds at the photoreceptor (Schnapf et al. 1987; Donner, 2021), as well as potentially transmission delays and movement delays for very fast motor responses (Bixler, 1967)."

"This timescale is also in some respects consistent with a prior study (Palmer et al. 2015), which found that for naturalistic stimuli the retina carries predictive information about its own activity out to hundreds of milliseconds. This study did not involve a normative model and used different measures: the mutual information in the retinal response about the full future of simple one-dimensional Brownian-motion stimuli, and information about future retinal responses in the current retinal responses for naturalistic stimuli."

As the reviewer correctly surmises, we chose our objective to be tractable and scalable, as well as decodable by upstream neurons. Training to predict the entire future, particularly for the full mutual information, would not be computationally feasible. However, we have in the past predicted a limited span of the future rather than a single timepoint for our non-spiking temporal prediction models (Singer et al. 2018). We found that doing this did not alter the results much when the span covered a range of values around that timepoint.

We think the similarities and differences between our approach and Palmer's are very interesting. Indeed, we have an ongoing collaboration looking into this relationship. While our model is optimized to predict a particular timepoint, we expect that the model response would still be informative about a span of future timepoints focused on the immediate future. We hope to expand on this more in future work.

Reviewer Point P 3.3 — **Understanding model ingredients and limitations:** While the bulk

of the paper is (rightly) devoted to the successes of the authors' model, I found discussion of some of its limitations lacking. For instance, it would be good to know which of the experiments the authors report rely critically on future prediction and which simply rely on the presence of recurrence in the model. Given the degree of novelty in the model ablation studies that help pinpoint the role of spikes, recurrence, and prediction time lag would be helpful. One cannot ask them to repeat these experiments for all possible combinations, but I would appreciate more clarity on which innovations are most important for explaining their results.

Reply: As requested, we now include further ablations and comparisons.

1. Recurrency: We further explored the role of the recurrent connections and found that they contribute significantly less (6% of total) input current compared to the feedforward connections post model training (new Extended Data Fig. 2). Furthermore, ablating the recurrent connections in the predictive model did not significantly alter the model's spike statistics to natural images and movies (updated Fig. 2c and d). Similarly, this did not alter the fraction of units exhibiting an OSR (16% in both cases) at 12Hz, but did impact the timing of the OSR at high frequencies. Ablating the recurrent connections slightly increased the number of orientation tuned units and slightly decreased the number of direction turned units and units showing Y-type cell-like responses. It also reduced the object motion sensitivity of the population, and slowed anticipation responses. For the changes we made to the text on this, see our response to Reviewer points P2.4 and P2.6.
2. Spiking: The inclusion of the spike mechanism allowed us to conduct our latency analysis (Figure. 5a, b, c), which is otherwise not possible in non-spiking models. We also found that the spiking mechanism was useful in having better dependence of the omission response on the dark-flash rate. See our response to reviewer points P2.6 and P4.2.
3. Prediction time lag: See our response to P3.1.
4. Metabolic cost: We now include a figure illustrating the spatial RFs of the predictive model trained without a metabolic regularization constraint (Extended Figure 7). These RFs do not match the biology, providing further evidence that the retina balances sensory processing under specific energy constraints. See our response to point P2.5.

Reviewer Point P 3.4 — Along different lines, there are at least two respects in which the model appears to perform poorly in comparison with efficient coding models. The first is mentioned by the authors themselves: receptive fields in their model lack surrounds. Despite the fact that some weak surrounds have been reported in some preps, it is striking that their model does not reproduce one of the most salient features of retinal physiology, one that many efficient coding models successfully reproduce. Second, the mosaics produced by the model (Fig. 3D) are not well-formed. For example, the ON parasol cells identified in the model have substantial overlap between neighbors, as do the OFF midget cells. This is a degeneracy that may have only a mild effect in reducing predictions from the model, but it is one that is well removed by several of the efficient coding models the authors cite.

Reply: We have now found that if we use more noise samples to measure the spatiotemporal receptive fields of our model in greater detail, then surrounds are in fact evident for many of the units of the prediction model. These surrounds tend to be lagged relative to the temporal peak of the centers,

consistent with the biology. For more details, please see our response to point P1.7 and our new Extended Data Figure 8.

We have now quantified the mosaic tiling using the mosaic tiling measure of Ravi et al. (2018). At least by this measure, the degree of overlap of the OFF midget cell receptive fields is consistent with the biology, but the ON parasol and ON midget cells overlap somewhat more than the biology and OFF parasol cells somewhat less. For more details, see our response to point P2.6, where we provide the new Discussion text mentioning the efficient coding models in this context and the new Extended Data Figure 5.

Reviewer Point P 3.5 — To clarify: I do not think it is important for models to get everything right, but it is likewise important here to notice what they get wrong. What the authors have provided is a compelling case that their model reproduces some interesting phenomena, but it is likewise clear that the model does a poorer job of explaining some features that efficient coding does well.

Reply: We thank the reviewer and agree with their point. We have endeavored to point out the current weaknesses of the model more explicitly, for example, that it could do better at capturing mosaic tiling. This is illustrated, for example, by the new manuscript text provided in response to the reviewer points P1.3 and P2.6.

Minor comments

Reviewer Point P 3.6 — Why does metabolic cost depend on synaptic weight, exactly in (10)? Isn't the majority of energy usage just proportional to the total number of action potentials, not the input currents? Some further explanation here would be helpful.

Reply: Recent work (Faria-Pereira & Morais 2022, Harris et al. 2012) suggests that maintenance of resting potentials in response to synaptic transmission is the main energy demand of the brain (43%), followed by putative housekeeping processes (25%), then action potential propagation (17%), then resting potential maintenence (16%). We have now added the following to the Methods on pg. 20:

"Synaptic transmission has been noted to require significant energy within the cerebral cortex, alongside spiking and maintenance of resting potentials (Faria-Pereira & Morais 2022, Harris et al. 2012). Hence, our loss function encapsulates both the energy expenditure of resting potential maintenance and the interaction of spikes and synapses."

Reviewer 3 (Remarks on code availability)

Reviewer Point P 3.7 — I attempted to review the code, but the repository has not been made public.

Reply: Apologies for this, the code is now public.

Reviewer 4

In this study, Taylor and colleagues constrain a neural network model of the retina to encode predictive information and find that the model recapitulates several known properties of the retina. Overall, the study is interesting and has potentially important results. However, the study does not adequately reference the previous literature and does not provide a deeper mechanistic understanding of predictive processing. Outlined below are several points that should be addressed prior to publication.

Reply: We thank the reviewer for their assessment and interest, and we hope that our answers below satisfy their comments.

Reviewer Point P 4.1 — Two previous studies have developed normative approaches to understand how prediction affects the receptive-field structure of sensory neurons. The authors cite the Singer et al paper, but the second paper is not cited (Matthew Chalk, Olivier Marre, Gašper Tkačik, 2018 PNAS DOI: 10.1073/pnas.1711114115). Besides missing a key study (in my opinion, THE most important study so far), the issue is that the Chalk et al paper covers the same ground as the current study. In other words, the current study does not build on the previous literature, which makes it difficult to understand how the current study is situated relative to previous work and to ascertain how it contributes to our understanding of predictive coding.

Reply: We agree that Chalk et al. (2018) is an important study and we now cite their work in our manuscript. While there is some relationship between the studies as they both involve temporal prediction, the differences are substantial:

1. We explored temporal prediction versus efficient coding in the retina. Chalk et al. (2018) explored sensory coding without focusing on a particular sensory region (their results more closely resemble coding in primary visual cortex rather than the retina).
2. We used a spiking neural network with recurrent connectivity for our normative investigation. In contrast to our non-linear model, Chalk et al. (2018) employed linear models. This is likely a major reason why our results are substantially different from the Chalk et al. (2018) study. The possibilities opened up by non-linear models are non-trivial.
3. When performing temporal prediction Chalk et al. (2018) did not find anywhere near the diversity of response properties that we found; for example, they found no direction-selective units.
4. We regularized our network by minimizing synaptic activity, whereas Chalk et al. (2018) regularized by a sparsity constraint on unit responses.
5. Chalk et al. (2018) used a patch sliding over images as input, whereas we used dynamic movies of natural scenes.
6. Due to the simpler objective and optimization process of our model, it is more scalable, enabling testing against diverse properties of the brain.
7. We extensively contrasted our model's spiking statistics, receptive fields, motion-tuning properties, and latency-coding properties to the biology. Chalk et al. (2018) primarily focused on analyzing just the linear receptive fields in their model for a limited comparison to the biology. Indeed, as

they use a simple linear model, rather than a non-linear recurrent spiking model, they would not have been able to conduct many of our analyses.

8. We tested how well our model trained for different prediction spans into the future could quantitatively predict actual RGC responses to natural images and movies across different animal species, to ascertain whether temporal prediction or efficient coding better captures the responses. Chalk et al. (2018) did not perform a quantitative assessment such as this.

We agree with the reviewer that the approach used by Chalk et al. (2018) is very valuable and has interesting similarities and differences to our model. However, our study examines whether diverse retinal phenomena can be accounted for within the theoretical framework of temporal prediction, rather than an in-depth exploration of a very simplified model. However, we believe the two studies complement each other, despite using very different models (e.g linear non-spiking vs nonlinear spiking encoder) and having different aims. We have now added the following paragraph to the Discussion on pg. 15:

"Another study (Chalk et al., 2018) maximized the predictive information of a non-spiking linear model about naturalistic visual input at future time-points. These authors observed units with receptive fields that resembled the simple cells of primary visual cortex, but without direction selectivity. In contrast, our model units had retina-like receptive fields and captured many diverse characteristics of RGCs, including some with direction-selectivity. These differences are likely to arise because our neural model is non-linear, uses strong synaptic activity constraints, and is trained on movies rather than shifted image-patches."

Reviewer Point P 4.2 — Another issue is that the paper presents properties of the model that are consistent with retinal processing (e.g., omitted stimulus response), but there is no tie to the underlying structure of the network model. What we need as a field is a more mechanistic understanding of how particular network structures give rise to functional properties. It seems that the authors would have all of the tools at their disposal to provide this – what is it about the connectivity within the network that gives rise to properties such as OSR? Without this, I think the potential impact of the network modeling is greatly diminished.

Reply: We agree and have now further investigated the relationship between the omitted stimulus responses and our model's underlying structure. These findings suggest that the feedforward connectivity, combined with the spiking non-linearity, play a more dominant role in generating the OSRs than the recurrent connectivity (except at high dark-flash rates). This is consistent with the finding of Schwartz et al. (2008) that blocking amacrine-cell-mediated inhibition did not effect OSR latency in the salamander retina, whereas interfering with ON-bipolar cells did. We also inspected the temporal profiles of the input weights and RFs of the autoencoding and predictive models and found the units with an OSR in the predictive model exhibited biphasic RFs (new Extended Data Figs. 13a and 14a). In comparison, we found that the temporal profiles of the units in the autoencoding model were monophasic (new Extended Data Figs. 13c and 14c), suggesting that biphasic structure in the input weights and RFs is another factor contributing to the OSRs. Hence, a prediction of our model is that while the feedforward input structure to RGC is important for OSR responses and their dark-flash rate dependence, the spiking-nonlinearity is also important.

We have now added new text to pg. 10 on the network characteristics that give rise to OSRs. See our response to points P2.3 and P2.6 for more detail and this text.

Furthermore, we have now explored the roles of the network structure of recurrency in various other retinal phenomena such as spiking statistics, orientation tuning, direction tuning, texture motion tuning,

object motion sensitivity and motion anticipation responses. See our responses to points P2.4 and P2.6 for more detail and associated text changes.

Reviewer Point P 4.3 — It is unclear to me why midget-like and parasol-like (in size) cells would fall out of a model like this. Midget cells are contributing to high spatial resolution and color vision. How is it that these cells would fall out of a normative model for prediction? Furthermore, it is difficult to determine how the constraints in the model relate to the results – e.g. What are the results if the model is constrained to have 1, 2, 4, or more cell types?

Reply: We did not include color in our training data and hence the midget-like units did not emerge in our model to encode color. To predict the visual future requires both spatial and temporal features of the visual input. We speculate that the midget-like units encode slow spatial detail while the parasol-like units encode the more rapid coarse-grained dynamics of the scene. Exactly why two dominant types emerge in the model (or four if ON/OFF differences are included), rather than one, three, four or more or a continuum is a deep question that is beyond the scope of this manuscript. However, we think it is reasonable to conclude that these properties emerge because they are optimal for temporal prediction given the model's constraints. It is worth noting that other normative studies (e.g. Harper & McAlpine 2004) suggest that two or a few discrete neural sub-populations can in certain situations be preferable to a neural continuum for accurate coding when the neural tuning widths are broad relative to the distribution of the stimuli.

The only constraint in our model's training objective is the metabolic regularization term (Eq. 7). We did not constrain our model to have a particular number of cell types. Rather, all cell types emerged within the model from training for temporal prediction with the metabolic regularization constraint. To make this more clear, we have added the following sentence to pg. 6 of the Results:

"After training for temporal prediction, the model units, which before training were undifferentiated, appeared to separate into different functional types."

Excluding this metabolic constraint led to less defined receptive fields without a clear parasol and midget distinction, suggesting a key factor in driving this distinction is metabolic efficiency. Also, training the model to encode the current stimulus rather than predict the future stimulus led to loss of the OFF parasol unit types. Together, this suggests that a constraint for metabolic efficiency and an objective of prediction are both required to drive the emergence of the midget/parasol division. See our response to P2.5 and P3.1 for more detail and the text changes.

Reviewer Point P 4.4 — I attempted to review the code for this paper using the link provided, but the code was not available. GitHub gave me an error message that the page did not exist. The code for this manuscript needs to be reviewed carefully prior to publication.

Reply: Apologies for this, the code is now public.

Minor comments

Reviewer Point P 4.5 — Abstract: "Our findings demonstrate that the retina is not merely a compressor of visual input…" I suggest softening this claim. The evidence for this is based solely on a computational model, no new empirical evidence is provided in this study, so "demonstrate" is much too strong a word. The findings are "consistent" with the idea that the retina is not a simple compressor of visual input.

Reply: We agree and have replaced "demonstrate" with "suggest".

Reviewer Point P 4.6 — Several aspects in the first paragraph of the introduction are difficult for me. First, when the authors state that the retina "sends a representation of the visual world to the brain" (lines 49-50). There are more than 20 distinct output (ganglion cell) pathways in the vertebrate retina and it is thought that each of these pathways provides a distinct representation of the visual world.

Second, lines 50-51: "Spatially downsampled by about 100 times." This depends on cell type and species. In the primate macula, the cone signals are actually upsampled by a factor of two by the midget system.

Third, "It remains a open question what information this pattern of activity represents" (lines 51-52). I think this is also a very inaccurate statement. There has been a tremendous amount of progress understanding what these pathways represent – we likely know more about how information is represented in the retina than any other part of the brain. The literature is packed with primary studies and reviews on this subject.

Reply: Taking all this on board, the start of the Introduction on pg. 2 now reads:

"Retinal ganglion cells (RGCs), driven by the photoreceptors via bipolar cells, respond to different patterns of light (Hartline 1938, Kuffler 1953) and send representations of the visual world to the brain (Meister and Berry 1999). Despite extensive progress, it remains an open question what general principle, if any, governs the information this collective pattern of activity represents."

Reviewer Point P 4.7 — Fourth, the phrase, "like a crystal ball foretelling the future" (lines 55-56) is too colloquial for a scientific journal, in my opinion. I would consider rephrasing.

Reply: We acknowledge your feedback and have removed this.

Reviewer Point P 4.8 — Lines 298-302: The authors cite an example of a cell in their model that did not respond to stationary presentations of high spatial frequency gratings, but responded when the gratings moved. They then state that this cell is similar to Y-type cells in mammalian retina. However, Y cells DO respond to flashed gratings (see original Hochstein and Shapley paper [1976] as well as the significant subsequent literature [e.g., Demb et al. 2001]). These cells are defined by their nonlinear spatial summation because they have nonlinear/rectified subunits. In fact, it is puzzling how one could obtain the result described in the present work in which a cell does not respond to a flashed high spatial frequency grating, but responds to a moving grating of the same spatial frequency. This is very non-retina-like. It would be helpful for the authors to describe how this property might arise.

Reply: To clarify, we did not flash the gratings but rather inputted the static gratings, unchanging over time or space, for a prolonged period of time (3 s). Our model would also exhibit nonlinear spatial summation in its units due to the recurrent connectivity. We now use the word 'static' rather than 'stationary' in the relevant sentence to be more clear.

Reviewer 4 (Remarks on code availability)

Reviewer Point P 4.9 — The code was not available from the URL provided by the authors. I received an error on GitHub when I attempted to access the code.

Reply: Apologies for this, the code is now public.

NCOMMS-24-22503A: Temporal prediction captures retinal spiking responses across animal species

Response to reviewers' comments

Comments in black. Replies in blue. Manuscript edits in red.

Reviewer 1

The authors added substantial amounts of data to explain results that were previously unclear or ambiguous. Addition of extended data figures now highlight the details of the RFs predicted by the models, distinguishing subtle spatial and temporal variations that enable encoding of distinct stimulus features. Although, the data and expanded text in the manuscript satisfy most of my comments and comments by other reviewers, there are small details that still need attention:

Reply: We thank the reviewer for their positive assessment, and we hope that our answers below satisfy their comments.

Reviewer Point P 1.1 — 1. Some of the results are described without explicit mention of the species the data are sourced from. Because spiking dynamics, latency, and RF properties can vary significantly across RGC types between species, it is necessary to clarify the species when presenting data. For example, in Figure 4, it is unclear whether the OS and DS cells are from salamander or mouse or rabbit. DS and OS cells are rare in primates, compared to rodents; hence making this distinction clear both in figure and text across the manuscript, is important.

Reply: Apologies for not making this more clear. The example units in Figure 4a are units from the prediction model. We have now clarified this in the caption text:

"Right: sample tuning curves of an orientation-selective model unit and a direction-selective model unit."

We have now gone through the manuscript and ensured that wherever we present experimental data we clearly state the species from which they were obtained. We have also added this information to the Discussion where this wasn't already the case.

Reviewer Point P 1.2 — 2. Although the term 'autoencoding model' is intended to distinguish this framework from traditional encoding models, 'autoencoding' itself is a misnomer, as it may be misconstrued as an auto encoder model, which fall in the category of unsupervised neural networks trained to extract latent structure in data. I suggest using an alternative term such as 'zero-lag encoding model' or 'non-predictive encoding model'.

Reply: We used the term autoencoding model as it is essentially an autoencoder model, which is perhaps the term that we should have used in the first place. This is because the objective of the model is to reconstruct its own immediate input. Although it is a little different from a standard autoencoder in that it has spiking and recurrent aspects, the objective is the same. With the regularization, it is effectively a variant on a sparse autoencoder. Originally, we used the term "encoder model", but we were concerned that this might be confused with the kind of encoding model that is fitted to data, such as an STRF; hence we opted for autoencoding model. However, to avoid any confusion, we are happy with the

reviewer's suggestion to use "non-predictive encoding model", and have now replaced all instances of "autoencoding" with "non-predictive encoding".

Also, to clarify the relationship of this model to an autoencoder, we have added the following sentence to the Methods:

"Note that this dependence of the regularization on the absolute value of the activity means that the case where $T_{\text{off}}^{\text{steps}} = 0$, the non-predictive encoding model, can be seen as a variant of a sparse autoencoder, but with a biologically-inspired sparsity objective and spiking and recurrent properties."

Reviewer Point P 1.3 — 3. Overlaps in RF mosaics are due to possible contamination from cells that may belong to other types (for example, Fig. 3 d, ON-parasol, OFF-midget). Is it because the spiking model with recurrent connections fail to account for the convergence of bipolar cell inputs or noise in data (spike sorting limitations)? Please clarify this in results or discussion.

Reply: These are interesting points to consider. First, our model does not completely account for the convergence of bipolar cell inputs, as evidenced by the overlap of the receptive fields. It is conceivable that this is because we did not include any non-linear properties of the bipolar cells, which may better enable model units to be differentially activated. Although the clusters in Figure 3a are fairly distinct, there could be a degree of misclassification, such as between OFF parasol and OFF midget units. However, while this may be a factor, we suspect it is not the main story. The overlap in RF mosaics could also be partly due to noise in our model and receptive field assessment process or due to limited training data or training time of the model. We have now added the following to the Results:

"As the clusters for the different unit classes are quite distinct (Fig. 3a), this overlap is unlikely to be due to poor cell-type assignment."

We have also added the following to the Discussion:

"However, bipolar cells can exhibit some non-linear integration (Schreyer & Gollish, 2021), thus modeling them with an additional layer of non-linear graded units may enable better tiling by the mosaics."

Reviewer Point P 1.4 — 4. A key aspect of natural viewing is dynamic changes in luminance. Multi-layered models with gain adaptation at the photoreceptor-BC synapse can better account for dynamic changes in luminance (PMID: 39009568), which may be a limitation of the existing framework. It is worth speculating in discussion, whether a predictive framework may (or not) be sufficient to capture natural luminance changes.

Reply: The model does in theory have have the capacity to capture responses to dynamic changes in luminance through its recurrency and non-linearity. Indeed, it has been shown in the auditory system that a single hidden-layer neural network can capture gain adaptation in neurons despite having no explicit gain mechanisms (Harper et al. 2016). The fact that our model performs well in representing RGC responses to dynamic natural stimuli, which can include dynamic changes in luminance, suggests that it might be doing so. However, explicitly adding a photoreceptor gain adaptation mechanism may further improve this. We have now added the following to the Discussion and included Idrees et al., 2024 in the reference list:

"Futhermore, photoreceptors show nonlinear adaptation, which may mechanistically underlie RGC responses to luminance changes in natural stimuli (Idrees et al., 2024). Incorporating an additional nonlinear dynamic layer for the photoreceptors and bipolar cells would allow investigation of whether luminance adaptation is also a functional consequence of temporal prediction."

Reviewer #1 (Remarks on code availability):

Reviewer Point P 1.5 — The GitHub link works and it contains the codes used in the paper. The README file contains sufficient details for downloading, training, and reproducing results

Reply: We are glad to hear this, thank you for checking.

Reviewer 2

Unfortunately, reviewer 2 is unavailable to review this round. We therefore seek further comments from other reviewer(s). *A brief summary of cross-comment for unavailable reviewer 2: '- The findings are interesting, but most of them are not uniquely explained by the authors' model. - There is some evidence to indicate that the authors' autoencoding model is not a strong baseline (e.g., the existence of "nonlocal" RFs in Extended Data 6). - There is at least one case where the authors' model does worse than existing efficient coding models (mosaic formation).'

Reply: A key strength of our model is the breadth of retinal phenomena it can explain under one simple principle: efficient temporal prediction. While different subsets of RGC properties can indeed be explained by other normative models, ours unifies all these properties under one framework and additionally predicts other features such as motion anticipation, stimulus-omission responses and differential motion tuning. Furthermore, our model incorporates recurrent connectivity and spiking, important features of the biology that were not included in previous efficient coding models, which enabled us to demonstrate that spike latency codes can also be accounted for by this principle.

Having said all this, we are not suggesting that aspects of the anatomy and physiology of the retina are inconsistent with other principles. Our study simply demonstrates the remarkably broad explanatory power of temporal prediction for understanding the retina. To clarify this, we have added the following sentence to the Introduction:

"Although other principles, such as efficient compression, may also be factors underpinning the anatomy and physiology of the retina, this study demonstrates the broad explanatory capacity of the principle of temporal prediction, which can account for a remarkably diverse range of RGC properties across different species."

To recognize the limitations in mosaic formation of our model, we have also modified a prior sentence in the Introduction to read:

"Like prior non-spiking normative investigations (Ocko et al., 2018; Jun et al., 2021; 2022; Karklin & Simoncelli, 2011, Roy et al., 2021, Singer et al., 2023), we found that our spiking model optimized for efficient temporal prediction was able to capture retina-like RFs and to a degree their spatial organizations."

We have also expanded a related sentence in the Discussion:

"In addition to capturing the spatial RFs of RGCs, their temporal dynamics, and, to some degree, their mosaic-like spatial organization, the predictive model exhibited several other properties that have been described in a range of vertebrate species, and which have not been accounted for by other normative studies of the retina."

Regarding the autoencoding (efficient compression) model, there are many ways of instantiating efficient coding or efficient compression models. Although our efficient compression model, with $T_{\text{off}}^{\text{steps}} = 0$, may produce some non-local RFs, many of the RFs are well localized. More importantly, this is

an efficient compression model with spiking neurons, a biologically-based restriction not placed on previous efficient coding models, which may explain its limitations. It is possible that a spiking efficient compression model could be modified in ways that better capture certain aspects of the retina, but that is not the focus of our paper. What is important for our efficient compression model is that it is based on exactly the same modelling framework as our temporal prediction model, enabling a fair comparison under the particular constraints that we have used.

We agree that there are limitations in our model, as there are with all models. One particular limitation is that although efficient temporal prediction does produce RGC mosaics, they are not as well captured by our model as they are by some efficient coding models that lack a spiking constraint. It remains to be seen whether future developments of the temporal prediction model or perhaps some combination of efficient coding and temporal prediction can capture the mosaic organization better, in addition to all the RGC response properties.

To this end, we have modified the paragraph where we discuss the limitations of our model to read:

"Finally, although each cell type produced by our model approximately spanned visual space, this mosaic tiling is imperfect with noticeable gaps and overlaps that are less apparent in the biology. Such tiling has been better captured by some efficient coding models (Ocko et al., 2018; Jun et al., 2021, 2022; Karkli and Simoncelli, 2011; Roya et al., 2021; Singer et al., 2023). However, that none of these models had the additional constraint of being spiking, and beyond our non-predictive encoding model, it remains to be seen how different instantiations of efficient coding/compression with spiking would perform. Nevertheless, that we do see tiling to some degree despite our temporal prediction model objective focusing on temporal rather than spatial aspects is notable. Training the temporal prediction model with more units or with additional efficiency constraints, such as on physical space, may enable more even and well-spaced tiling. Indeed, it may be worth exploring models with an objective combining both efficient coding of current input with temporal prediction of future input."

Reviewer 3

In their revised manuscript, Taylor et al. respond to an extensive list of critiques from myself and other reviewers that all cluster around several key topics. In particular, reviewers found that some model aspects appeared ad hoc, the authors failed to discuss several important caveats and related papers, and too little effort was made to distinguish what temporal prediction and recurrence uniquely contribute to the results.

I found the authors' responses reasonable and a bit unsatisfying. The addition of ablation experiments for the recurrent connections are welcome and shed light on several concerns raised by reviewers, and clarifications about the relation between this recurrence and biology have been made at key points. On the other hand, several concerns raised by reviewers about conceptual issues, particularly relations to previous studies, have been relegated to the discussion, while the introduction remains largely unchanged.

On balance, however, the authors have succeeded in better contextualizing their findings and discussing potential limitations of their model, and in this sense my concerns have been addressed. All models are necessarily limited, and the work now better represents the pros and cons of this particular approach.

Reply: We thank the reviewer for their valuable input. We have now modified the Introduction slightly to better contextualize our findings in relation to previous studies and make limitations evident. We

have also slightly reorganized the text of the Results and Discussion in places to make it more concise. See our reply to Reviewer #2.

A few small points:

Reviewer Point P 3.1 — - p4, top: I appreciate the authors comments in the rebuttal addressing the practical points that motivate choosing a fixed time interval for prediction, but in the current manuscript, this number, 128 ms, appears in the results with no explanation or justification. The authors must make some effort to justify this choice to readers, since it is critical to the definition and success of the model.

Reply: This was empirically chosen as the value that gives high prediction for natural inputs. We have now modified the sentence that first introduces this value to read:

"Unless otherwise mentioned, for the predictive model, we report our results using the model optimized for prediction of $T_{\text{off}} = 128$ms into the future as this value gives high prediction of responses to natural scenes across multiple species (see below)."

Also, in the section relating to Figure 6c we now say:

"Hence, it can be seen that the value of T_{off} at 128ms used in previous figures is justified as a value that gives high response prediction across multiple species."

Reviewer Point P 3.2 — - Extended Data Fig 5 caption: "textbfa" should be "**a**", as well as for panel d.

Reply: Thank you, this is now corrected.

Reviewer #3 (Remarks on code availability):

Reviewer Point P 3.3 — Notebooks in codebase define paths relative to a 'root' variable that expects to find/place results in 'PycharmProjects/RetinalModel', which may not exist. Also, [this line](https://github.com/webstorms/RetinalModel/blob/main/scripts/model/fits.py#L4) appears to reference a specific path that may not exist on users' machines. I may have misunderstood something, but I would strongly encourage the authors to verify that their code will run from a clean install per their own setup/install instructions to ensure that all paths work as intended.

Reply: Thank you this is now corrected. We have removed the /PycharmProjects/RetinalModel assumption: root is now auto-resolved from each script's own location and from the notebook working directory, so the repository can be cloned anywhere. The hardcoded /home/datasets/natural in scripts/model/fits.py (and the same default in two analysis classes) has been replaced with root/data/natural; the README now instructs users to place the dataset there.

Reviewer 4

The authors have addressed all of my previous concerns.

Reply: Many thanks for reviewing this manuscruipt.

** See Nature Portfolio's author and referees' website at www.nature.com/authors for information about policies, services and author benefits.

This email has been sent through the Springer Nature Tracking System NY-610A-NPG&MTS
